# FXR-regulated COX6A2 triggers mitochondrial apoptosis of pancreatic β-cell in type 2 diabetes

**DOI:** 10.1038/s41419-024-07302-4

**Published:** 2024-12-20

**Authors:** Lianqi Shao, Xiangchen Kong, Simian Lv, Xingsheng Shu, Xiaosong Ma, Xiaojiao Ai, Dan Yan, Ying Ying

**Affiliations:** 1 Diabetes Institute, the Shenzhen Key Laboratory of Metabolism and Cardiovascular Homeostasis ZDSYS, Shenzhen University Medical School, Shenzhen, PR China; 2 Guangdong Key Laboratory for Biomedical Measurements and U1trasound Imaging, National-Regional Key Technology Engineering Laboratory for Medical U1trasound, School of Biomedical Engineering, Shenzhen University Medical school, Shenzhen, PR China; 3 School of Basic Medicine, Jiamusi University, Jiamusi, PR China

**Keywords:** Acetylation, Metabolic disorders, Predictive markers

## Abstract

Pancreatic β-cell apoptosis plays a crucial role in the development of type 2 diabetes. Cytochrome c oxidase subunit 6A2 (COX6A2) and Farnesoid X Receptor (FXR) have been identified in pancreatic β-cells, however, whether they are involved in β-cell apoptosis is unclear. Here, we sought to investigate the role of FXR-regulated COX6A2 in diabetic β-cell apoptosis. We found that COX6A2 expression was increased in islets from diabetic animals, whereas FXR expression was suppressed. Notably, overexpression of COX6A2 facilitated β-cell apoptosis, whereas its deficiency attenuated this process and ameliorates type 2 diabetes, suggesting a pro-apoptotic role of COX6A2 in β-cells. Mechanistically, increased COX6A2 interacted with and enhanced the expression of voltage-dependent anion channel 1 (VDAC1), thereby promoting the mitochondrial translocation of Bax, leading to the release of cytochrome c from the mitochondria to the cytoplasm and ultimately causing β-cell apoptosis. Moreover, FXR negatively regulated COX6A2 expression through the inhibition of histone acetyltransferase p300 occupancy, diminishing histone H3 acetylation at lysine 27 on the *Cox6a2* promoter. Furthermore, the deficiency of FXR intensified β-cell apoptosis under diabetic situations. Thus, it is probable that in diabetogenic environments, reduced FXR expression contributes to enhanced COX6A2 expression, culminating in β-cell apoptosis. These findings emphasize the essential involvement of the FXR/p300 pathway-controlled COX6A2 in β-cell apoptosis, revealing a previously undiscovered mechanism underlying diabetic β-cell apoptosis.

## Introduction

Type 2 diabetes mellitus (T2DM) is a metabolic disorder characterized by hyperglycemia, resulting from the combined effects of genes and environment. The underlying pathogenesis is primarily due to insulin resistance and dysfunction in the pancreatic islet β-cells, which are the predominant cell type in the islets and have the sole capacity to produce insulin [1, 2]. Previous studies have shown that diabetic patients exhibit reduced β-cell mass and increased rate of β-cell apoptosis, while the rate of cell proliferation appears to be unaffected [3], suggesting that the decrease in β-cell mass in diabetic patients is largely owing to accelerated rates of β-cell death. However, the mechanisms underlying β-cell apoptosis have not been fully uncovered. Unraveling these mechanisms holds great potential importance for the foundational and translational studies pertaining to T2DM.

Farnesoid X Receptor (FXR/Nr1h4), one of the bile acid receptors, is a transcription factor that can be activated or inhibited by a coactivator or corepressor complex, respectively [4–6]. FXR controls the expression of specific genes, a process via binding to FXR response elements (FXRE) on the promoter of the target gene and then recruiting a histone-modifying enzyme, which causes a change in the histone modification at the promoter region, thereby regulating the transcriptional expression of the target gene [7, 8]. FXR has been reported to be widely involved in the entire metabolic process of bile acid synthesis, transport, and reabsorption, as well as regulating blood glucose levels in the body through a variety of direct and indirect ways [9–12]. We have recently highlighted the significant role of FXR in T2DM, where the reduced expression of FXR results in impaired insulin secretion, while the activation of FXR leads to the enhancement of insulin secretion and incretin effect after Roux-en-Y gastric bypass (RYGB) surgery by regulating the expression of TRPA1 or GLP-1R [13, 14]. However, the effect of FXR on β-cell apoptosis remains unclear, although some studies have shown that FXR can inhibit hepatocyte apoptosis [4].

COX6A (Cytochrome c oxidase subunit IV A), one of the 13 subunits of cytochrome c oxidase (also known as mitochondrial respiratory chain complex IV), plays an important role in complex IV expression and enzyme activity [15, 16]. COX6A consists of two subtypes in mammals, COX6A1 (liver-type) and COX6A2 (heart-type), and the latter is expressed in islet β-cell [17–19]. It has been reported that COX6A2 deficient in mice protects against high-fat-diet-induced insulin resistance and obesity [20]. However, whether COX6A2 is involved in the apoptosis of islet β-cell in T2DM remains unknown.

In this study, we show that COX6A2 expression is increased in islets from diabetic animals. Increased COX6A2 promotes β-cell apoptosis by modulating voltage- dependent anion channel 1 (VDAC1)/Bax-mediated release of cytochrome c from mitochondria. Additionally, COX6A2 expression is negatively regulated by FXR through the epigenetic mechanism. Our results reveal that diabetogenic situations likely reduce FXR expression, thereby enhancing COX6A2 expression, which subsequently exaggerates β-cell apoptosis and exacerbates T2DM.

## Results

### COX6A2 enhances the apoptosis of β-cells in diabetes

**Fig. 1:**
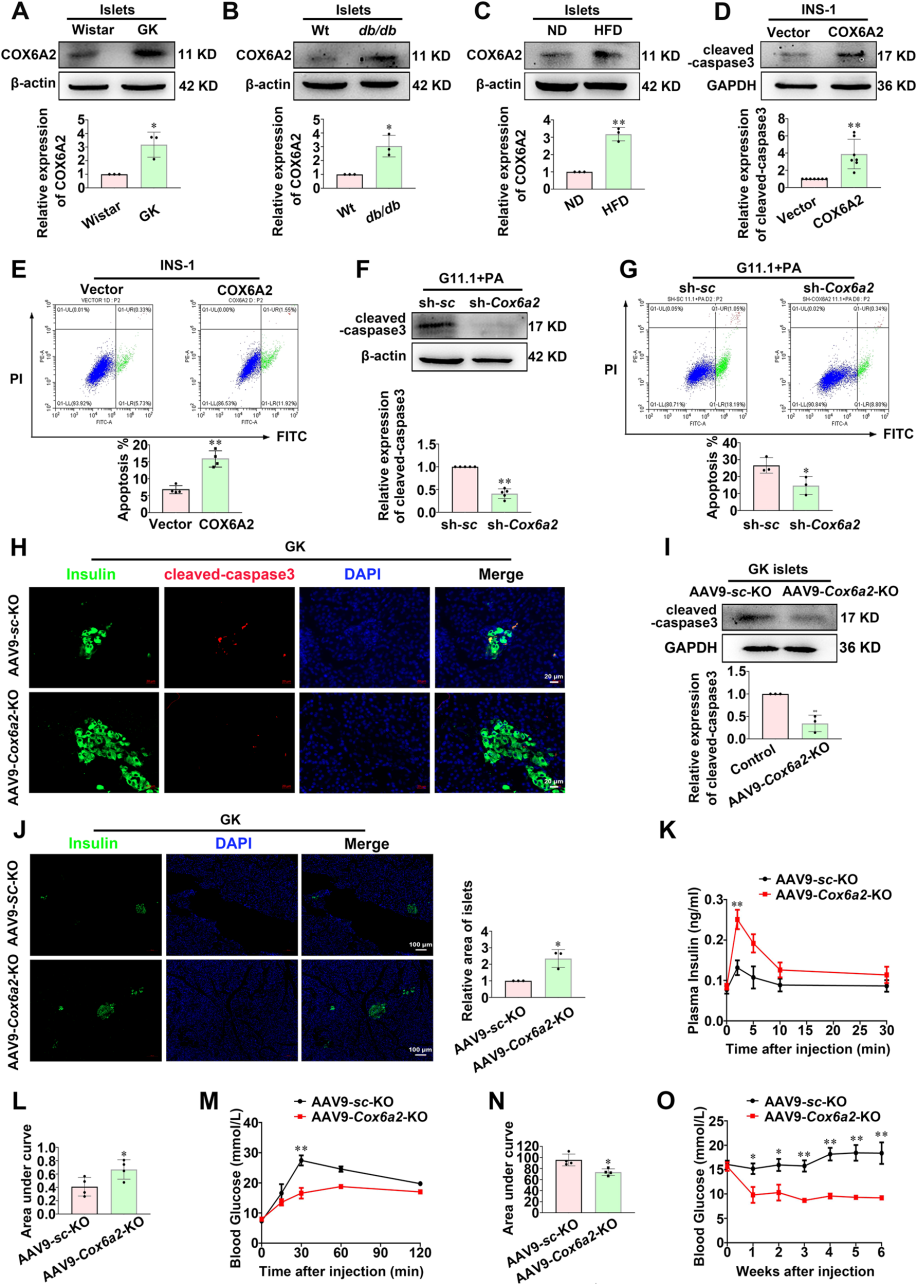
COX6A2 enhances the apoptosis of β-cells in diabetes. **A**–**C** COX6A2 protein was determined (**A**) in islets isolated from Wistar and GK rats, (**B**) in islets isolated from wild-type and db/db mice, and (**C**) in islets isolated from high-fat-diet-induced and normal diet mice, respectively. Bars represent means ± SEM, *n* = 3. *p < 0.05, **p < 0.01 (t-test). **D** Cleaved-caspase3 protein was examined in vector and COX6A2 overexpression INS-1 832/13 cells. Bars represent means ± SEM, *n* = 7. **p < 0.01 (t-test). **E** A flow cytometer was used to determine the apoptosis in vector and COX6A2 overexpression INS-1 832/13 cells. *n* = 4. **p < 0.01 (t-test). **F** Cleaved-caspase3 protein was examined in scramble control and sh-*Cox6a2* INS-1 832/13 cells by treatment with 0.4 mM PA for 48 h. Bars represent means ± SEM, *n* = 5. **p < 0.01 (t-test). **G** A flow cytometer was used to determine the apoptosis in scramble control and sh-*Cox6a2* INS-1 832/13 cells by treatment with 0.4 mM PA for 48 h. *n* = 3. *p < 0.05 (t-test). **H** Representative immunostaining images for cleaved-caspase3 (red), Insulin (green), DAPI (blue), and merge of the three in islets from GK-AAV9-vector and GK-AAV9-*Cox6a2*-KO rats; *n* = 3. Bars represent 20 μm. **I** Cleaved-caspase3 protein was examined in islets from GK-AAV9-vector and GK-AAV9-*Cox6a2*-KO rats. Bars represent means ± SEM, *n* = 3. **p < 0.01 (t-test). **J** Representative immunostaining images for Insulin (green), DAPI (blue), and merge of the two in islets from GK-AAV9-vector and GK-AAV9-*Cox6a2*-KO rats. Bars represent means ± SEM, *n* = 3, Bars represent 100 μm. *p < 0.05 (t-test). **K** Plasma insulin level at 0, 2, 5, 10 and 30 min in GK-AAV9-vector and GK-AAV9-*Cox6a2*-KO rats after intraperitoneal administration of 2 g/kg glucose. Data represent means ± SEM, *n* = 4. **p < 0.01 (t-test). **L** The area under the curves (AUV) was calculated for plasma insulin. Bars represent means ± SEM, *n* = 4. *p < 0.05 (t-test). **M** The glucose level at 0, 15, 30, 60, and 120 min in GK-AAV9-vector and GK-AAV9-*Cox6a2*-KO rats after intraperitoneal administration of 2 g/kg glucose. Data represent means ± SEM, *n* = 4. **p < 0.01 (t-test). **N** The area under the curves (AUV) was calculated for (**M**). Bars represent means ± SEM, *n* = 4. *p < 0.05 (t-test). **O** Glucose level of GK-AAV9-vector and GK-AAV9-*Cox6a2*-KO rats after virus injection. Data represent means ± SEM, *n* = 4. *p < 0.05, **p < 0.01 (t-test).

To determine whether COX6A2 is involved in β-cell apoptosis in diabetes, we investigated the protein expression of COX6A2 in islets in diabetic situations. This revealed that COX6A2 protein expression was increased by 3 folds (*p* < 0.05) in islets from GK rats, db/db mice, and high-fat-diet (HFD)-induced diabetic mice compared to the Wistar rats, C57 mice, and normal diet mice, respectively (Fig. 1A–C). A similar observation was also found in INS-1 832/13 cells treated with 0.4 mM PA for 48 h (Supplementary Fig. S1), which produces a model of high-fat-diet (Palmitic acid)- induced lipotoxicity in vitro, confirming the elevated COX6A2 expression in diabetic β-cells. Then COX6A2 overexpression (Supplementary Fig. S2A) and *Cox6a2* knockdown INS-1 832/13 cells (Supplementary Fig. S2B) were employed to identify whether COX6A2 regulates the apoptosis of β-cells. Ectopic expression of COX6A2 triggered increase in cleaved-caspase3 expression (*p* < 0.01) and apoptotic cell numbers (*p* < 0.01) (Fig. 1D, E), while knockdown of *Cox6a2* led to a significant reduction in cleaved-caspase3 (*p* < 0.01) and apoptotic cell numbers (*p* < 0.05) upon lipotoxicity stress, as compared to the scramble control (Fig. 1F, G).

To further investigate the role of COX6A2 in diabetic β-cell apoptosis in vivo, the AAV9-*Cox6a2*-KO (adeno-associated virus) viral vectors were injected into the pancreas of GK rats via the common bile duct to achieve tissue-specific knockdown of the *Cox6a2* gene. A scrambled shRNA (U6-spgRNA: GCACCCAGTCCGCCCTGAGCAAA) was applied as a control. The administration of AAV9-*Cox6a2*-KO led to a significant decrease in islet COX6A2 protein expression (Supplementary Fig. S2C). This was paralleled with considerably decreased expression of cleaved-caspase3 protein (*p* < 0.01) in the islets from GK rats injected with AAV9-*Cox6a2*-KO, compared to those injected with scramble control (Fig. 1H, I). Concomitantly, the insulin-positive cell area was increased (*p* < 0.05) in AAV9-*Cox6a2*-KO group (Fig. 1J). Therefore, the GK rats administrated with AAV9- *Cox6a2*-KO exhibited enhanced plasma insulin levels (Fig. 1K, L), improved glucose tolerance (Fig. 1M, N). Accordingly, the GK rats administrated with AAV9-*Cox6a2*-KO showed significantly lower blood glucose levels than those with control AAV vectors at 6 weeks post-injection (Fig. 1O). These results suggest that COX6A2 enhances the apoptosis of β-cells in T2DM. There is a limitation in this study that we didn’t measure the RNA level of *Cox6a2* in the islets from GK rats injected with AAV9-*Cox6a2*-KO, compared to those injected with scramble control in order to have enough islets for the COX6A2 protein detection. An all-round assessment of the knockdown’s comprehensiveness will surely improve this study.

**Fig. 2:**
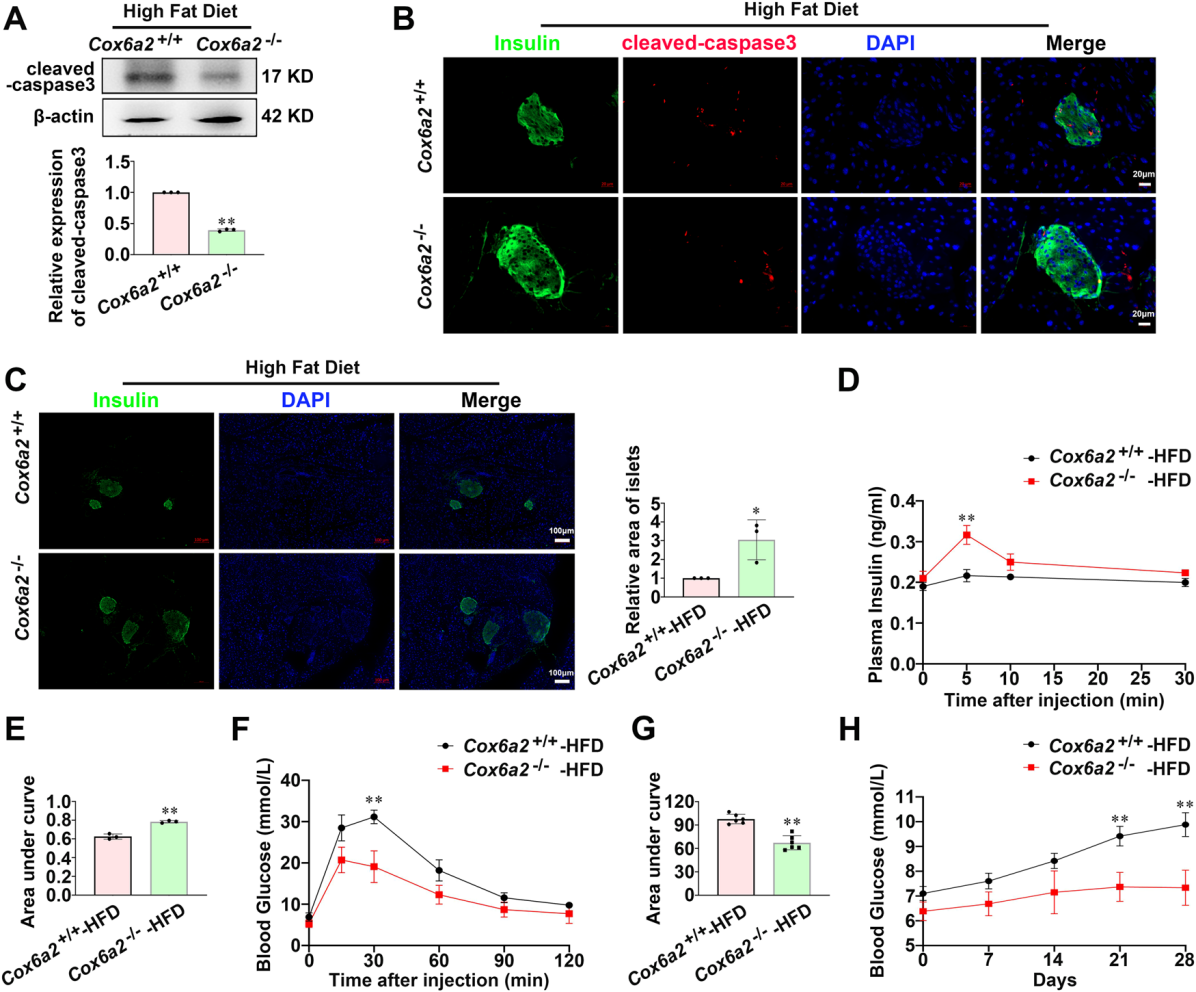
Knockout of *Cox6a2* reduces β-cells apoptosis and improves diabetes symptoms. **A** Cleaved-caspase3 protein was examined in islets from *Cox6a2*^+/+^ and *Cox6a2*^−/−^mice fed with HFD. Bars represent means ± SEM, *n* = 3. **p < 0.01 (t-test). **B** Representative immunostaining images for cleaved-caspase3 (red), Insulin (green), DAPI (blue), and merge of the three in islets from *Cox6a2*^+/+^ and *Cox6a2*^−/−^ mice fed with HFD; *n* = 3. Bars represent 20 μm. **C** Representative immunostaining images for Insulin (green), DAPI (blue), and merge of the two in islets from *Cox6a2*^+/+^ and *Cox6a2*^−/−^ mice fed with HFD. Bars represent means ± SEM, *n* = 3, Bars represent 100 μm. *p < 0.05 (t-test). **D** Plasma insulin level at 0, 5, 10, and 30 min in *Cox6a2*^+/+^ and *Cox6a2*^−/−^ mice fed with HFD after intraperitoneal injection of 2 g/kg glucose. Data are means ± SEM, *n* = 3. **p < 0.01 (t-test). **E** The area under the curves (AUC) was calculated for plasma insulin. Bars represent means ± SEM, *n* = 3. **p < 0.01 (t-test). **F** The glucose level at 0, 15, 30, 60, 90, and 120 min in *Cox6a2*^+/+^ and *Cox6a2*^−/−^ mice fed with HFD after intraperitoneal injection of 2 g/kg glucose. Data represent means ± SEM, *n* = 6. **p < 0.01 (t-test). **G** The area under the curves (AUC) was calculated for (**F**). Bars represent means ± SEM, *n* = 6. **p < 0.01 (t-test). **H** The glucose level of *Cox6a2*^+/+^ and *Cox6a2*^−/−^ mice fed with HFD. Data represent means ± SEM, *n* = 6. **p < 0.01 (t-test).

To corroborate the aforementioned findings, we further generated wild-type (*Cox6a2*^+/+^) and *Cox6a2* knockout (*Cox6a2*^−/−^) mice (Supplementary Fig. S2D) and examined the expression of islet cleaved-caspase3 and insulin-positive cell area in *Cox6a2*^+/+^ and *Cox6a2*^−/−^ mice fed with HFD for 28 days. As shown in Fig. 2A, B, a notable decrease in cleaved-caspase3 protein was observed in the islets from *Cox6a2*^−/−^ mice under HFD (*p* < 0.01). Thus, the insulin-positive cell area was enlarged (*p* < 0.05) in *Cox6a2*^−/−^-HFD group mice (Fig. 2C). Consequently, the *Cox6a2*^−/−^-HFD mice exhibited enhanced plasma insulin levels (Fig. 2D, E), which was parallelled by improved glycemic controls (Fig. 2F, G). Therefore, the *Cox6a2*^−/−^-HFD group mice maintained a lower blood glucose level of ~7 mmol/L (*p* < 0.01), compared to the *Cox6a2*^+/+^-HFD mice over 28 days after HFD feeding (Fig. 2H). These results thus suggest that knockout of *Cox6a2* reduces HFD-induced β-cell apoptosis and ameliorates HFD-induced diabetes associated symptoms.

### COX6A2 triggers mitochondria-dependent apoptosis of β-cells via regulation of VDAC1

**Fig. 3:**
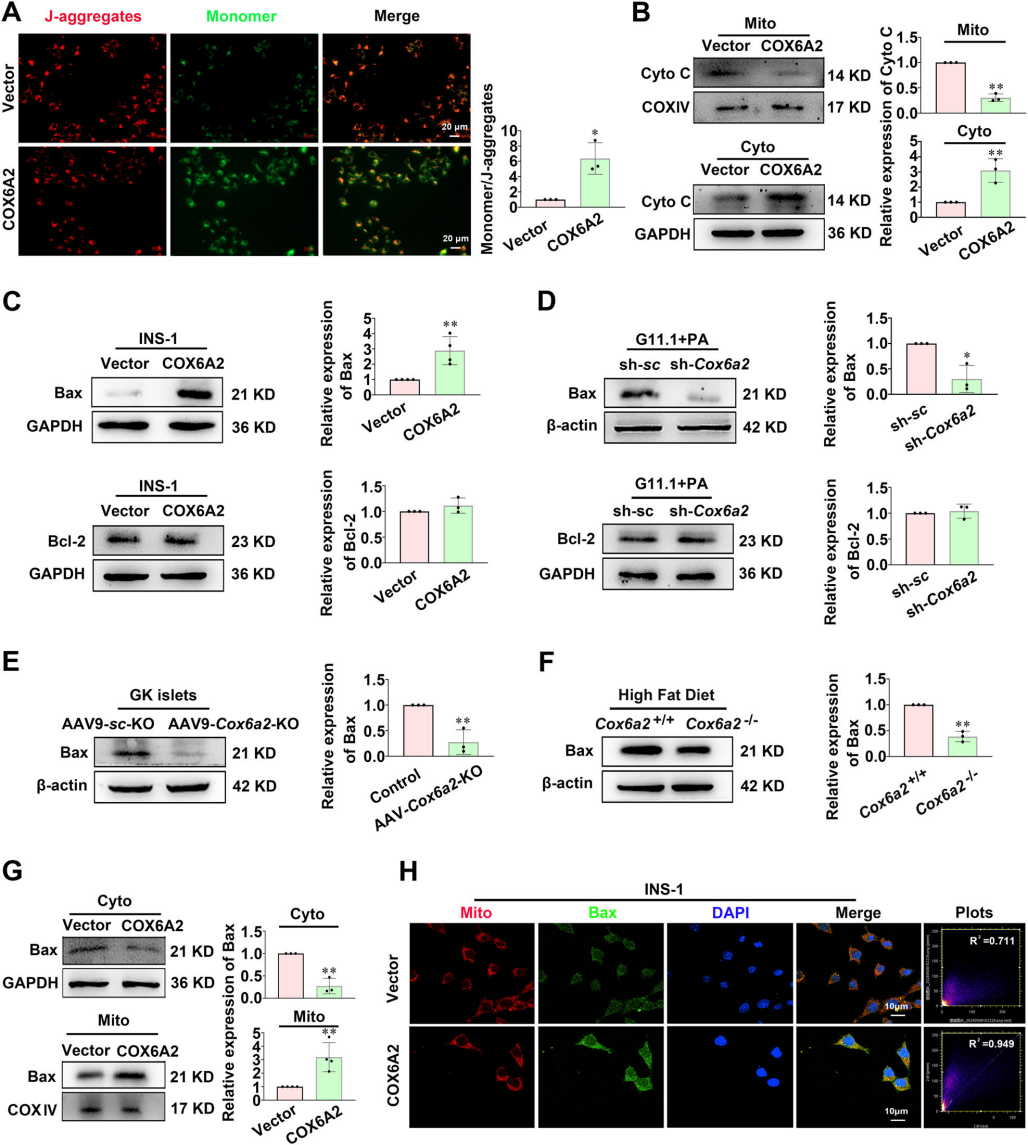
COX6A2 promotes the mitochondrial translocation of Bax and the release of cytochrome c from mitochondria in β-cells. **A** Mitochondrial membrane potential was determined in vector and COX6A2 overexpression INS-1 832/13 cells by JC-1. Bars represent means ± SEM, *n* = 3, Bars represent 20 μm. *p < 0.05 (t-test). **B** The distribution of cytochrome c in mitochondria and cytoplasm was determined in vector and COX6A2 overexpression INS-1 832/13 cells. Bars represent means ± SEM, *n* = 3. **p < 0.01 (t-test). **C**, **D** Bax and Bcl-2 protein expression was examined in vector and COX6A2 overexpression INS-1 832/13 cells (**C**), or scramble control and sh-*Cox6a2* INS-1 832/13 cells treated with 0.4 mM PA for 48 h (**D**). *n* = 4 (vector and COX6A2). **p < 0.01 (t-test), *n* = 3 (scramble control and sh-*Cox6a2*). *p < 0.05 (t-test). **E**, **F** The protein expression of Bax was determined in islets from GK-AAV9-vector and GK-AAV9-*Cox6a2*-KO rats (**E**), and islets from *Cox6a2*^+/+^ and *Cox6a2*^−/−^ mice fed with HFD (**F**). *n* = 3. **p < 0.01 (t-test). **G** The distribution of Bax in cytoplasm and mitochondria was determined in vector and COX6A2 overexpression INS-1 832/13 cells. Bars represent means ± SEM, *n* = 3 (cytoplasmic protein), *n* = 4 (mitochondrial protein). **p < 0.01 (t-test). **H** Representative immunostaining images for mitochondria (red), Bax (green), and DAPI (blue) in vector and COX6A2 overexpression INS-1 832/13 cells; *n* = 3, Bars represent 10 μm.

We explored the cellular mechanism by which COX6A2 promotes β-cell apoptosis. Given that COX6A2 is a subunit of mitochondrial complex IV, we hypothesized that COX6A2 might induce β-cell apoptosis via a mitochondria-dependent pathway. Consequently, we analyzed mitochondrial-related indicators linked to apoptosis. As shown in Fig. 3A, B, COX6A2 overexpression cells exhibited a decreased mitochondrial transmembrane potential (ΔΨm) (*p* < 0.05) and facilitated the release of cytochrome c from the mitochondria to the cytoplasm compared to vector cells. We also examined the apoptosis regulatory genes Bcl-2 and Bax. The Bax protein level significantly increased (*p* < 0.01) in COX6A2 overexpression cells but decreased markedly (*p* < 0.05) in *Cox6a2* knockdown cells under lipotoxicity challenge (Fig. 3C, D). Consistent with the in vitro findings, Bax was significantly decreased in the islets from GK rats injected with AAV9-*Cox6a2*-KO virus and in those from *Cox6a2*^−/−^-HFD mice compared to the respective controls (Fig. 3E, F). We further investigated the subcellular distribution of Bax and found that the Bax protein level is lower in cytoplasm extracts but higher in mitochondrial extracts in COX6A2 overexpression cells compared to vector cells (Fig. 3G). The immunostaining assay also indicated stronger colocalization signals of Bax and mitochondria in COX6A2 overexpression cells than in vector cells (Fig. 3H). These data suggest that COX6A2 not only promotes increased protein levels of Bax but also facilitates its mitochondrial translocation. Interestingly, there was no significant difference in the expression of Bcl-2 (Fig. 3C, D).

**Fig. 4:**
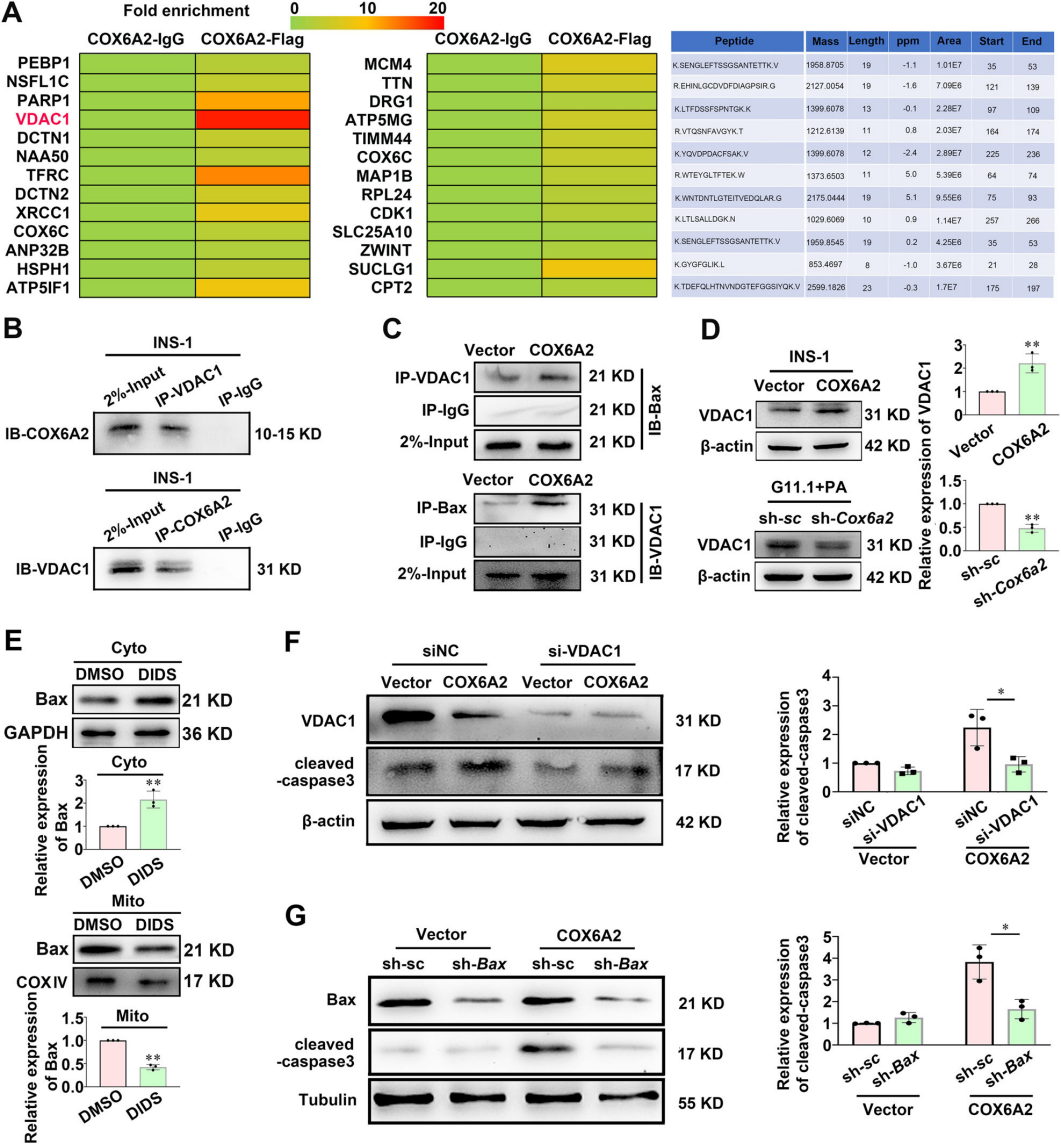
Increased COX6A2 induces β-cell apoptosis via regulation of VDAC1. **A** The outcome of mass spectrometry analysis regarding proteins that interacted with COX6A2. **B** Anti-COX6A2 and anti-VDAC1 antibodies were used to carry out a protein pull-down assay of VDAC1 and COX6A2 in INS-1 832/13 cells. **C** Anti-Bax and anti-VDAC1 antibodies were used for VDAC1 and Bax protein pull-down in vector and COX6A2 overexpression INS-1 832/13 cells. **D** VDAC1 protein was determined in vector and COX6A2 overexpression INS-1 832/13 cells (upper panel), or scramble control and sh-*Cox6a2* INS-1 832/13 cells treated with 0.4 mM PA for 48 h (lower panel), Bars represent means ± SEM, *n* = 3. **p < 0.01 (t-test). **E** The distribution of Bax in the cytoplasm and mitochondria was determined in INS-1 832/13 cells treated with DMSO or 200 μM DIDS for 2 h. Bars represent means ± SEM, *n* = 3. **p < 0.01 (t-test). **F** The vector and COX6A2 overexpression INS-1 832/13 cells were transfected with either control siRNA (siNC) or siRNA target against *Vdac1* mRNA (si-VDAC) for 48 h, followed by the examination of protein levels of VDAC1 and cleaved-caspase3. Bars represent means ± SEM, *n* = 3. *p < 0.05 (t-test). **G** The level of cleaved-caspase3 in vector and COX6A2 overexpression INS-1 832/13 cells simultaneously transduced with scramble or shRNA targeted against Bax mRNA. Data are means ± SEM, *n* = 3. *p < 0.05 (t-test).

To investigate the specific mechanism by which COX6A2 facilitates the mitochondrial translocation of Bax, we employed mass spectrometry to identify proteins interacting with COX6A2 following the pull-down of all binding proteins. Among the identified partners, we noted VDAC1, a mitochondrial protein crucial for apoptosis driven by the mitochondria [21, 22] (Fig. 4A). Co-immunoprecipitation (Co-IP) experiments corroborated that COX6A2 associates with VDAC1 (Fig. 4B, Supplementary Fig. S9). Additionally, the interaction between VDAC1 and Bax is further strengthened by the overexpression of COX6A2 (Fig. 4C). Furthermore, examination of VDAC1 expression revealed that overexpression of COX6A2 in INS-1 832/13 cells resulted in a significant elevation in both VDAC1 protein (*p* < 0.01) and mRNA (*p* < 0.01) levels (Fig. 4D; Supplementary Fig. S3). Conversely, VDAC1 protein levels significantly decreased (*p* < 0.01) in *Cox6a2* knockdown cells under lipotoxic conditions (Fig. 4D). Collectively, these findings suggest that COX6A2 overexpression enhances the interaction between VDAC1 and Bax, thereby increasing the mitochondrial translocation of Bax. Interestingly, COX6A2 overexpression cells treated with 200 μM DIDS (an inhibitor of VDAC1) for 2 h induced an increase in cytoplasmic Bax and a concurrent decrease in mitochondrial Bax (Fig. 4E). To investigate the role of VDAC1 in COX6A2-increased β-cell apoptosis, the expression of cleaved-caspase3 was determined in COX6A2 overexpression cells that were either transfected with VDAC1 siRNA or treated with DIDS. As shown in Fig. 4F and Supplementary Fig. S4, the expression of cleaved-caspase3 was substantially reduced (*p* < 0.05) in COX6A2 overexpression cells after transfection with si-VDAC1 or treatment with DIDS. To confirm the role of Bax in COX6A2-induced β-cell apoptosis, we knocked down Bax in both vector and COX6A2 overexpression INS-1 832/13 cells. As shown in Fig. 4G, Bax knockdown markedly attenuated the elevated levels of cleaved-caspase3 induced by COX6A2 overexpression. These findings indicate that COX6A2 facilitates the translocation of Bax from the cytoplasm to the mitochondria by interacting with VDAC1, ultimately leading to β-cell apoptosis.

### FXR inhibits the *Cox6a2* expression in β-cells

**Fig. 5:**
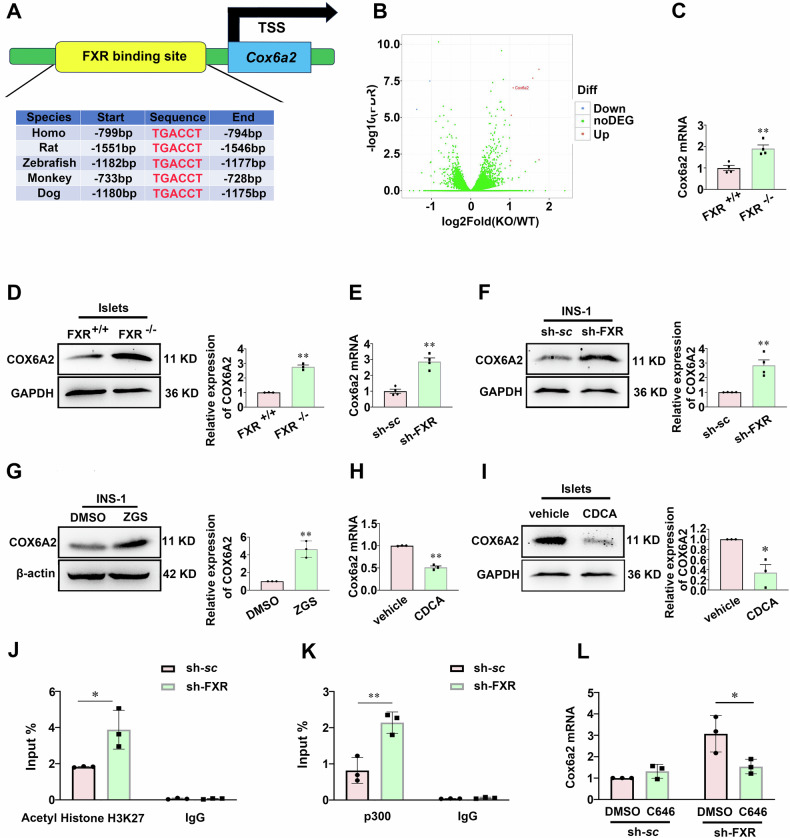
FXR regulates COX6A2 expression in β-cells. **A** The potential binding site of FXR in the promoter region of *Cox6a2* gene was analyzed with the bioinformatics database JASPAR. **B** The result of RNA-seq in islets from FXR^+/+^ and FXR^−/−^ mice. **C**, **D** The expression of COX6A2 mRNA (**C**) and protein (**D**) were determined in islets from FXR^+/+^ and FXR^−/−^ mice. Bars represent means ± SEM, *n* = 4 (**C**) or 3 (**D**). **p < 0.01 (t-test). **E**, **F** qPCR (**E**) and Western blotting (**F**) analysis of COX6A2 mRNA (**E**) and protein (**F**) expression in scramble control and sh-FXR INS-1 832/13 cells. Bars represent means ± SEM, *n* = 4. **p < 0.01 (t-test). **G** COX6A2 protein expression was determined in INS-1 832/13 cells treated with DMSO or 20 μM Z-guggulsterone (ZGS) for 48 h. Bars represent means ± SEM, *n* = 3. **p < 0.01 (t-test). **H**, **I** COX6A2 mRNA (**H**) and protein (**I**) were examined in islets isolated from SD rats. The rats were subjected to intraperitoneal injection of CDCA at a dose of 20 mg/kg for 2 weeks. Bars represent means ± SEM, *n* = 3 rats per group. *, *p* < 0.05; **p < 0.01 (t-test). **J**, **K** ChIP assays were performed to determine the acetylation of histone H3 at lysine 27 (**J**), and the occupancy of p300 (**K**), at the *Cox6a2* promoter in the scramble and sh-FXR INS-1 832/13 cells. Bars represent means ± SEM, *n* = 3. *p < 0.05, **p < 0.01 (t-test). **L** The level of *Cox6a2* mRNA was examined in the scramble and sh-FXR INS-1 832/13 cells treated with DMSO or 10 μM C646 for 48 h. Data are means ± SEM, *n* = 3. *p < 0.05 (t-test).

An analysis of the JASPAR bioinformatics database identified potential FXR binding sites in the promoter region of the *Cox6a2* gene (Fig. 5A). Additionally, RNA sequencing (RNA-seq) was used to investigate differentially expressed genes in the islets from FXR^+/+^ and FXR^−/−^ mice. Intriguingly, RNA-seq revealed an increased transcription of *Cox6a2* in FXR^−/−^ mice (Fig. 5B). This upregulation was confirmed through elevated mRNA and protein levels in islets from FXR^−/−^ mice (Fig. 5C, D). Similar findings were observed in INS-1 832/13 cells upon FXR knockdown or inhibition (Fig. 5E–G). Conversely, intraperitoneal injection of SD rats with the endogenous FXR agonist CDCA for 14 days resulted in significant reductions in *Cox6a2* mRNA and protein levels (Fig. 5H, I). Likewise, INS-1 832/13 cells treated with the FXR agonist GW4064 showed a notable decrease in *Cox6a2* expression (Supplementary Fig. S5A, B). These findings suggest that FXR negatively regulates *Cox6a2* expression in β-cells. Since FXR regulates gene expression through histone modification, we investigated the epigenetic mechanisms by which FXR controls *Cox6a2* expression. Remarkably, we observed that FXR knockdown in INS-1 832/13 cells significantly increased the acetylation of histone H3 at lysine 27 (ACH3K27), a modification associated with enhanced gene transcription, at the *Cox6a2* promoter (Fig. 5J). Further, FXR knockdown heightened the recruitment of p300, a histone acetyltransferase responsible for H3K27 acetylation [23, 24], to the *Cox6a2* promoter (Fig. 5K). Crucially, administering the specific p300 inhibitor, C646, reversed the FXR knockdown-induced rise in *Cox6a2* mRNA levels (Fig. 5L). These findings suggest that FXR suppresses *Cox6a2* transcription in β-cells by limiting p300 recruitment and subsequently decreasing H3K27 acetylation at the *Cox6a2* promoter.

### FXR inhibits the apoptosis of β-cells in diabetes

**Fig. 6:**
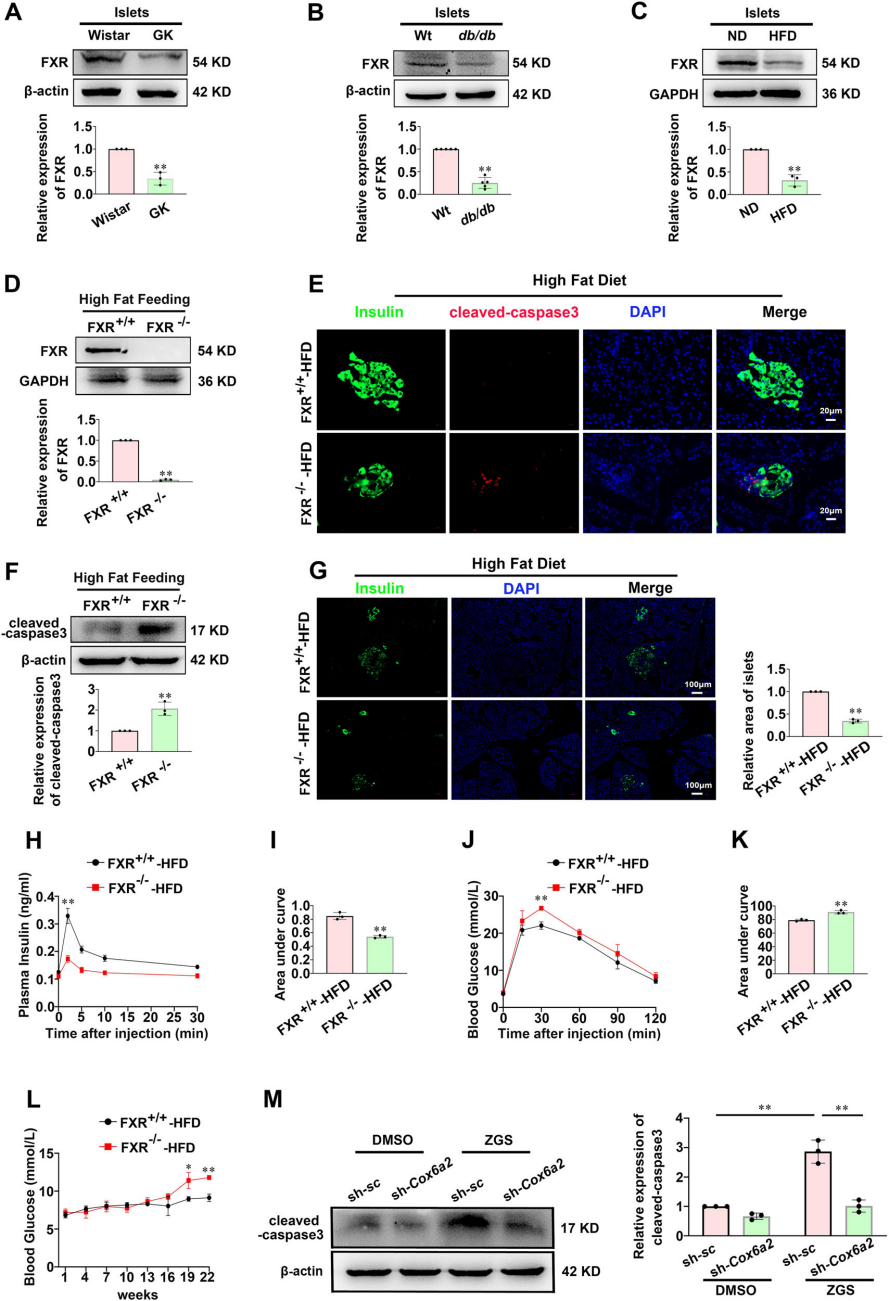
FXR inhibits the apoptosis of β-cells in diabetes. **A**–**C** The protein expression of FXR was determined in islets isolated from Wistar and GK rats (**A**), in islets isolated from wild-type and db/db mice (**B**), and in islets isolated from high-fat-diet-induced and normal diet mice (**C**), respectively. Bars represent means ± SEM, *n* = 3–5. **p < 0.01 (t-test). **D** FXR protein expression was examined in islets from FXR^+/+^ and FXR^−/−^ mice fed with HFD. Bars represent means ± SEM, *n* = 3. **p < 0.01 (t-test). **E** Representative immunostaining images for cleaved-caspase3 (red), Insulin (green), DAPI (blue), and merge of the three in islets from FXR^+/+^ and FXR^−/−^ mice fed with HFD; *n* = 4. Bars represent 20 μm. **F** The protein level of cleaved-caspase3 was examined in islets from FXR^+/+^ and FXR^−/−^ mice fed with HFD. Bars represent means ± SEM, *n* = 3. **p < 0.01 (t-test). **G** Representative immunostaining images for Insulin (green), DAPI (blue), and merge of the two in islets from FXR^+/+^ and FXR^−/−^ mice fed with HFD. Bars represent means ± SEM, *n* = 3, Bars represent 100 μm. **p < 0.01 (t-test). **H** Plasma insulin level at 0, 2, 5, 10, and 30 min in FXR^+/+^ and FXR^−/−^ mice fed with HFD after intraperitoneal injection of 2 g/kg glucose. Bars represent means ± SEM, *n* = 3. **p < 0.01 (t-test). **I** The area under the curves (AUC) was calculated for plasma insulin. Bars represent means ± SEM, *n* = 3. **p < 0.01 (t-test). **J** The glucose level at 0, 15, 30, 60, 90, and 120 min in FXR^+/+^ and FXR^−/−^ mice fed with HFD after intraperitoneal administration of 2 g/kg glucose. Bars represent means ± SEM, *n* = 3. **p < 0.01 (t-test). **K** The area under the curves (AUC) was calculated for IPGTT. Bars represent means ± SEM, *n* = 3. **p < 0.01 (t-test). **L** The glucose level of FXR^+/+^ and FXR^−/−^ mice after fed with HFD. Bars represent means ± SEM, *n* = 3. *p < 0.05, **p < 0.01 (t-test). **M** The level of cleaved-caspase3 protein was examined in the scramble and sh-*Cox6a2* INS-1 832/13 cells treated with DMSO or 20 μM ZGS for 48 h, respectively. Bars represent means ± SEM, n = 3. **p < 0.01 (t-test).

To explore the impact of FXR on β-cells apoptosis in T2DM, we initially assessed its expression levels and found a significant reduction: ~70% (*p* < 0.01), ~80% (*p* < 0.01) and ~70% (*p* < 0.01) in islets from GK rats, db/db mice and high-fat-diet-induced diabetic mice, respectively, compared to the Wistar rats, C57 mice and normal diet mice (Fig. 6A–C; Supplementary Fig. S6A). Similarly, the expression of FXR was reduced by ~70% (*p* < 0.01) in INS-1 832/13 cells upon lipotoxicity challenge, as compared to the control group (Supplementary Fig. S6B). We then employed FXR knockdown (Supplementary Fig. S7A), inhibition, and overexpression in INS-1 832/13 cells (Supplementary Fig. S7B) to determine whether FXR regulates β-cell apoptosis. Knockdown or inhibition of FXR triggered an increase in cleaved-caspase3 expression (*p* < 0.05) and apoptotic cell numbers (*p* < 0.01) (Supplementary Fig. S8A–C), while overexpression of FXR significantly reduced cleaved-caspase3 expression (*p* < 0.01) and apoptotic cell numbers (*p* < 0.01) under lipotoxic stress conditions compared to the vector control (Supplementary Fig. S8D, E). Notably, islets from FXR^−/−^ mice (Fig. 6D) fed with HFD exhibited a similar increase in cleaved-caspase3 protein levels (*p* < 0.01), concomitant with decreased insulin-positive cell area (*p* < 0.01), as compared with those from FXR^+/+^ group fed the same diet (Fig. 6E–G). Therefore, FXR^−/−^ mice fed with HFD displayed reduced plasma insulin levels and impaired glucose tolerance compared with the control FXR^+/+^ HFD group (Fig. 6H–K). Accordingly, blood glucose levels in FXR^−/−^ mice with HFD were significantly higher after 19 weeks of feeding, reaching approximately 12.5 mmol/L at 22 weeks, while the blood glucose level in FXR^+/+^ mice on the same diet consistently stayed below ~10 mmol/L (Fig. 6L). To determine whether FXR protects the β-cells from apoptosis by repressing *Cox6a2* expression, the expression of cleaved-caspase3 was evaluated in both scramble and *Cox6a2* knockdown cells treated with the FXR inhibitor ZGS for 48 h. As shown in Fig. 6M, treatment with ZGS substantially increased the expression of cleaved-caspase3 (*p* < 0.01) in scramble INS-1 832/13 cells. However, *Cox6a2* knockdown abolished the ZGS-enhanced cleaved-caspase3 level compared to the scramble group. These results indicate that FXR protects β-cell from apoptosis by repressing *Cox6a2* expression under diabetic conditions.

## Discussion

**Fig. 7:**
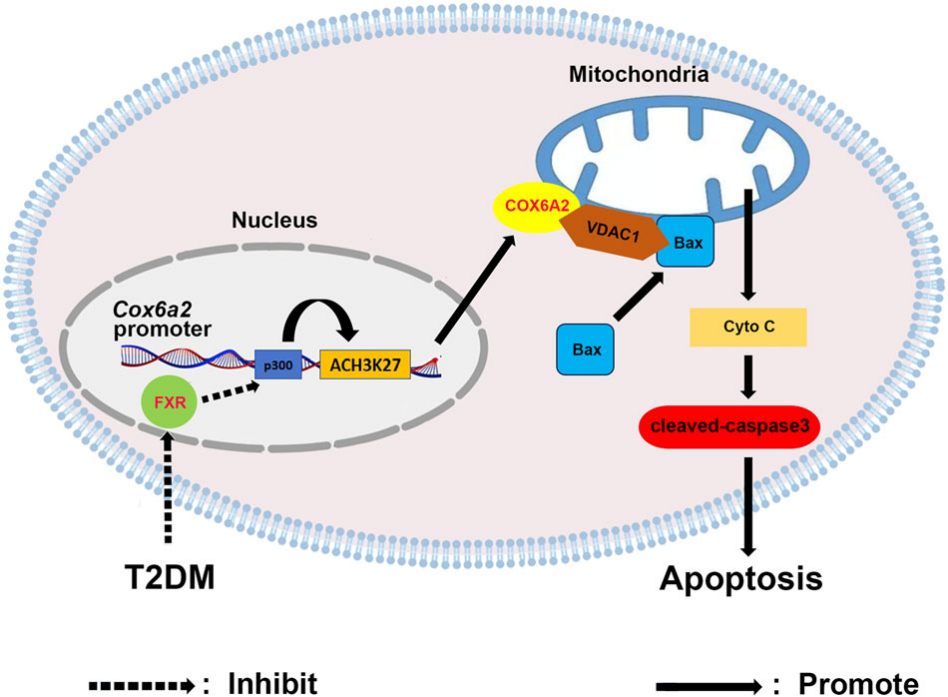
The schematic representation of the molecular mechanism. Schematic representation of the molecular mechanism of FXR/COX6A2-mediated β-cell apoptosis in diabetes. See text for details.

In this study, we found that FXR negatively regulates β-cell apoptosis by repressing *Cox6a2* transcription. Specifically, FXR achieves this by diminishing p300-mediated acetylation of H3K27 at the *Cox6a2* promoter. Importantly, we further revealed that COX6A2 facilitates the mitochondrial translocation of Bax by interacting with VDAC1, which in turn leads to islet β-cell apoptosis. These findings suggest that the diabetogenic situations-reduced FXR leads to the increase of COX6A2, thereby inducing β-cell apoptosis through a mitochondria-dependent pathway, ultimately contributing to the exacerbation of type 2 diabetes (Fig. 7).

COX6A2 is one of the 13 subunits of cytochrome c oxidase. Deficiency of *Cox6a2* in mice has shown protection against insulin resistance and obesity induced by high-fat diets [20]. COX-2, another subunit of cytochrome c oxidase, has been implicated in the apoptosis of human colon cancer, non-small-cell lung cancer cells, and endothelial cells [25–27]. However, there is no existing evidence that COX6A2 plays a role in regulating β-cell apoptosis. Based on our findings, we present the first evidence that COX6A2 promotes β-cell apoptosis in diabetes. This claim is supported by three key pieces of evidence. First, elevated expression of COX6A2 was observed in islets from diabetic animals (Fig. 1A–C). Second, overexpression of COX6A2 led to increased expression of cleaved-caspase3 in INS-1 832/13 cells, which was reduced in sh-*Cox6a2* INS-1 832/13 cells under lipotoxic stress, as well as in islets from GK rats injected with AAV9- *Cox6a2*-KO virus, and in islets from *Cox6a2*^−/−^ mice fed with high-fat diet when compared to their respective controls (Fig. 1D, F, H, I; Fig. 2A, B). Third, COX6A2 overexpressing INS-1 832/13 cells exhibited a higher number of apoptotic cells, whereas *Cox6a2* knockdown cells displayed a reduced number of apoptotic cells under lipotoxic stress (Fig. 1E, G) compared to their controls.

To investigate how COX6A2 triggers β-cell apoptosis, we utilized mass spectrometry to identify proteins interacting with COX6A2 in COX6A2 overexpressing INS-1 832/13 cells. Among the proteins, VDAC1 exhibited the highest fold enrichment (Fig. 4A). VDAC1 plays a critical role in regulating mitochondrial function and can induce apoptosis by facilitating the release of cytochrome c through its oligomerization and formation of a channel within the VDAC1 homo-oligomer [28]. Our findings reveal that COX6A2 promotes β-cell apoptosis via modulation of VDAC1, supported by several key observations. First, the Co-IP assay confirmed the interaction between COX6A2 and VDAC1 (Fig. 4B, Supplementary Fig. S9). Second, the protein level of VDAC1 was notably elevated in COX6A2 overexpressing INS-1 832/13 cells but decreased in COX6A2 knockdown cells under lipotoxic stress (Fig. 4D). Third, either silence or inhibition of VDAC1 diminished the elevated level of cleaved-caspase3 protein induced by COX6A2 overexpression (Fig. 4F; Supplementary Fig. S4).

It is well established that VDAC1 can interact with Bax, which forms oligomers in the mitochondrial outer membrane in response to apoptotic signals. This interaction facilitates the release of cytochrome c into the cytoplasm, thereby promoting apoptosis. VDAC1 works synergistically with Bax to increase the permeability of the mitochondrial outer membrane, jointly fostering the apoptotic process. Reduction of VDAC1 expression via RNA interference significantly diminishes Bax activation, and inhibits mitochondrial outer membrane permeability, thereby precluding cytochrome c release and caspase 3 activation [29–31]. This ultimately leads to suppressed cell apoptosis. Notably, our data reveal that overexpression of COX6A2 increases the interaction between VDAC1 and Bax (Fig. 4C), enhancing mitochondrial translocation of Bax, an effect reduced by VDAC1 inhibition (Fig. 4E). Furthermore, the knockdown of Bax counteracted the elevated levels of cleaved-caspase3 induced by COX6A2 overexpression (Fig. 4G). This underscores the critical role of Bax in COX6A2- mediated β-cell apoptosis. Nonetheless, the precise mechanisms by which COX6A2 affects VDAC1 and Bax expression require further investigation.

A study [17] has reported a decrease in the mRNA levels of *Cox6a2* in islets from db/db mice. Contrarily, our findings show an increase in the COX6A2 protein levels in islets from db/db mice compared to the control group (Fig. 6B). The discrepancy may stem from differences in experimental conditions and the levels at which the *Cox6a2* gene was assessed.

FXR is a transcription factor that regulates the transcriptional expression of target genes through epigenetic mechanisms [32]. We demonstrate that *Cox6a2* is a target gene negatively regulated by FXR in β-cells. The following evidence supports this concept: First, FXR binding sites (FXRE) are present at the *Cox6a2* promoter (Fig. 5A). Second, activation of FXR by CDCA or GW4064 reduces the expression of *Cox6a2* mRNA and protein (Fig. 5H, I; Supplementary Fig. S5A, B). Third, a deficiency in FXR leads to increased *Cox6a2* expression (Fig. 5C–F). A key finding of this study is that FXR knockdown promotes the recruitment of p300 to the *Cox6a2* promoter (Fig. 5K), which results in increased ACH3K27 at the promoter (Fig. 5J). This acetylation is associated with enhanced transcription of the *Cox6a2* gene, as ACH3K27 is known to be a marker of gene activation [24, 33]. Furthermore, the inability of FXR knockdown to increase *Cox6a2* expression in the presence of p300 inhibitor (Fig. 5L) further underscores the critical role of p300 in mediating FXR-dependent regulation of *Cox6a2* expression. Previous studies have indicated that FXR can suppress hepatocyte apoptosis [4]. In this research, we expand on these findings by demonstrating that FXR inhibits the apoptosis of β-cells by repressing the transcription of *Cox6a2* in diabetic situations. This hypothesis is supported by several lines of evidence. First, FXR expression was found to be decreased in islets from diabetic animals (Fig. 6A–C). Correspondingly, FXR deficiency resulted in increased β-cell apoptosis both in vivo and in vitro (Fig. 6E, F; Supplementary Fig. S8A, B). Conversely, the overexpression of FXR led to reduced β- cell apoptosis under lipotoxic conditions (Supplementary Fig. S8D, E). Additionally, while the inhibition of FXR induced apoptosis in scramble INS-1 832/13 cells, it did not have the same effect in *Cox6a2* knockdown cells (Fig. 6M). This suggests that COX6A2 may mediate FXR-regulated β-cell apoptosis.

Collectively, our findings indicate that increased COX6A2 levels may enhance β-cell apoptosis through the modulation of VDAC1-mediated cytochrome c release from the mitochondria. This rise in COX6A2 expression might result from the reduced expression of FXR in diabetes. Diabetic conditions likely drive β-cell apoptosis by modulating the FXR/p300/COX6A2 pathway. These insights reveal a novel regulatory mechanism for β-cell apoptosis and highlight COX6A2 as a possible therapeutic target for type 2 diabetes. Unfortunately, there is no human study confirming the changes observed in COX6A2 expression in islets from diabetic animals now. And we can not obtain any human diabetic or normal pancreatic tissue currently. It can be a limitation to our study. Therefore, further research in human will certainly improve our study greatly.

## Materials and methods

### Experimental animals and isolation of islets

Male Wistar and Goto-Kakizaki (GK) rats (Blood glucose is more than 15 mmol/L) aged 8 weeks were purchased from SLRC (Shanghai, China). Male Sprague Dawley (SD) rats (aged 6 weeks) were purchased from Guangdong Medical Laboratory Animal Center (Guangzhou, China). The SD rats were intraperitoneally injected with either CDCA (20 mg/kg/day) or vehicle respectively for 2 weeks. Male C57BLKS/JGpt (BKS) mice and BKS-Leprem2Cd479/Gpt (db/db) mice (Blood glucose is more than 20 mmol/L) aged 5 weeks were purchased from GemPharmatech Co., Ltd. (Nanjing, China). FXR knockout mice (C57Bl/6) were kindly provided by Prof. Youfei Guan at Dalian University, China [34]. All the animal procedures were performed according to the principles of laboratory animal care and approved by the Shenzhen University Animal Care Committee. All rat or mouse pancreata were excised for immunostaining or were digested by collagenase P to collect islets. The samples in each group should be random allocated and the number should be greater than or eaqual to 3. No treatment other than necessary intervention is given.

### Cell lines

RPMI 1640 medium was used to culture INS-1 832/13 cells [35]. FXR knockdown or overexpressing INS-1-1 832/13 cells were generated as reported [36]. To overexpress COX6A2, INS-1 832/13 cells were transduced with pCDH-CMV-MCS-EF1-Puro-3xFlag (vector) and pCDH-CMV-MCS-EF1-Puro-3xFlag-COX6A2 (COX6A2) and then selected with puromycin (3 μg/ml) for 1 week. For knockdown of *Cox6a2* or Bax, INS-1 832/13 cells were transduced with either scramble or shCOX6A2 (RMM3981-201787879) or shBax (TRCN0000273037) plasmid and then selected with puromycin (3 μg/ml) for 1 week.

### In vivo delivery of *Cox6a2* knockout adeno-associated virus

The AAV9-*Cox6a2*-KO (adeno-associated virus) plasmid was generated by inserting a sgRNA (5’-TAAGGTCCTCAGTCGGAGCA-3’) that targets against *Cox6a2* gene (Rat) into pX601-AAV-CMV-SaCas9-3xHA-U6-sgRNA vector. The AAV9 was packaged and purchased from Vigene Biosciences Inc (China). To decrease the expression of COX6A2 in islets in vivo, GK rats were anesthetized with continuous isoflurane and then 50 μL of AAV9-*Cox6a2*-KO (2×10 12 virus particles) was injected through the common bile duct. The rats injected with 50 μL of scramble control AAV viral vectors were used as the control group.

### Intraperitoneal Glucose Tolerance Test (IPGTT) and Intraperitoneal Insulin Tolerance Test (IPITT)

Rats or mice were fasted overnight before the IPGTT, after which they were given intraperitoneal injections of glucose at a dosage of 2 g/kg body weight. The rats or mice were fasted 6 h before undergoing the IPITT and were then administered human insulin at a dosage of 0.75 U/kg body weight through intraperitoneal injection. Blood glucose levels were determined at 15, 30, 60, 90, and 120 min after glucose or human insulin injection. The blood glucose level was determined with a glucometer.

### Plasma insulin level detection

Blood samples were collected from rats or mice via jugular vein or supraorbital sinus, followed by centrifugation at 2000 g, 4 °C for 10 min. The supernatant was collected as plasma. The plasma insulin levels were determined using the ultrasensitive insulin Elisa kit (ALPCO).

### Western blotting analysis

The experiment method was performed as described [37]. The following antibodies were used: FXR (Biorbyt, orb156973), COX6A2 (Proteintech, 11421-1-AP), Cytochrome C (Cell signaling, #11940), Insulin (Cell signaling, #8138S), COX IV (Cell signaling, #4850), cleaved-caspase3 (Cell Signaling, #9661), Bax (Proteintech, 60267-1-Ig), VDAC1 (Proteintech, 55259-1-AP), Flag (Proteintech, 80010-1-RR), GAPDH (Cell Signaling, #5174), Tubulin (Santa Cruz, sc-8035), Bcl-2 (Santa Cruz, sc- 7382), and anti-β-actin (Sigma, A5441). The densities of the immunoblot bands were determined by Gel-Pro Analyzer 4.0 software.

### RNA extraction and Real-time PCR

Total RNA was extracted from INS-1 832/13 cells or rat/mouse islets using Trizol reagent (Invitrogen). Real-time PCR was performed with SYBR Green master mix (Promega) on the ABI QuantStudio 5 real-time PCR System. The primer sequences are listed in Supplementary Table 1. The relative expression level of the target gene mRNA was normalized to β-actin or GAPDH.

### Chromatin Immunoprecipitation (ChIP) assay

ChIP assays were carried out with a ChIP assay kit (Millipore) according to the manufacturer’s instructions. Soluble chromatin was extracted from INS-1 832/13 cells transduced with sh-FXR, and then immunoprecipitated with antibodies (2 μg) against p300 (Santa Cruz, sc-48343) or acetylated H3K27 (Abcam, ab4729) respectively. Thereafter, qPCR was used to purify and quantify the DNA fragments using the primers listed in Supplementary Table 2.

### Co-immunoprecipitation (Co-IP)

IP lysis buffer (50 mM Tris-Cl, 1% NP 40, 300 mM NaCl, 1 mM DTT, 2.5% Glycerol, pH 7.6) supplemented with 1 mM PI (protease inhibitor) and PMSF was used to lyse the INS-1 832/13 cells. 1 mg cell lysate buffer was incubated with 3 μg of VDAC1 (Santa Cruz, sc-390996), COX6A2 (Proteintech, 11421-1-AP), Flag (Proteintech, 80010-1-RR), Bax (Santa Cruz, sc-20067) or IgG (Abcam, ab27478) antibody at 4 °C overnight, followed by immunoprecipitation with 60 μl protein A agarose beads (Millipore, #16-157) for at least 3 h at 4 °C the second day. The complexes were then washed with low salt wash buffer (Millipore, #20-154), high salt wash buffer (Millipore, #20-155), LiCl wash buffer (Millipore, #20-156), and TE buffer (Millipore, #20-157), sequentially. Immunoprecipitated supernatants were separated using SDS-PAGE, followed by immunoblotting with VDAC1/Flag/Bax antibody and analysis using the Tanon5200 image system.

### Immunofluorescence

For the detection of co-localization of Insulin and cleaved-caspase3/mitochondria and Bax, the pancreatic sections of rats or mice were prepared and INS-1 832/13 cells were plated on Poly-L-lysine-coated coverslips. The sections and cells were fixed with 4% paraformaldehyde and permeabilized with 0.3% Triton X-100. After washing with PBS, the sections and cells were incubated with rabbit anti-cleaved-caspase3 (Cell Signaling, 1:100) and mouse anti-Insulin (Abcam, 1:100) primary antibodies overnight at 4 °C followed by incubation with Alexa-Fluor546-labeled goat anti-rabbit (Invitrogen, 1:1000) and Alexa-Fluor488-labeled goat anti-mouse (Invitrogen, 1:1000) secondary antibodies for one hour. The sections and cells were then incubated with DAPI dye for 10 min at room temperature. Finally, the ZEISS 510 LSM confocal laser-scanning microscope was used to observe the images.

### Mitochondrial transmembrane potential (ΔΨm) measurement

The JC-1 Mitochondrial Membrane Potential Assay Kit (Beyotime Biotechnology, China) was used to analyze the ΔΨm of INS-1 832/13 cells. The cells were placed in 6-well plates and then incubated with JC-1 dye (1:200) for 20 min at 37 °C. The cell-associated fluorescence was determined by Axio Observer 3 (ZEISS, Germany) fluorescent microscope.

### Flow cytometry

Cell apoptosis was quantified by Annexin V-FITC/PI (BD Biosciences). After washing with PBS twice, the cells were stained with Annexin V-FITC/PI according to the manufacturer’s instructions. Subsequently, the cells were analyzed via flow cytometry (FACScan, BD Biosciences) and apoptotic fractions were obtained.

### Statistical analyses

Statistical analysis was performed by SPSS20.0 software. An independent t-test (for two groups) was used to measure the statistical differences. *P* < 0.05 was considered statistically significant.

## Supplementary information

Supplementary Figure S1

Supplementary Figure S2

Supplementary Figure S3

Supplementary Figure S4

Supplementary Figure S5

Supplementary Figure S6

Supplementary Figure S7

Supplementary Figure S8

Supplementary Figure S9

Supplemental Material 1

Supplemental Material 2

Supplemental Material 3

Supplementary Table 1

Supplementary Table 2

## Data Availability

The raw and processed RNA-seq data of FXR knockout mice have been uploaded to GEO database under accession number GSE279682. And all the datasets generated and/or analyzed during the current study are available from the corresponding author upon reasonable request.
