# Supplementary figures and images for: FXR-regulated COX6A2 triggers mitochondrial apoptosis of pancreatic β-cell in type 2 diabetes
Source: Cell Death Dis. 2024 Dec 20;15(12):920. doi: 10.1038/s41419-024-07302-4 (PMC11659401; doi:10.1038/s41419-024-07302-4)

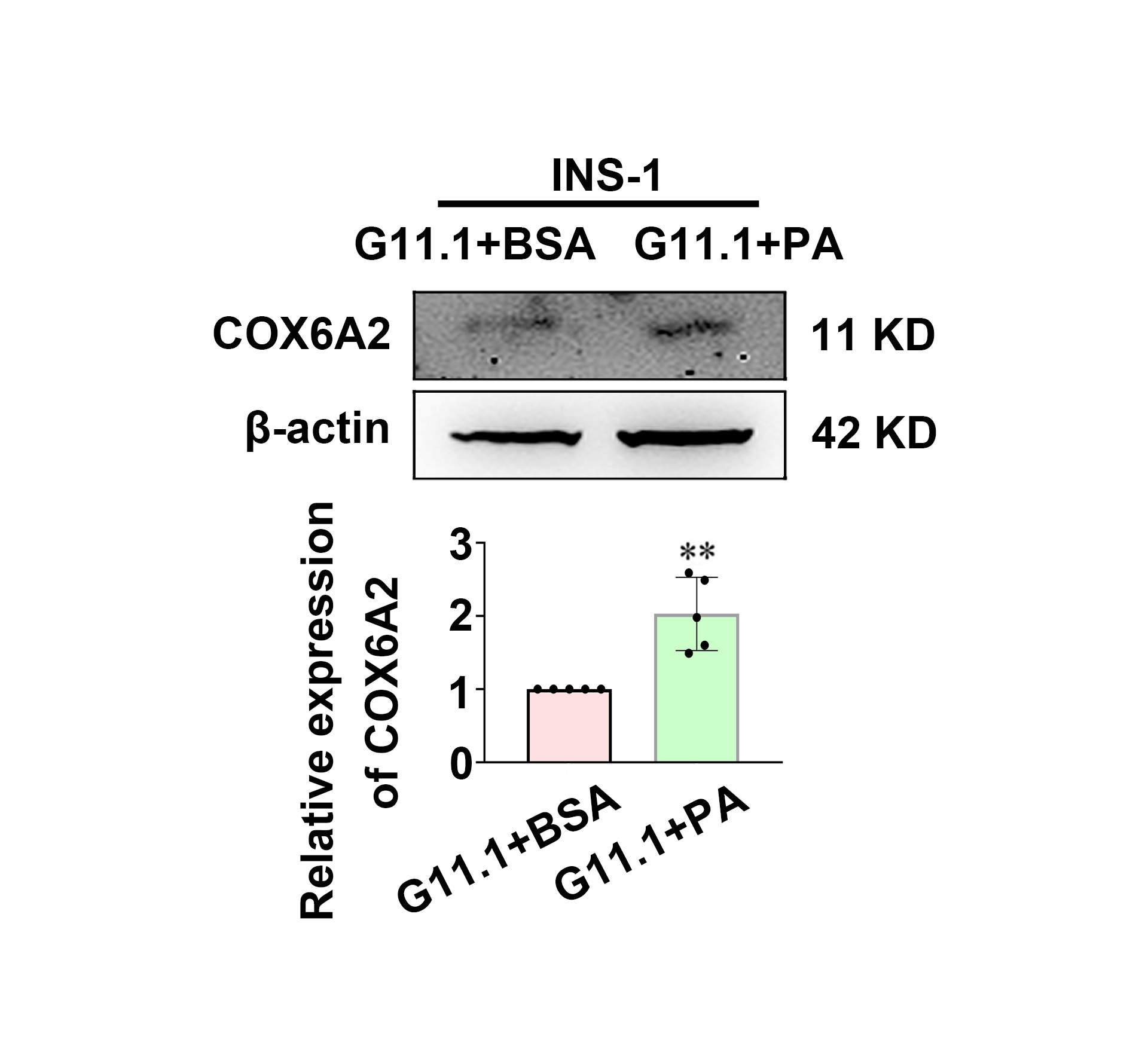

Supplement: Supplementary file 1 — Supplementary Figure S1 [file 41419_2024_7302_MOESM1_ESM.tif]

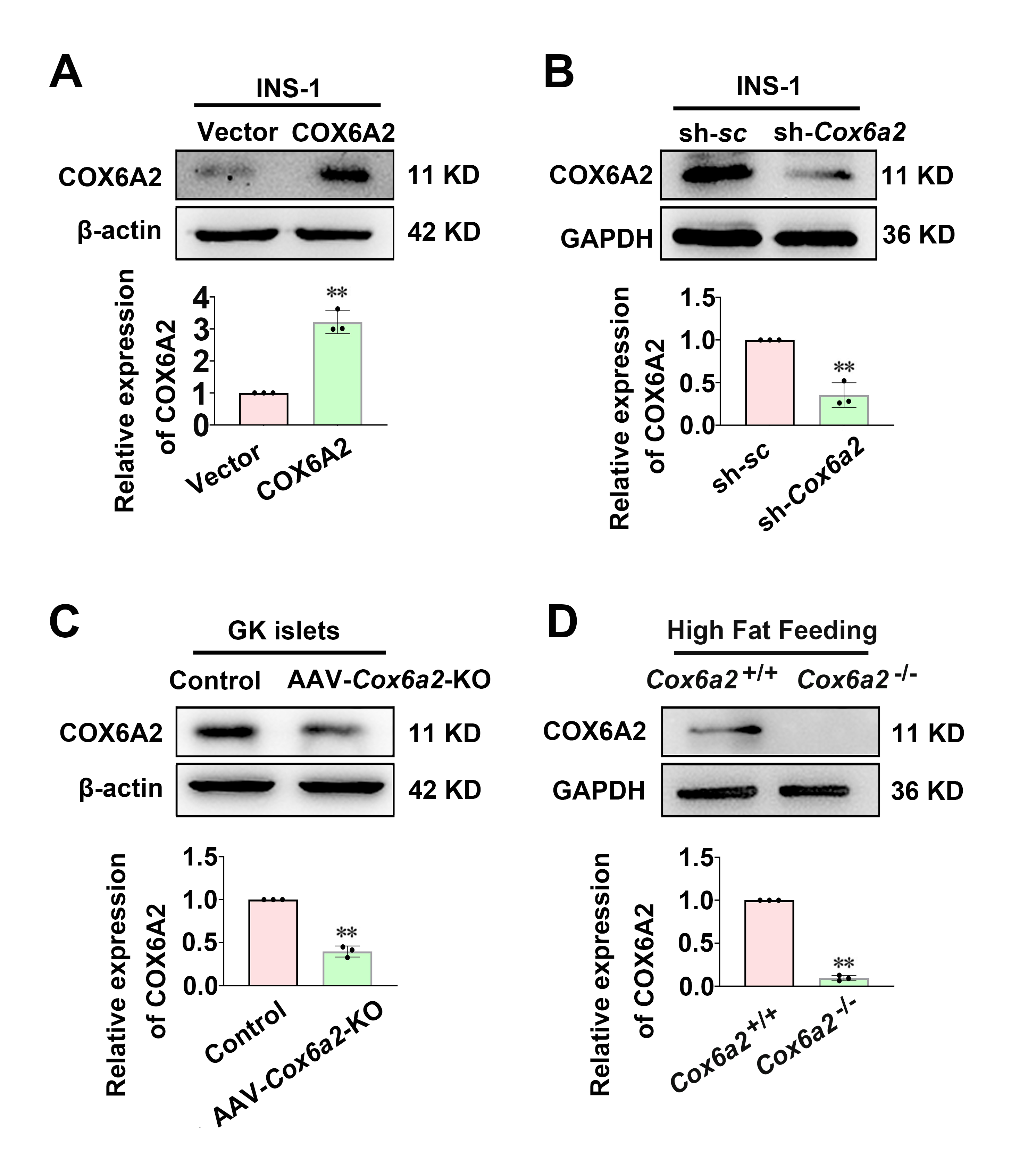

Supplement: Supplementary file 2 — Supplementary Figure S2 [file 41419_2024_7302_MOESM2_ESM.tif]

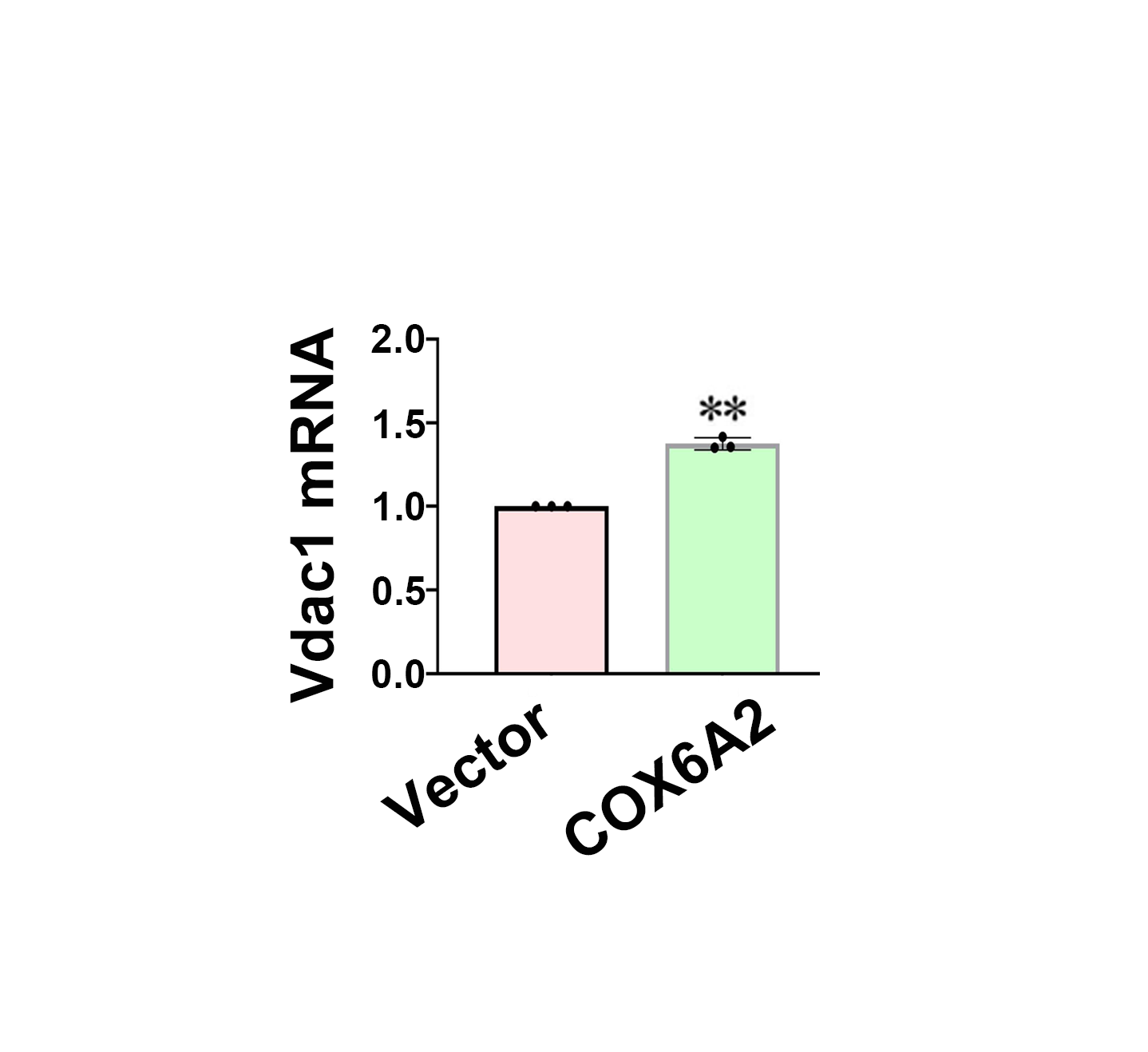

Supplement: Supplementary file 3 — Supplementary Figure S3 [file 41419_2024_7302_MOESM3_ESM.tif]

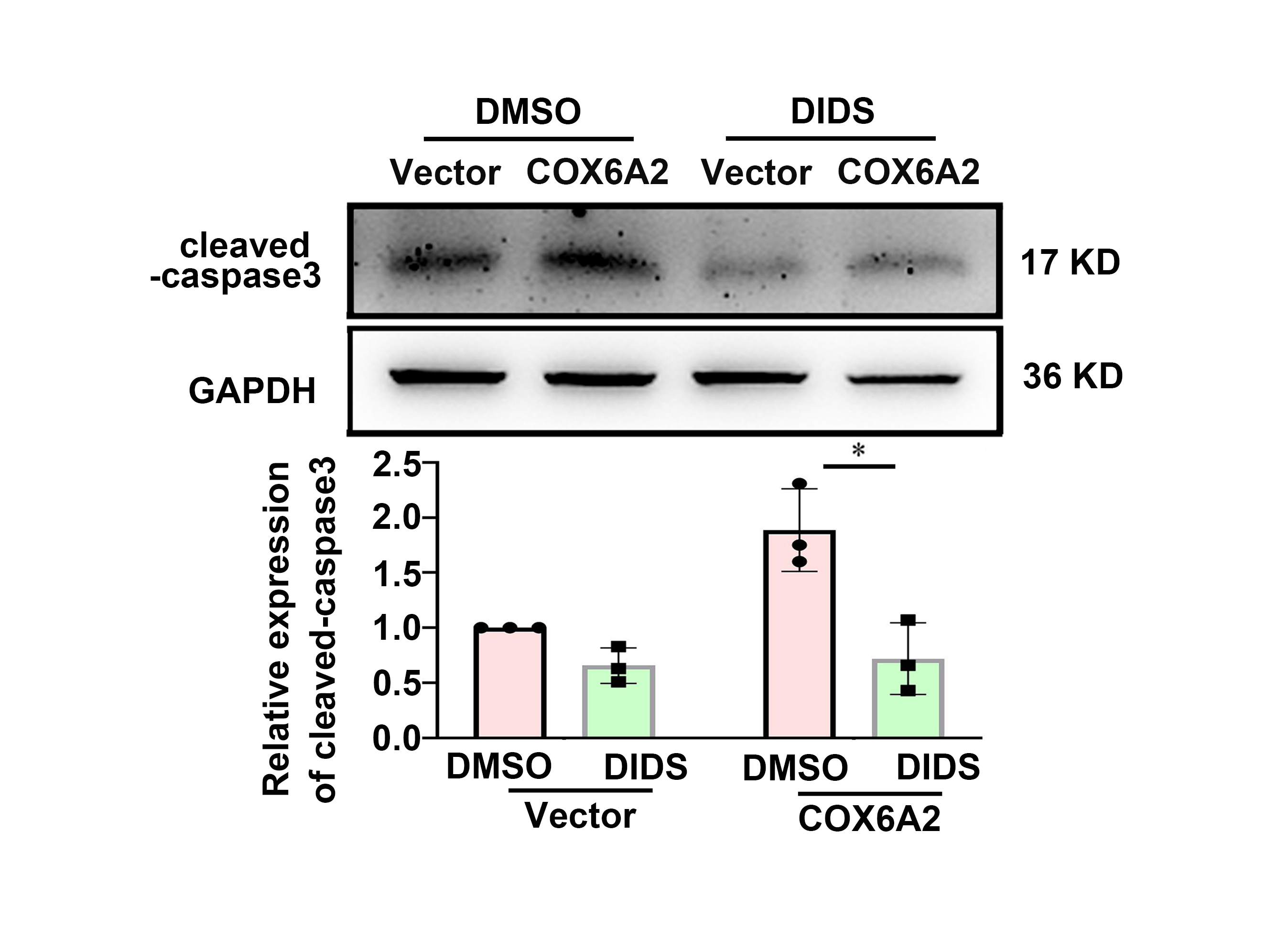

Supplement: Supplementary file 4 — Supplementary Figure S4 [file 41419_2024_7302_MOESM4_ESM.tif]

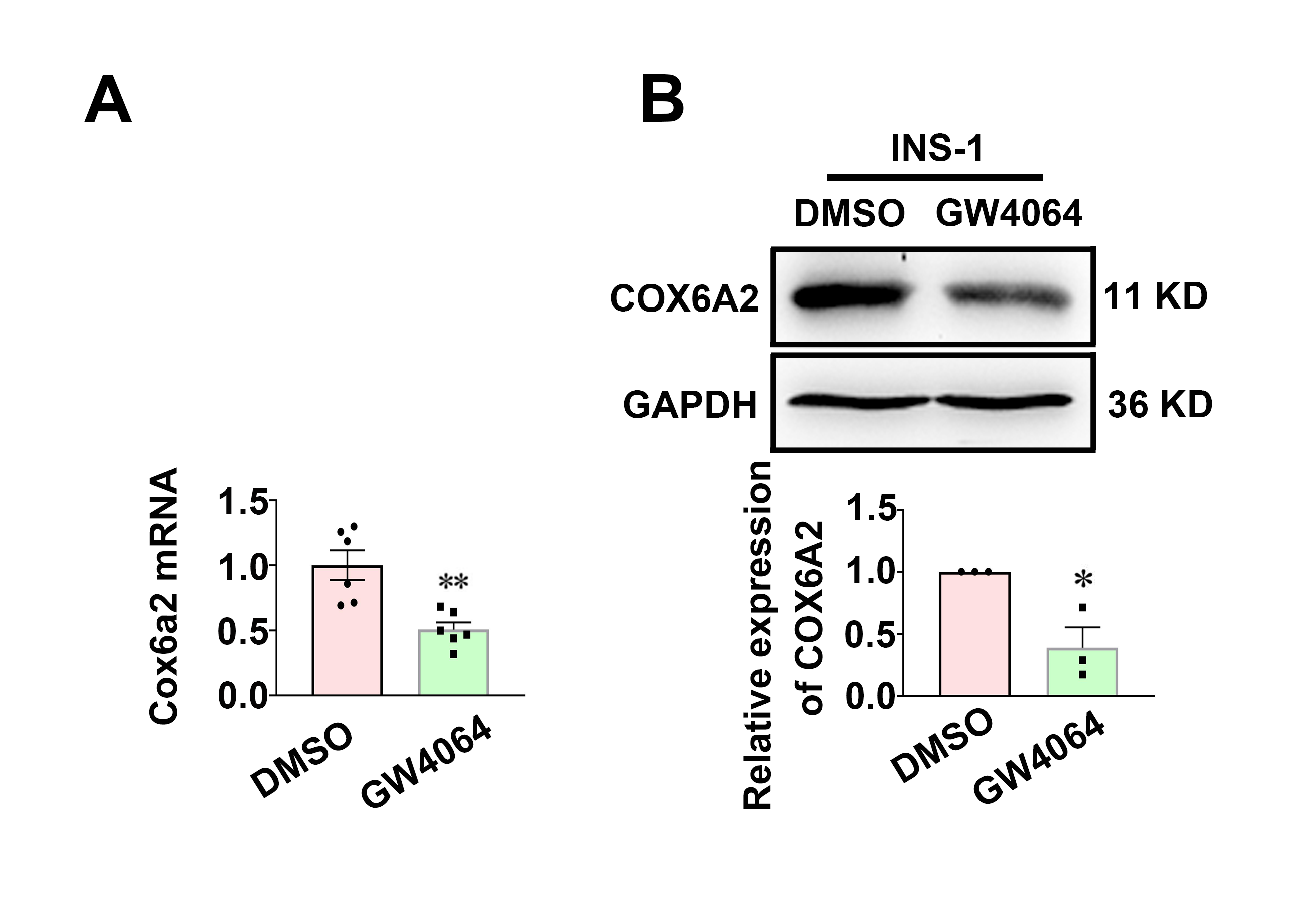

Supplement: Supplementary file 5 — Supplementary Figure S5 [file 41419_2024_7302_MOESM5_ESM.tif]

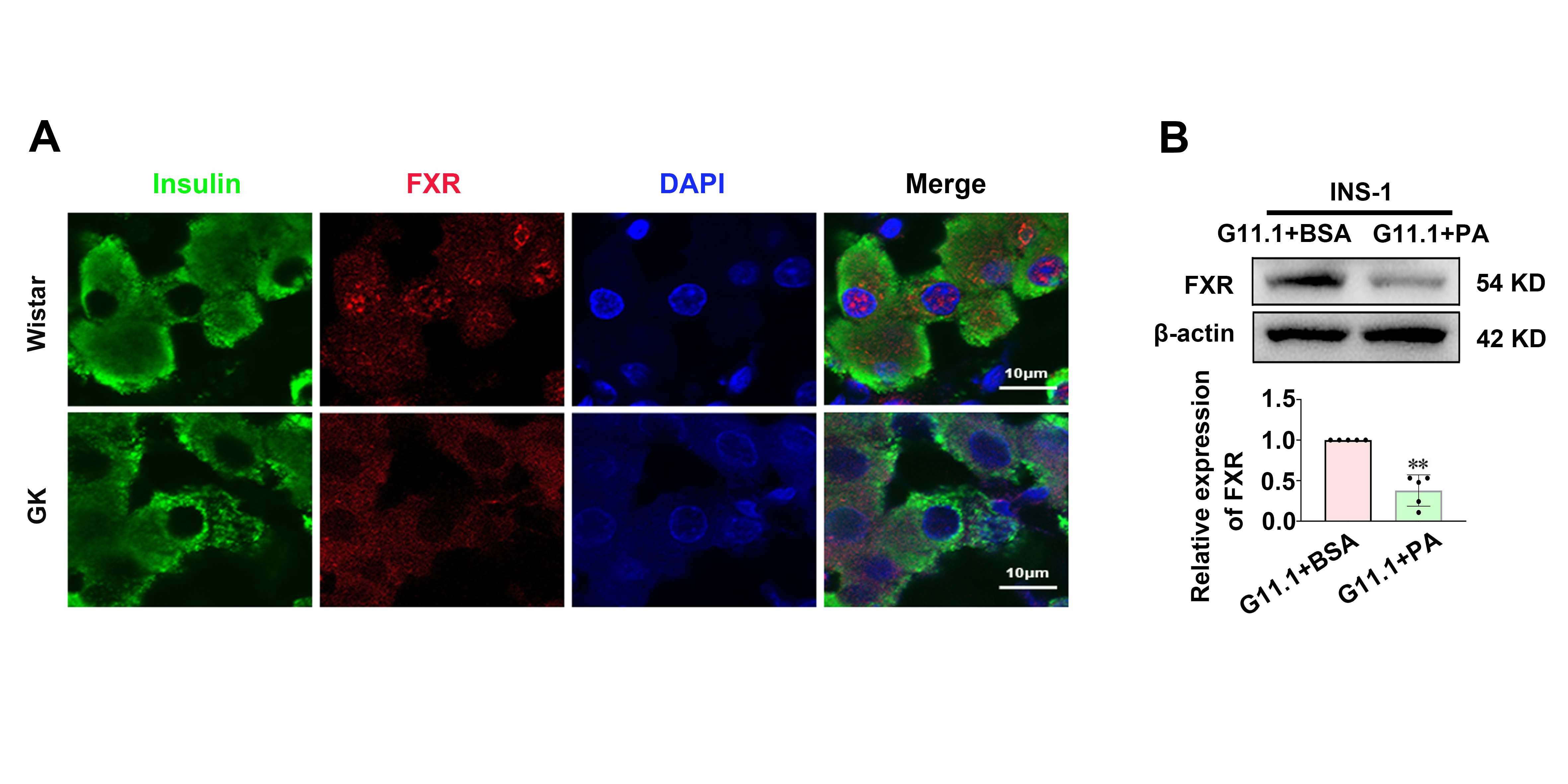

Supplement: Supplementary file 6 — Supplementary Figure S6 [file 41419_2024_7302_MOESM6_ESM.tif]

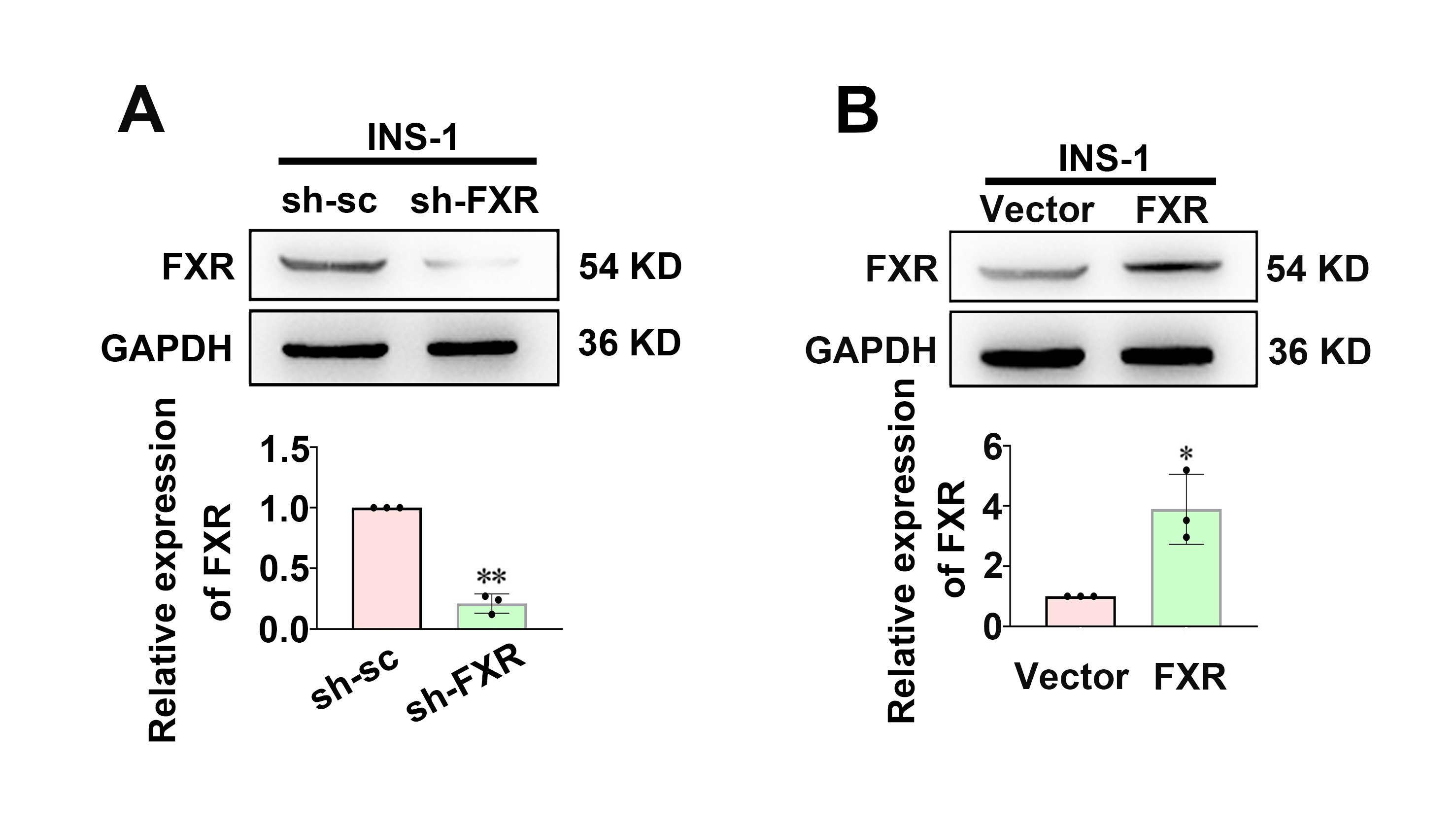

Supplement: Supplementary file 7 — Supplementary Figure S7 [file 41419_2024_7302_MOESM7_ESM.tif]

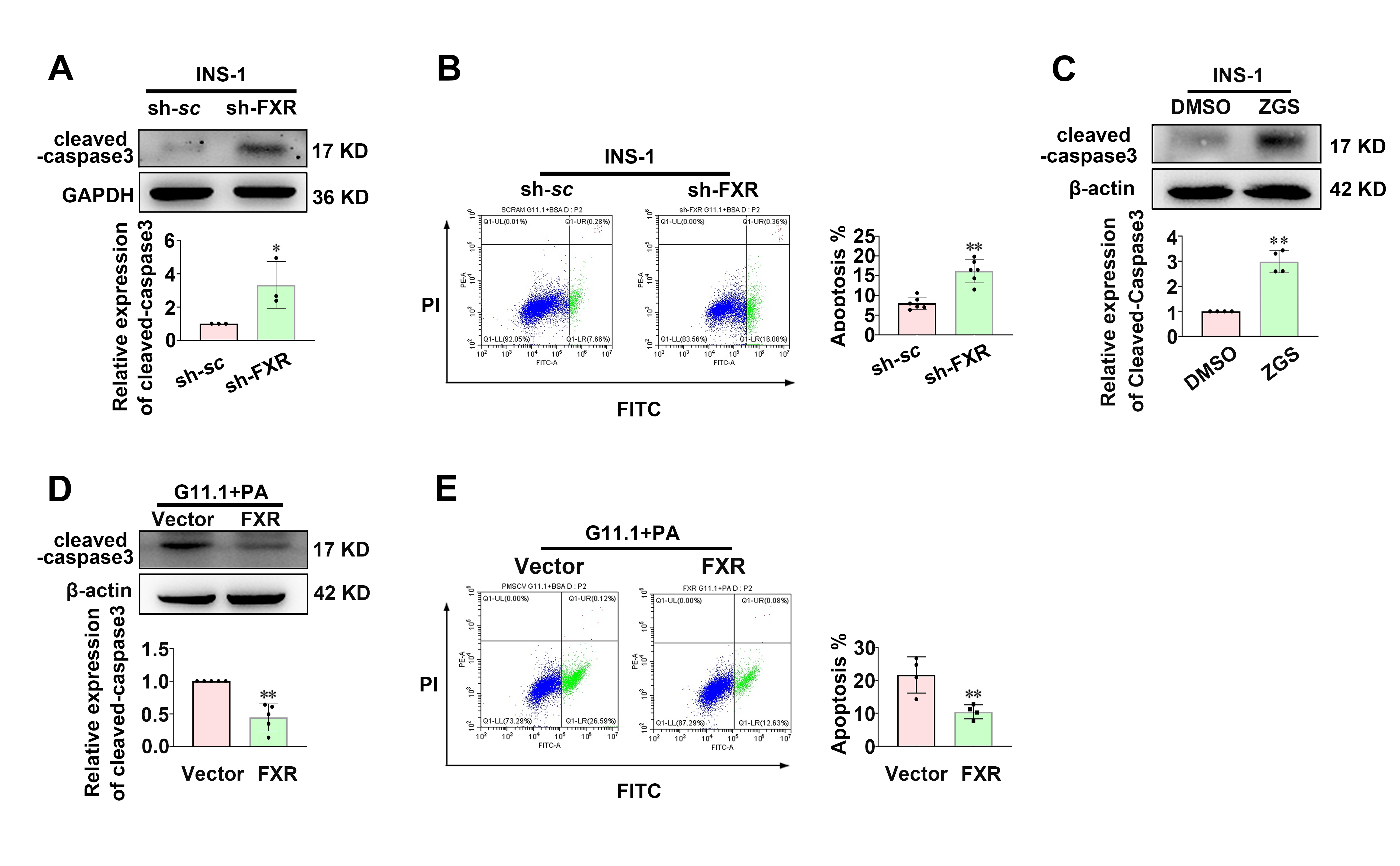

Supplement: Supplementary file 8 — Supplementary Figure S8 [file 41419_2024_7302_MOESM8_ESM.tif]

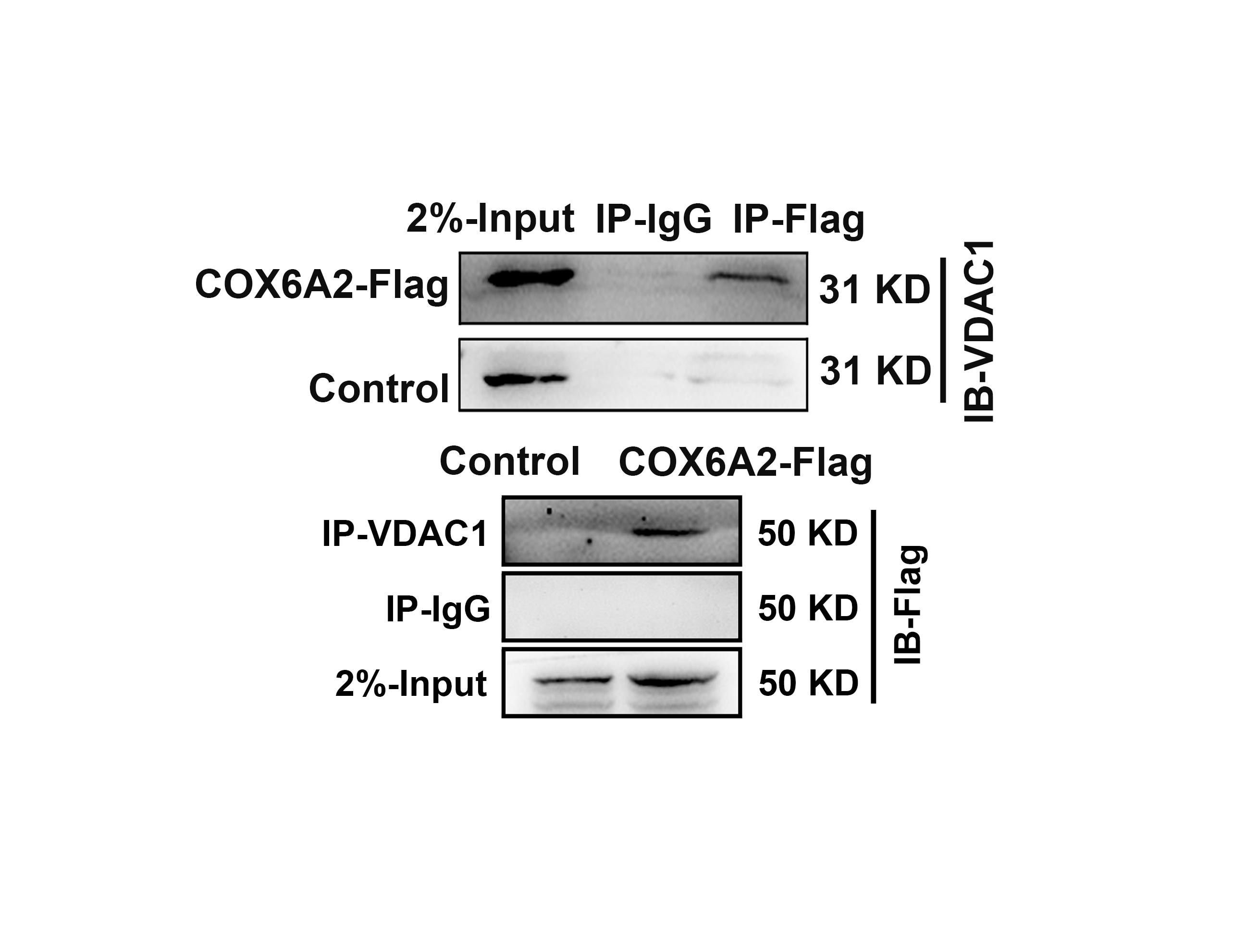

Supplement: Supplementary file 9 — Supplementary Figure S9 [file 41419_2024_7302_MOESM9_ESM.tif]

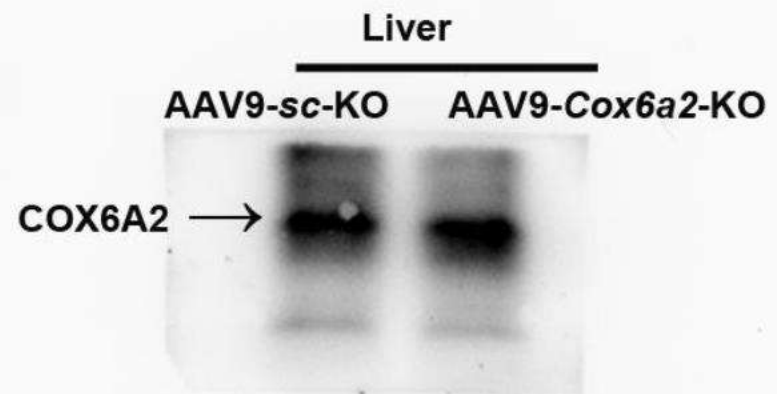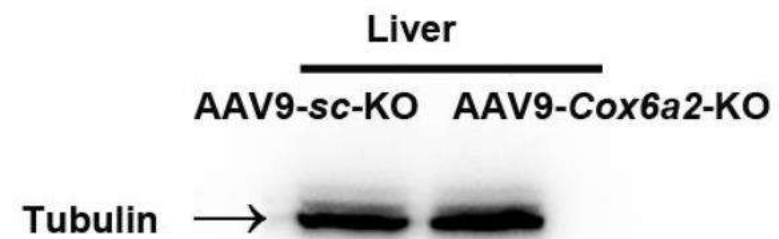

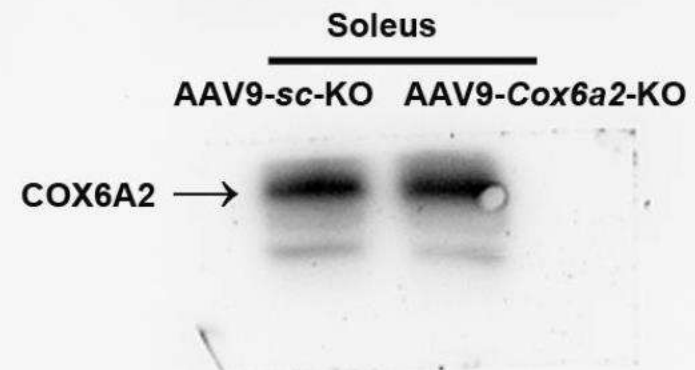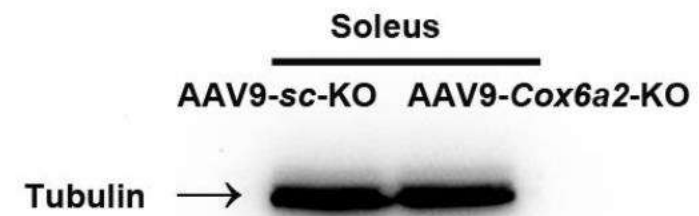

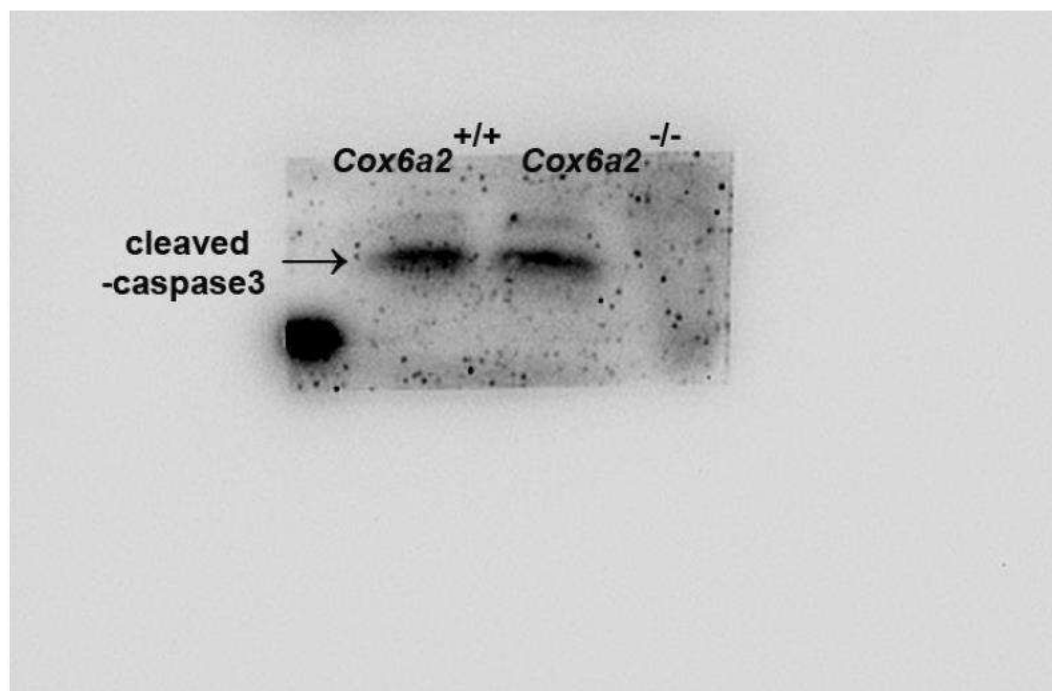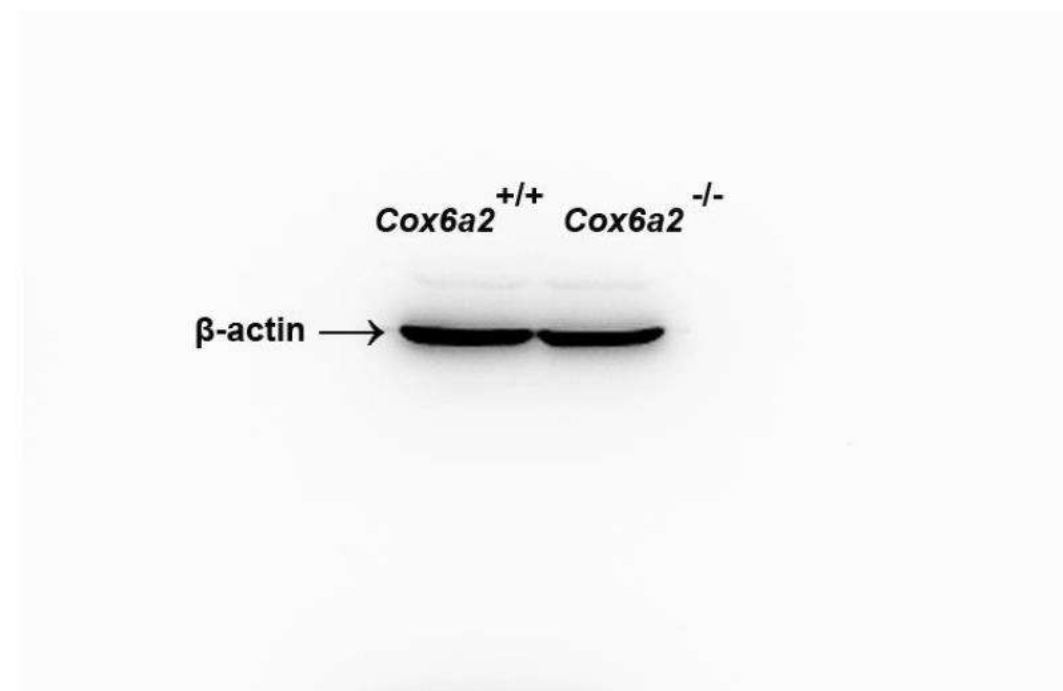

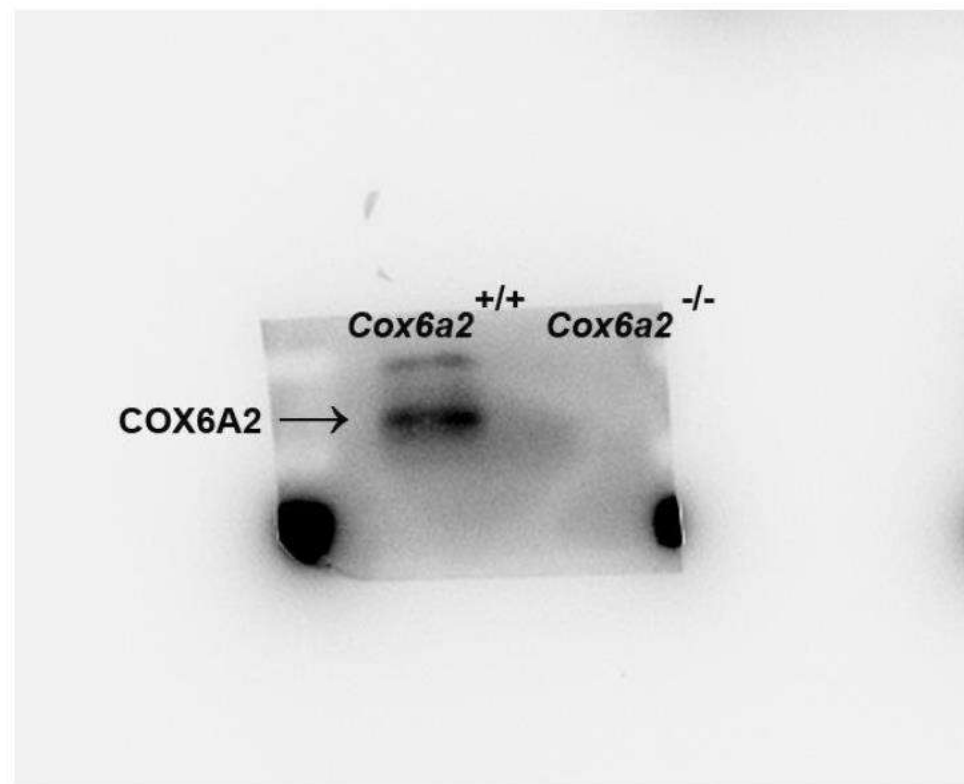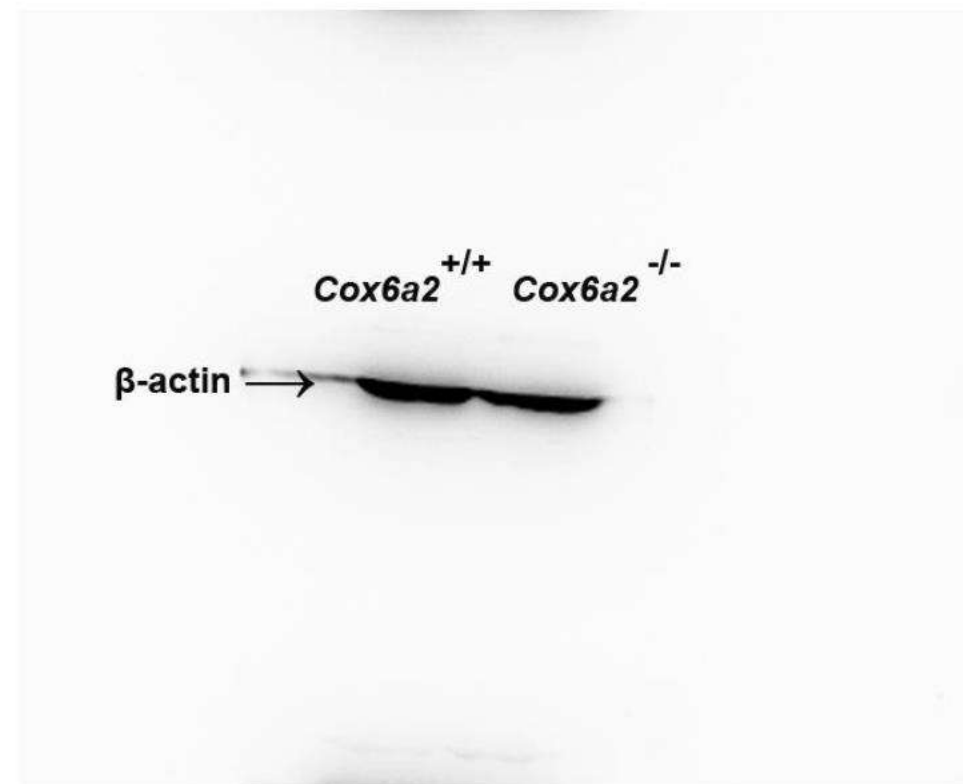

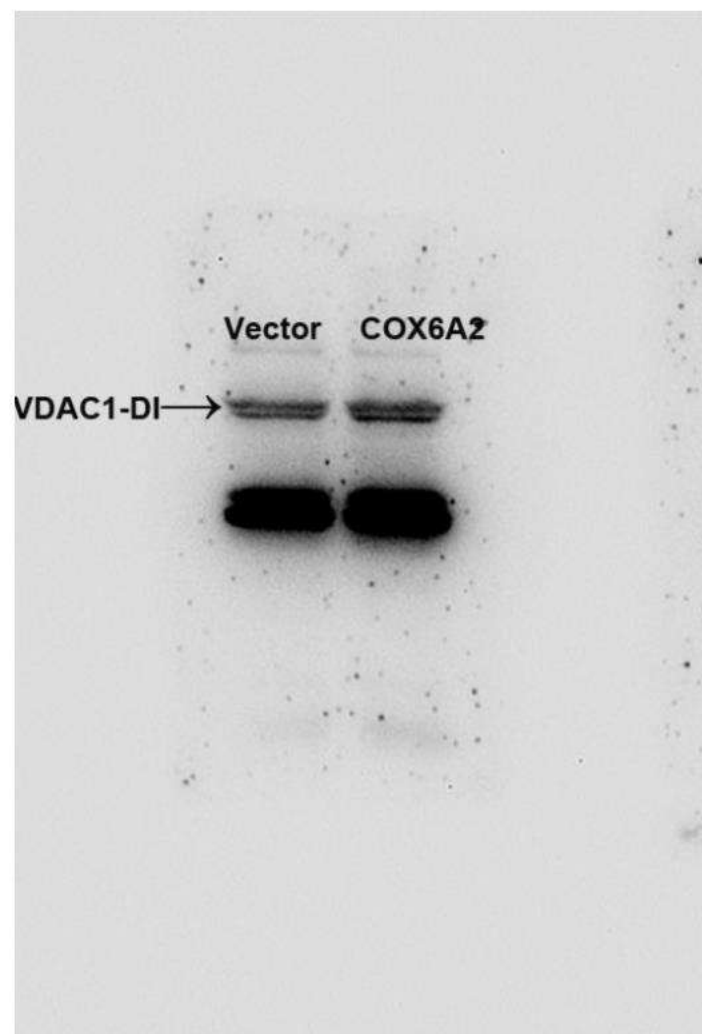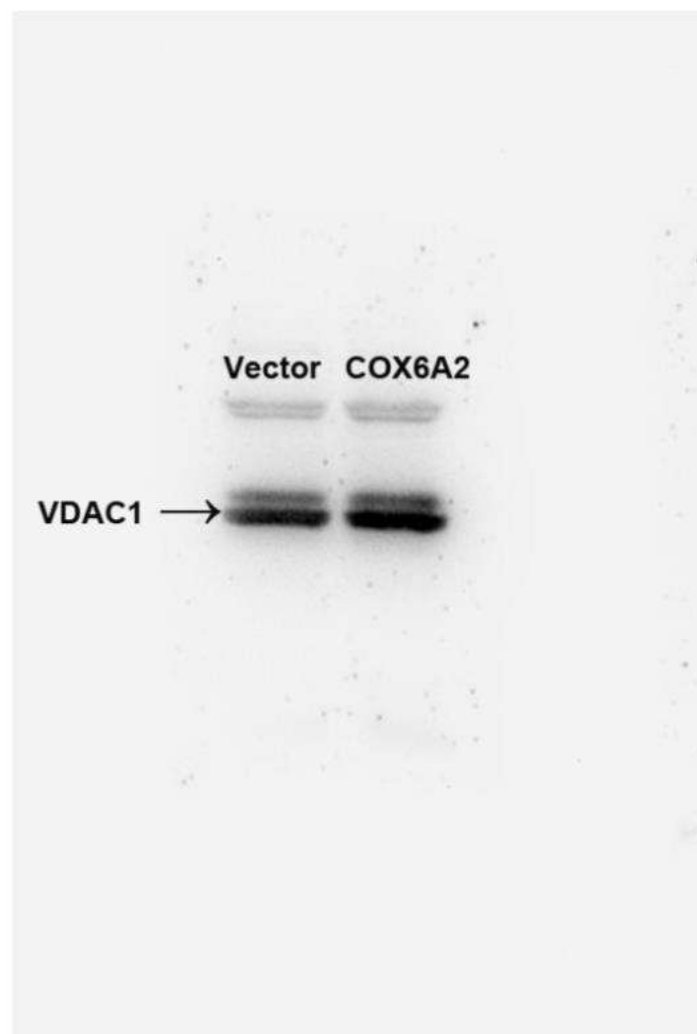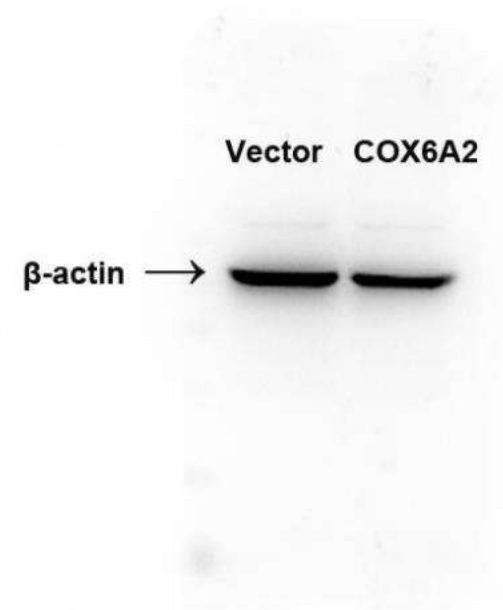

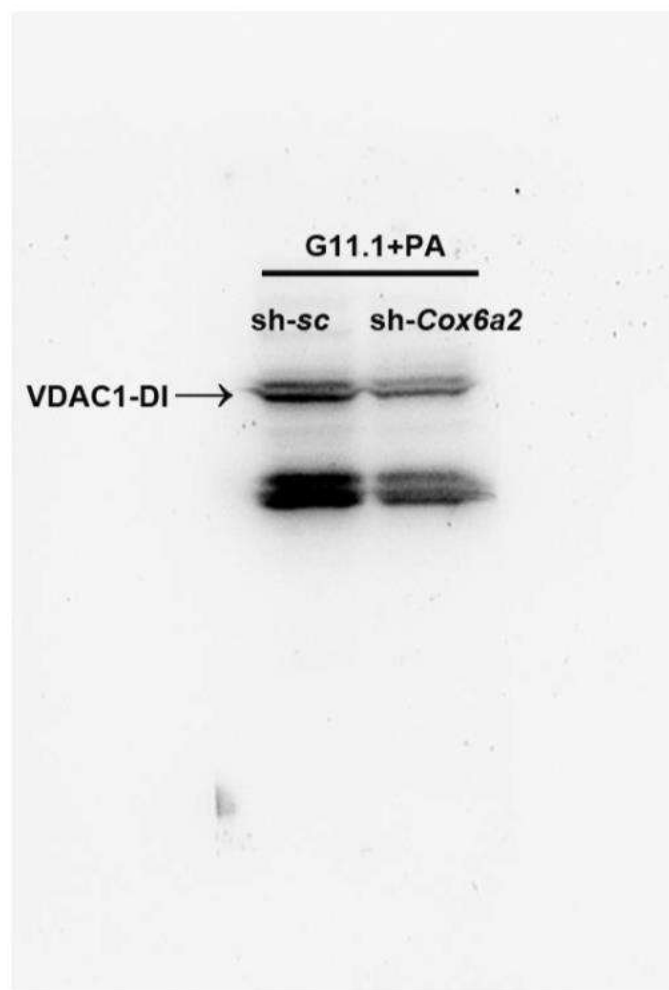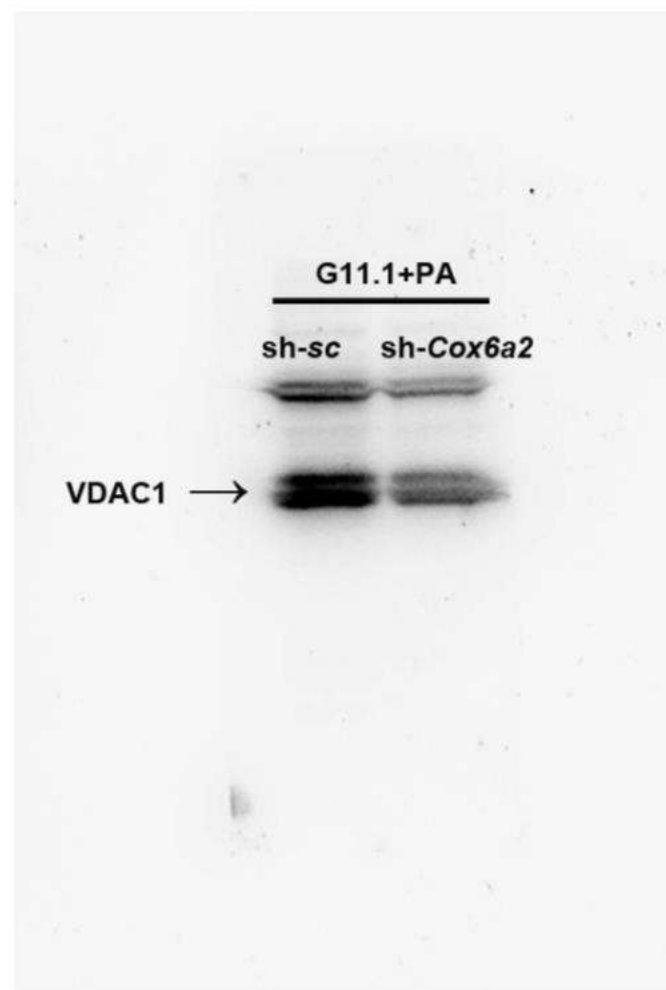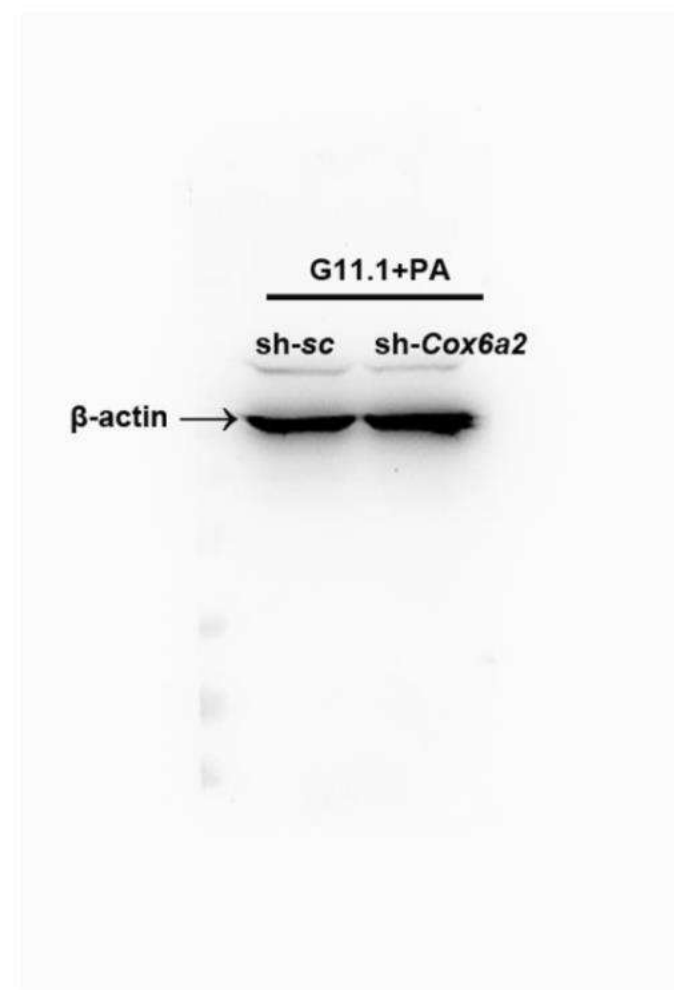

COX6A2 →

2%-Input

IP-VDAC1

IP-IgG

VDAC1 →

2%-Input

IP-COX6A2

IP-IgG

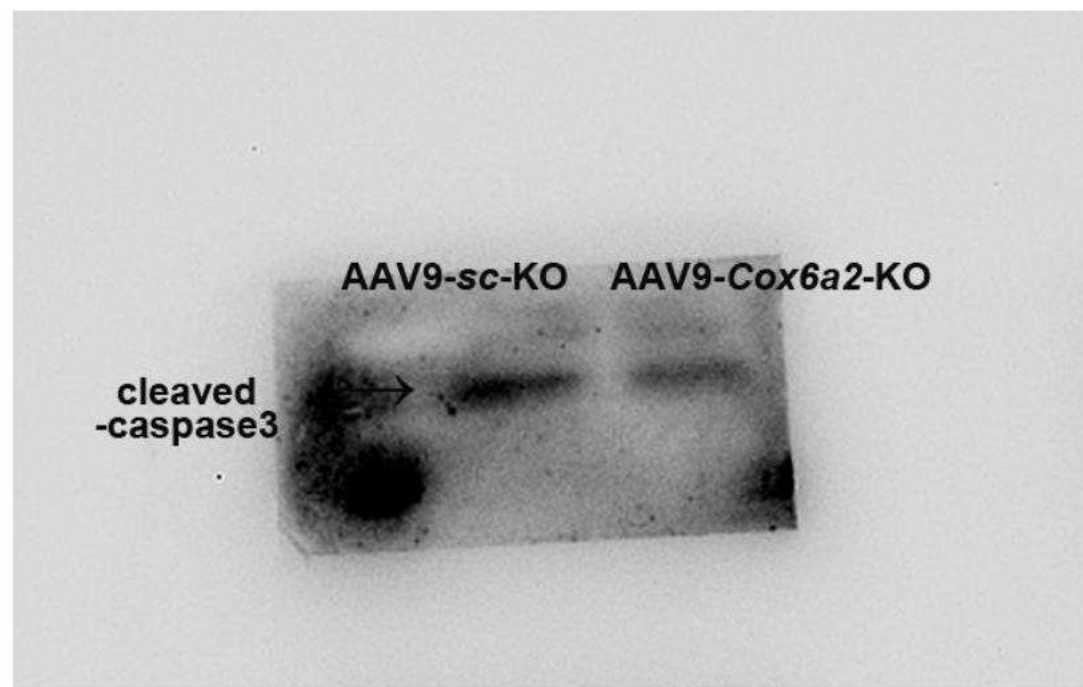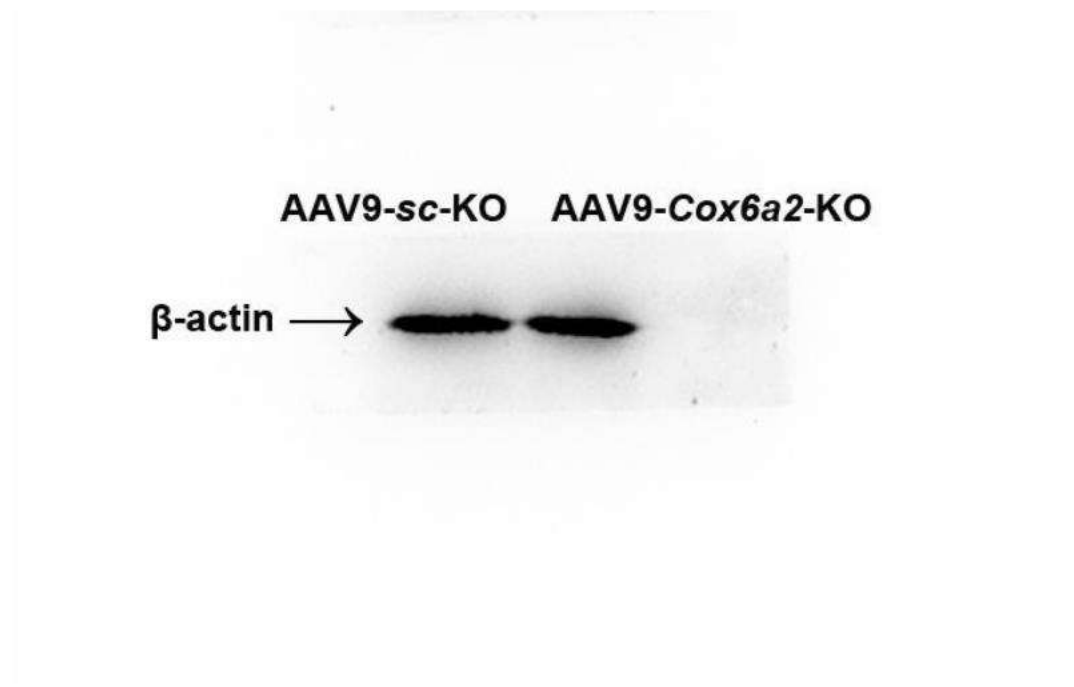

Supplement: Supplementary file 12 — Supplemental Material 3 [file 41419_2024_7302_MOESM12_ESM.pdf]
