# Supplementary material for: FXR-regulated COX6A2 triggers mitochondrial apoptosis of pancreatic β-cell in type 2 diabetes
Source: Cell Death Dis. 2024 Dec 20;15(12):920. doi: 10.1038/s41419-024-07302-4 (PMC11659401; doi:10.1038/s41419-024-07302-4)
Supplement: Supplementary file 10 — Supplemental Material 1 [file 41419_2024_7302_MOESM10_ESM.docx]

**Supplementary materials**

**Supplementary Figure legends**

**Supplementary Figure S1.** Palmitic acid induces COX6A2 protein expression in INS-1 832/13 cells

COX6A2 protein was determined in INS-1 832/13 cells by treatment with 10% BSA or

0.4 mM palmitic acid (PA) for 48 h, respectively. Bars represent means ±SEM, n=5. **, p<0.01 (t-test).

**Supplementary Figure S2.** The protein expression of COX6A2 in β-cells under different conditions

(**A**) COX6A2 protein was examined in vector and COX6A2 overexpression INS-1 832/13 cells. Bars represent means ±SEM, n=3. **, p<0.01 (t-test). (**B**) COX6A2 protein was determined in the scramble and sh-*Cox6a2* INS-1 832/13 cells. Bars represent means±SEM, n=3. **, p<0.01 (t-test). (**C**) The protein level of COX6A2 was examined in islets from GK-AAV9-vector and GK-AAV9-*Cox6a2*-KO rats. Bars represent means ±SEM, n=3. **, p<0.01 (t-test). (**D**) COX6A2 protein expression was examined in islets from *Cox6a2*^+/+^ and *Cox6a2*^-/-^ mice fed with HFD. Bars represent means ±SEM, n=3. **, p<0.01 (t-test).

**Supplementary Figure S3.** *Vdac1* mRNA is increased in COX6A2 overexpression INS-1 832/13 cells

*Vdac1* mRNA was determined in vector and COX6A2 overexpression INS-1 832/13 cells, Bars represent means ±SEM, n=3. **, p<0.01 (t-test).

**Supplementary Figure S4.** Inhibition of VDAC1 attenuates the increase of cleaved-caspase3 level induced by COX6A2 overexpression

The protein level of cleaved-caspase3 was examined in vector and COX6A2 overexpression INS-1 832/13 cells treated with DMSO or 200 μM DIDS for 2 h respectively. Bars represent means ±SEM, n=3. *, p<0.05 (t-test).

**Supplementary Figure S5.** Activation of FXR inhibits COX6A2 expression in INS-1

832/13 cells

(**A, B**) COX6A2 mRNA (**A**) and protein (**B**) were determined in INS-1 832/13 cells by

treatment with DMSO or 5 μM GW4064 for 24 h, respectively. Bars represent means ±SEM, n=6 (**A**) or 3 (**B**). *, p<0.05; **, p<0.01 (t-test).

**Supplementary Figure S6.** FXR expression is decreased in β-cells under diabetic conditions

(**A**) Representative immunostaining images for FXR (red), Insulin (green), DAPI (blue), and merge of the three in islets from Wistar and GK Rat; Bars represent 10 μm. (**B**) FXR protein expression was determined in INS-1 832/13 cells by treatment with 10% BSA or 0.4 mM PA for 48 h, respectively. Bars represent means ±SEM, n=5. **, p<0.01 (t-test).

**Supplementary Figure S7.** FXR protein levels were determined in FXR knockdown or overexpression INS-1 832/13 cells

(**A**) FXR protein levels were determined in scramble control and sh-FXR INS-1 832/13

cells. Bars represent means ±SEM, n=3. **, p<0.01 (t-test). (**B**) The protein level of FXR was determined in vector and FXR overexpression INS-1 832/13 cells. Bars represent means ±SEM, n=3. *, p<0.05 (t-test).

**Supplementary Figure S8.** FXR inhibits cell apoptosis in INS-1 832/13 cells

(**A**) The protein level of cleaved-caspase3 was examined in scramble control and sh- FXR INS-1 832/13 cells. Bars represent means ±SEM, n=3. *, p<0.05 (t-test). (**B**) A flow cytometer was used to determine the apoptosis in scramble control and sh-FXR INS-1 832/13 cells. n=6. **, p<0.01 (t-test). (**C**) The level of cleaved-caspase3 was examined in INS-1 832/13 cells treated with DMSO or 20 μM ZGS for 48 h, respectively. Bars represent means ±SEM, n=4. **, p<0.01 (t-test). (**D**)The cleaved-caspase3 protein was determined in vector and FXR overexpression INS-1 832/13 cells by treatment with 0.4 mM PA for 48 h. Bars represent means ±SEM, n=5. **, p<0.01 (t-test). (**E**) A flow cytometer was used to determine the apoptosis in vector and FXR overexpression INS-1 832/13 cells exposure to 0.4 mM PA for 48 h. n=4. **, p<0.01 (t-test).

**Supplementary Figure S9.** COX6A2 interacted with VDAC1

Anti-flag and anti-VDAC1 antibodies were used to carry out a protein pull-down assay of VDAC1 and COX6A2 in COX6A2 overexpressing INS-1 832/13 cells which were tagged with Flag.
