# Supplementary material for: FXR-regulated COX6A2 triggers mitochondrial apoptosis of pancreatic β-cell in type 2 diabetes
Source: Cell Death Dis. 2024 Dec 20;15(12):920. doi: 10.1038/s41419-024-07302-4 (PMC11659401; doi:10.1038/s41419-024-07302-4)

Fig. 1A

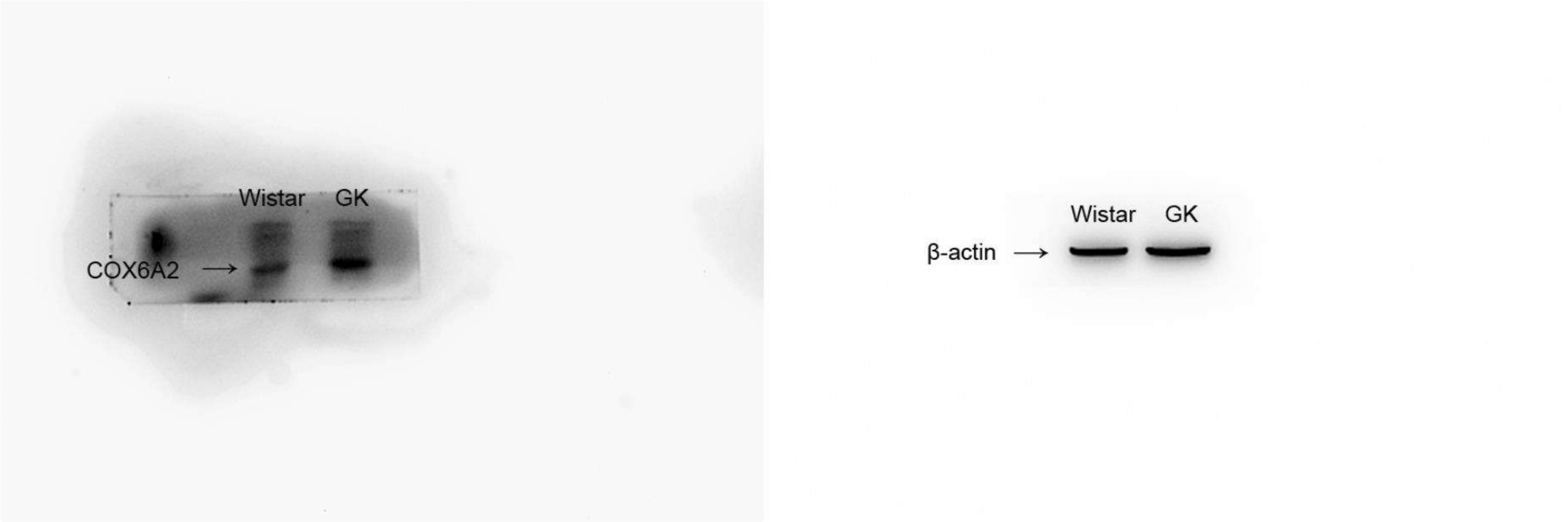

Fig. 1B

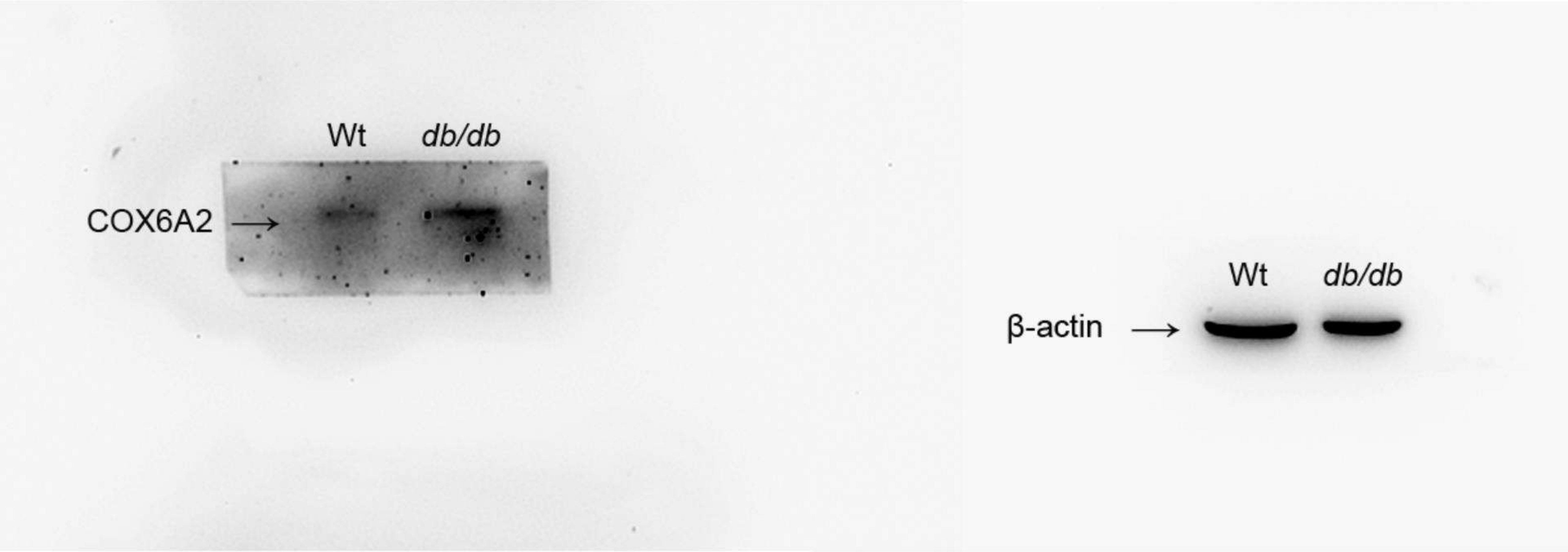

Fig. 1C

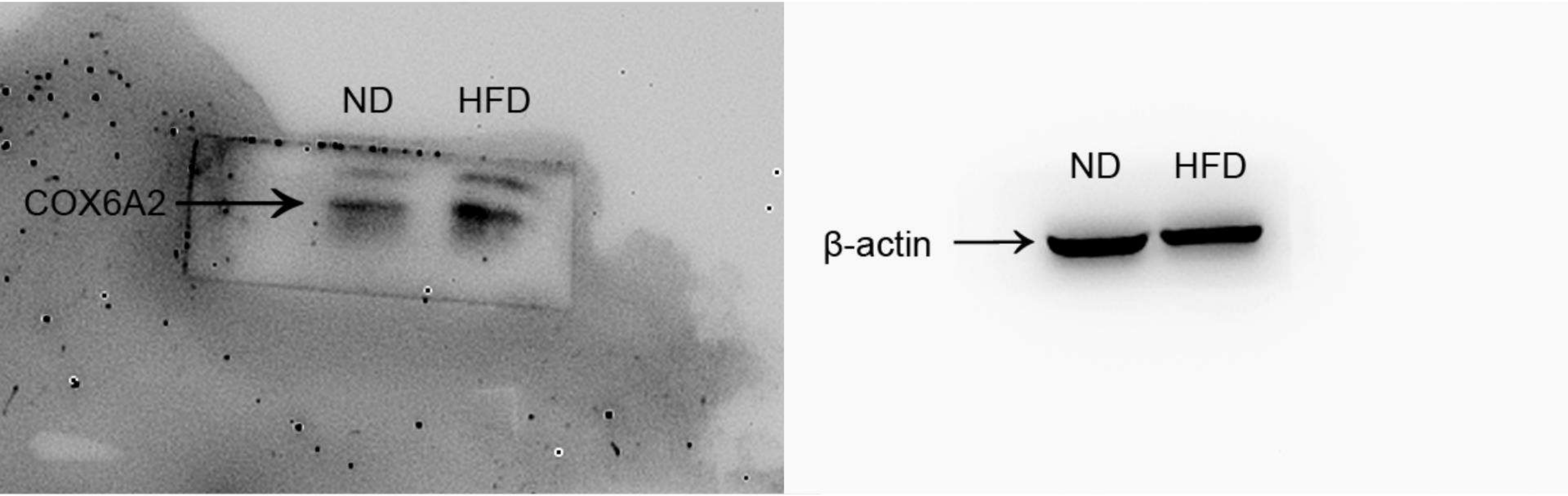

Fig. 1D

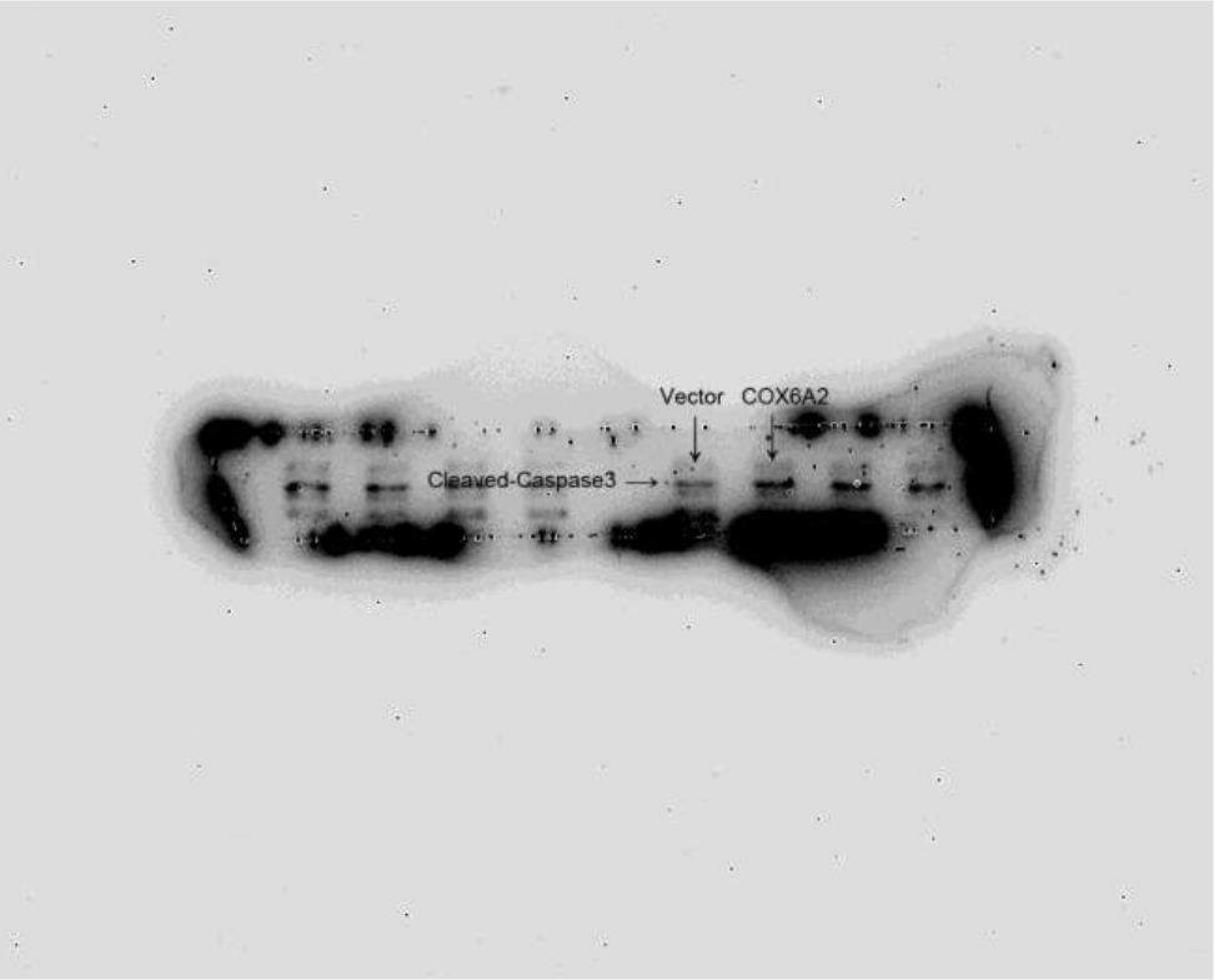

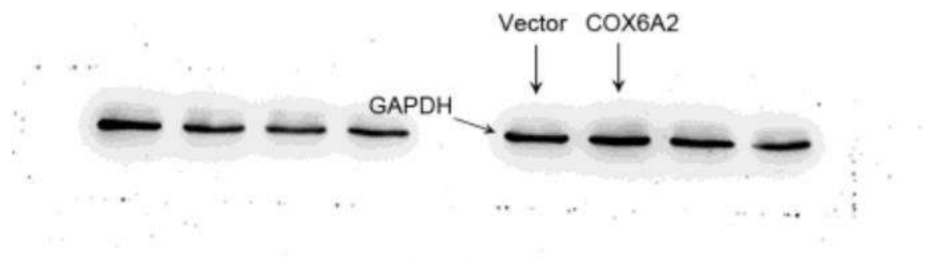

Fig. 1F

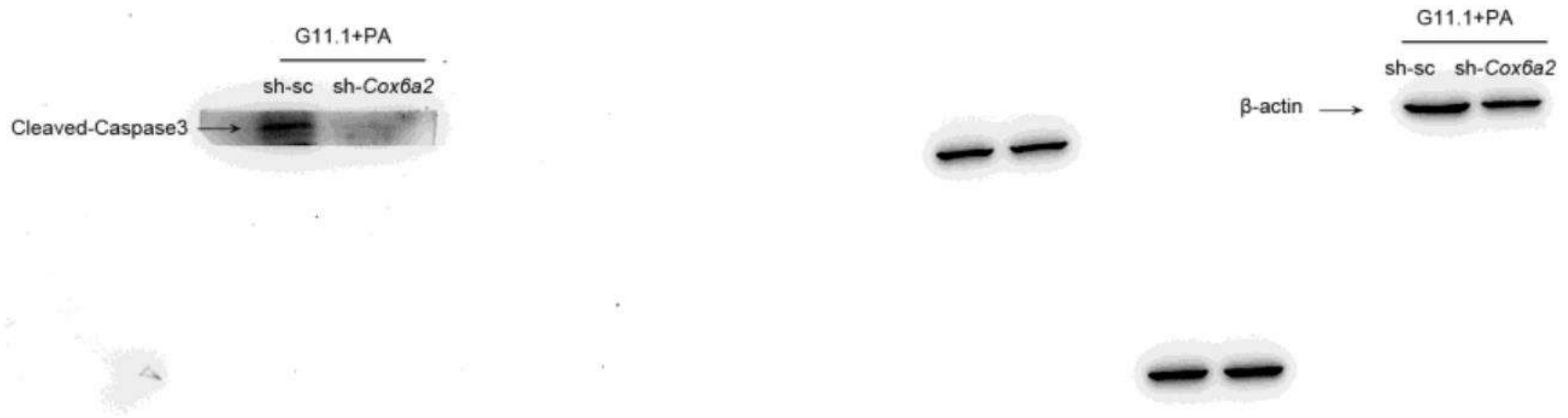

G11.1+PA
sh-sc
sh-Cox6a2
Cleaved-Caspase3
G11.1+PA
sh-sc
sh-Cox6a2
β-actin

Fig. 1I

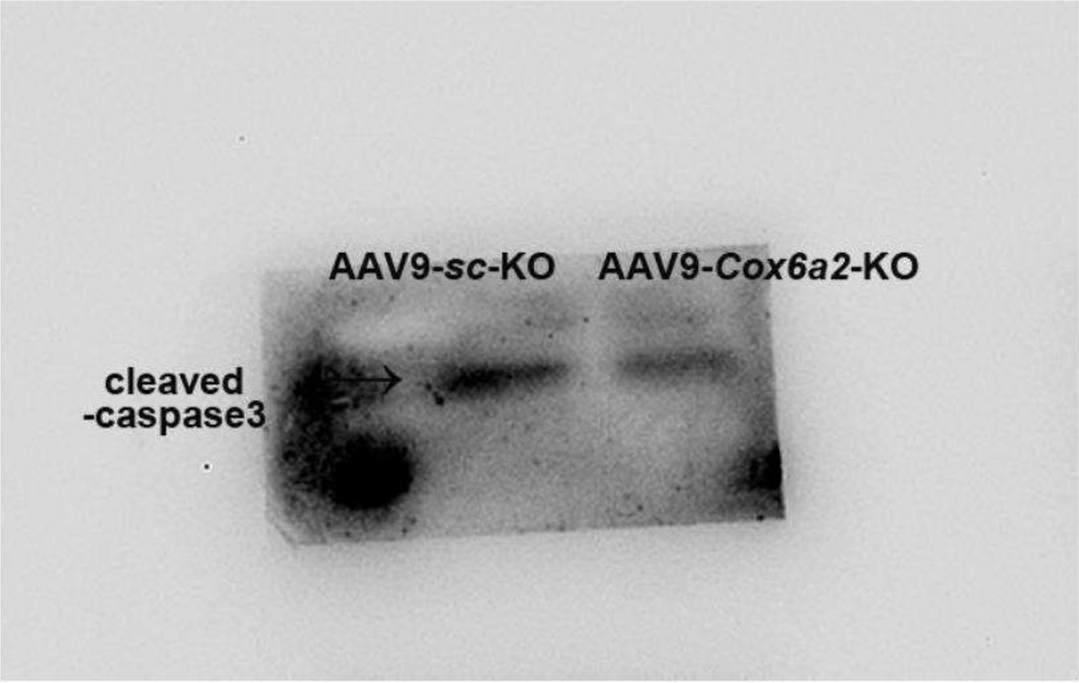
AAV9-*sc*-KO AAV9-*Cox6a2*-KO
cleaved
-caspase3

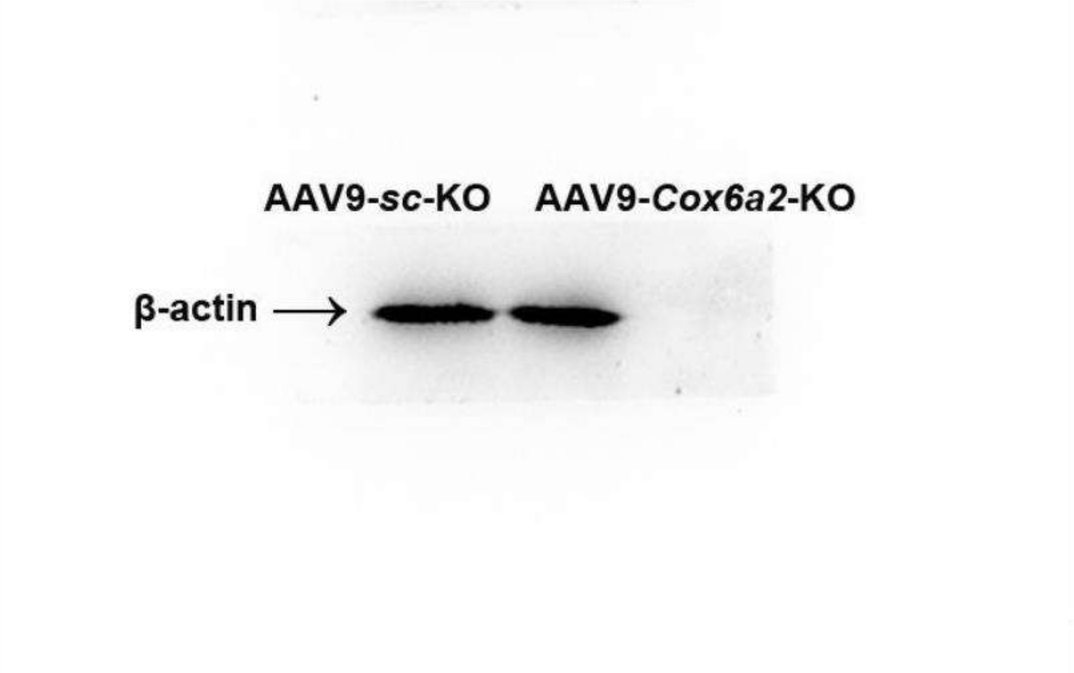
AAV9-*sc*-KO AAV9-*Cox6a2*-KO
β-actin

Fig. 2A

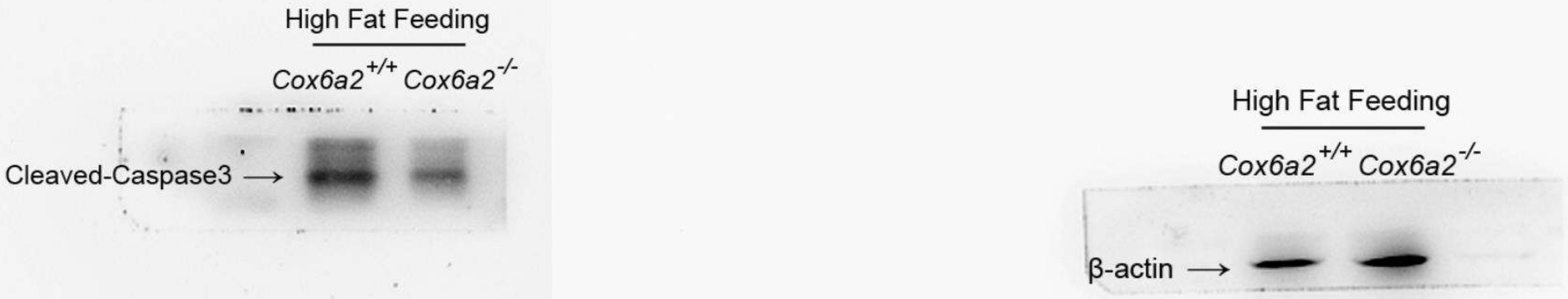
High Fat Feeding
Cox6a2+/+ Cox6a2-/-
Cleaved-Caspase3
High Fat Feeding
Cox6a2+/+ Cox6a2-/-
β-actin

Fig. 3B

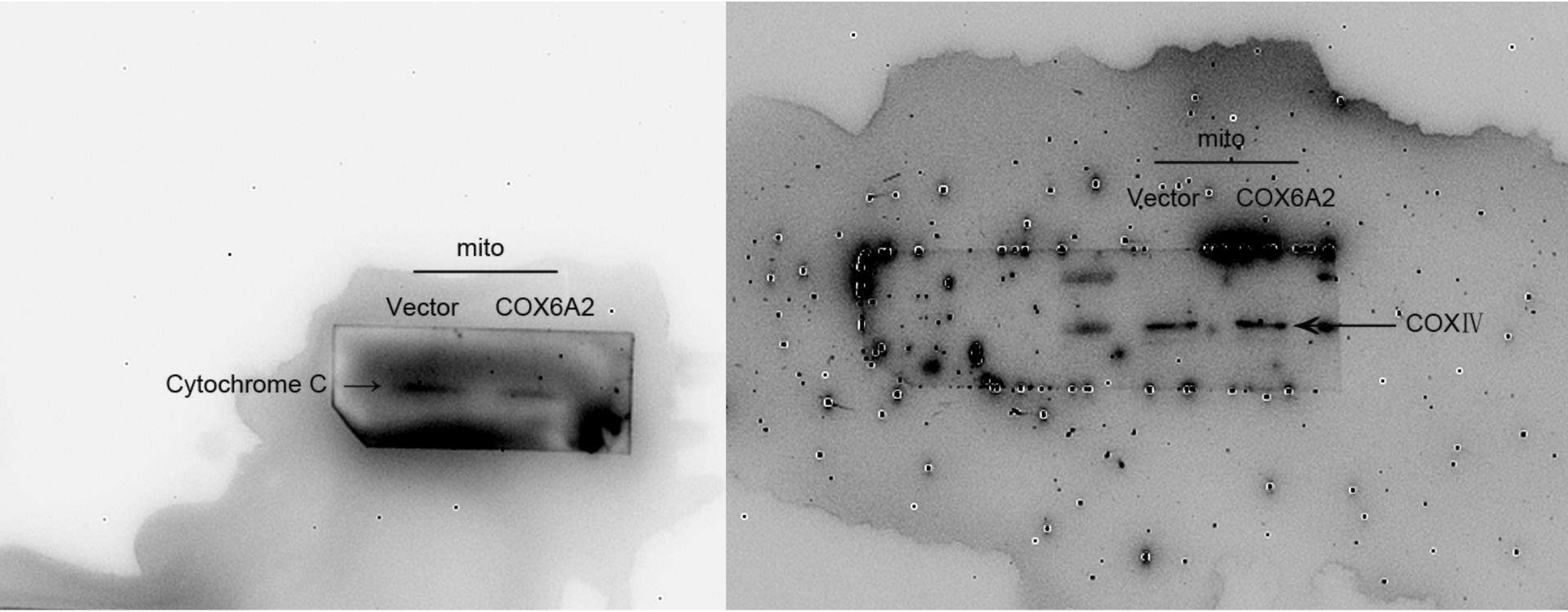

Fig. 3B

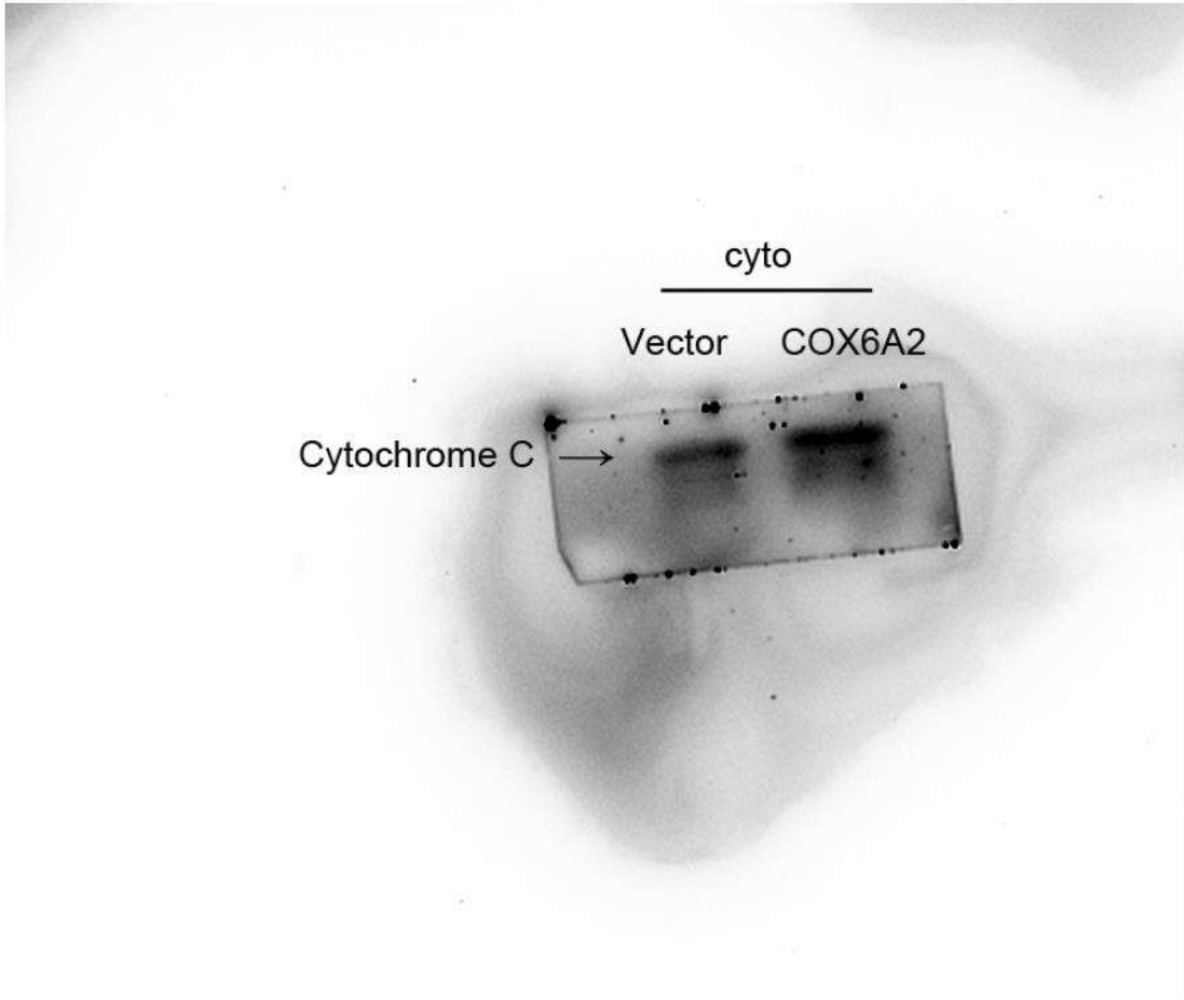

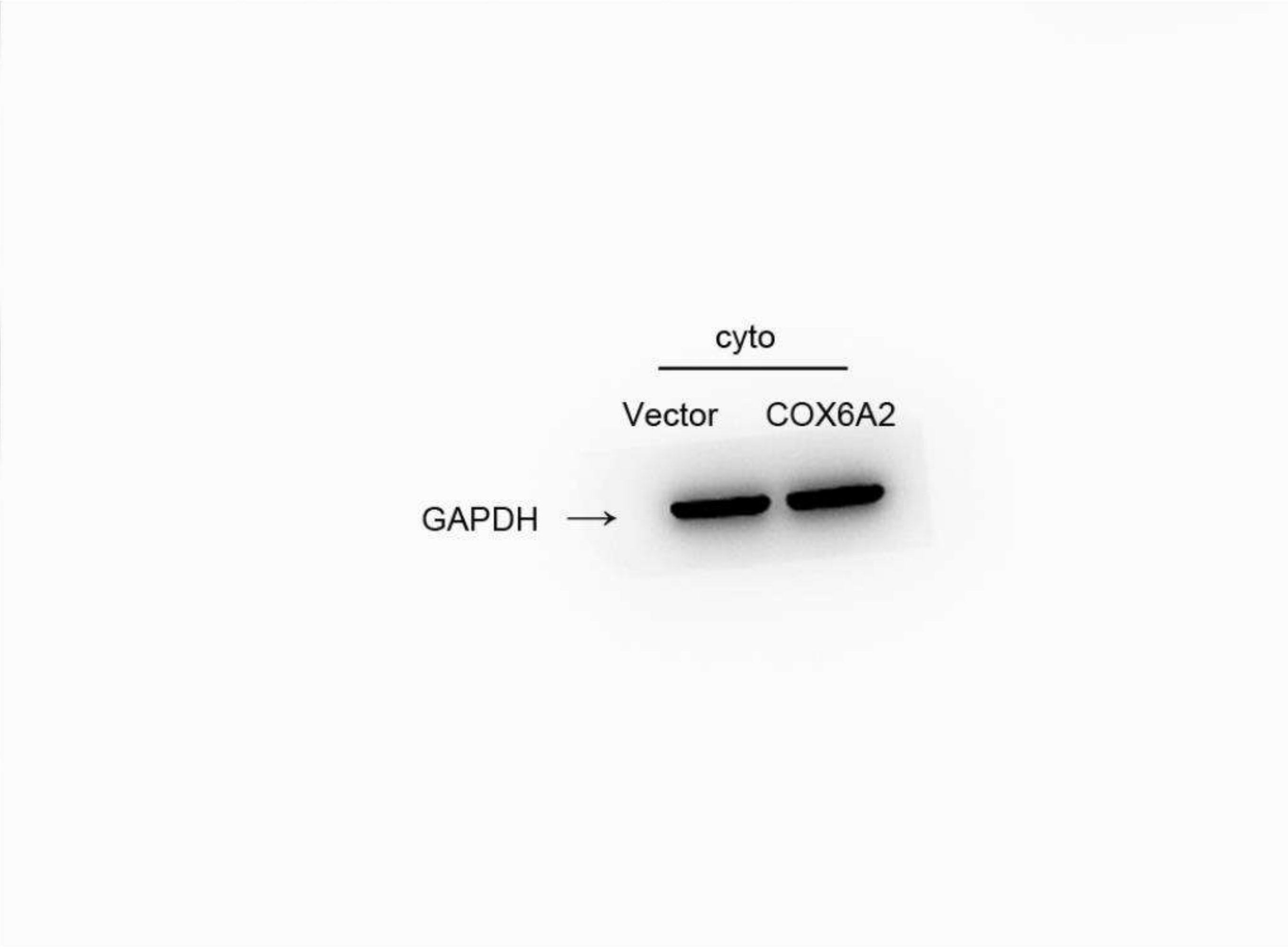

Fig. 3C

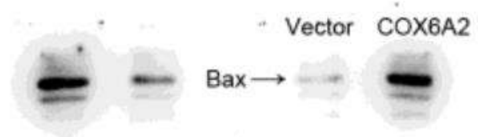

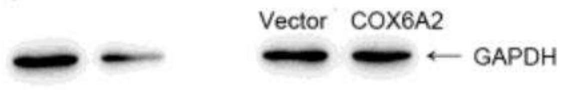

Fig. 3C

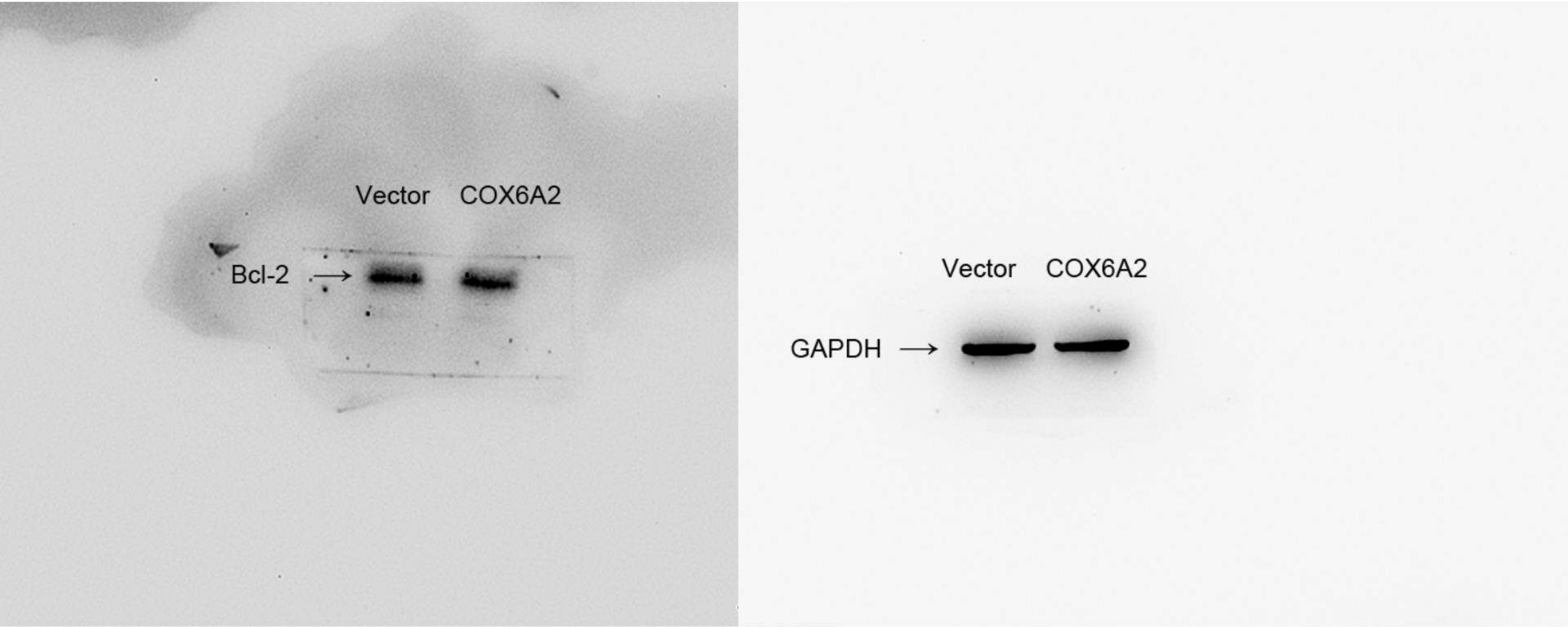

Fig. 3D

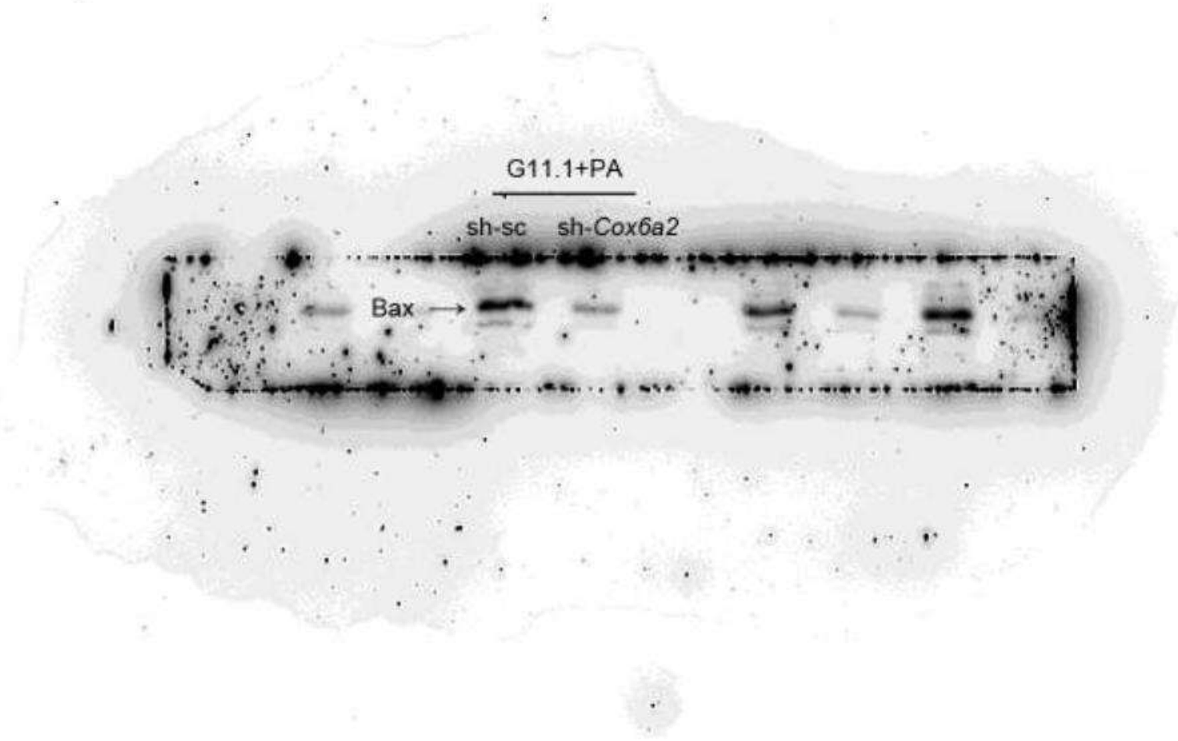

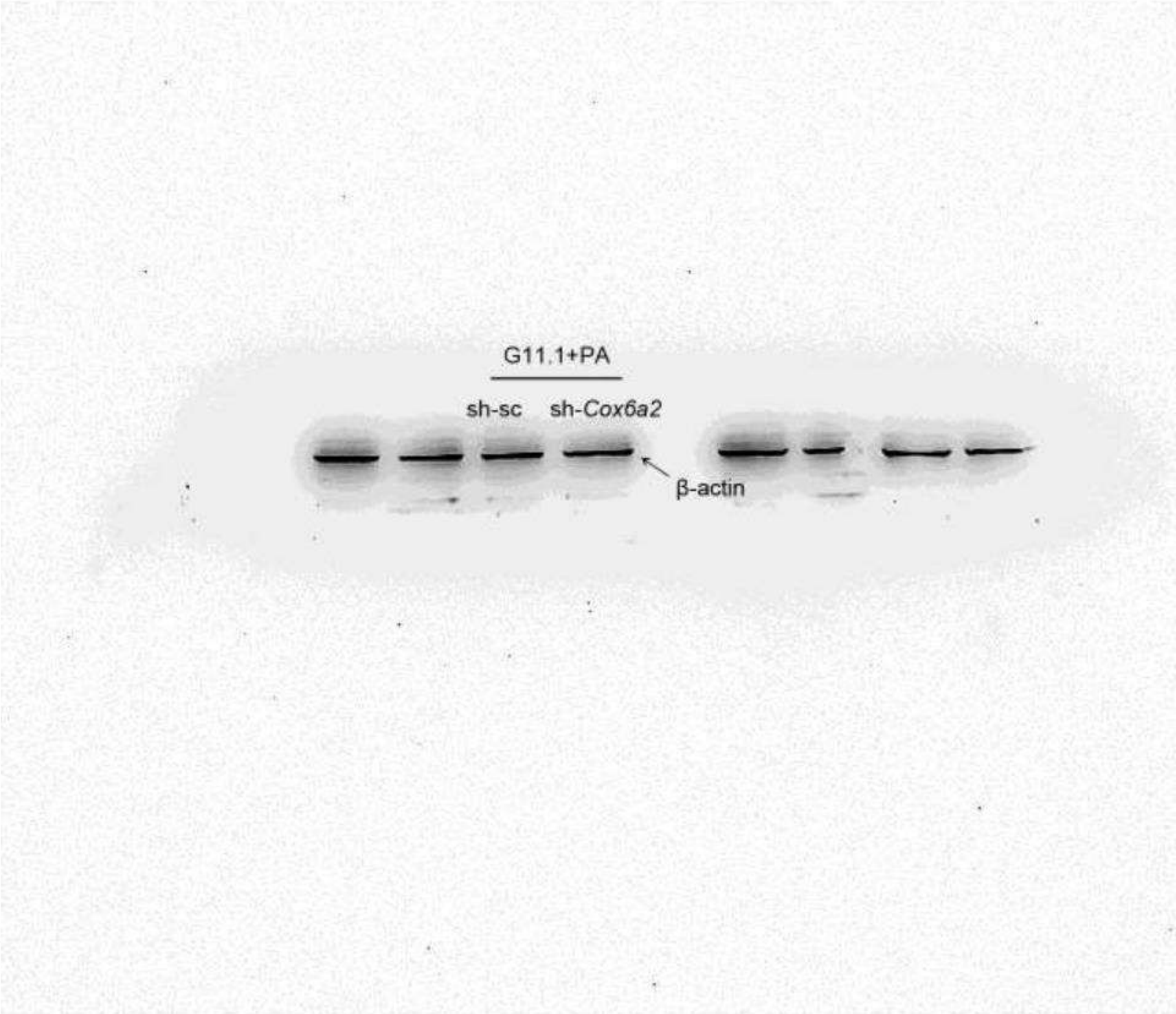

Fig. 3D

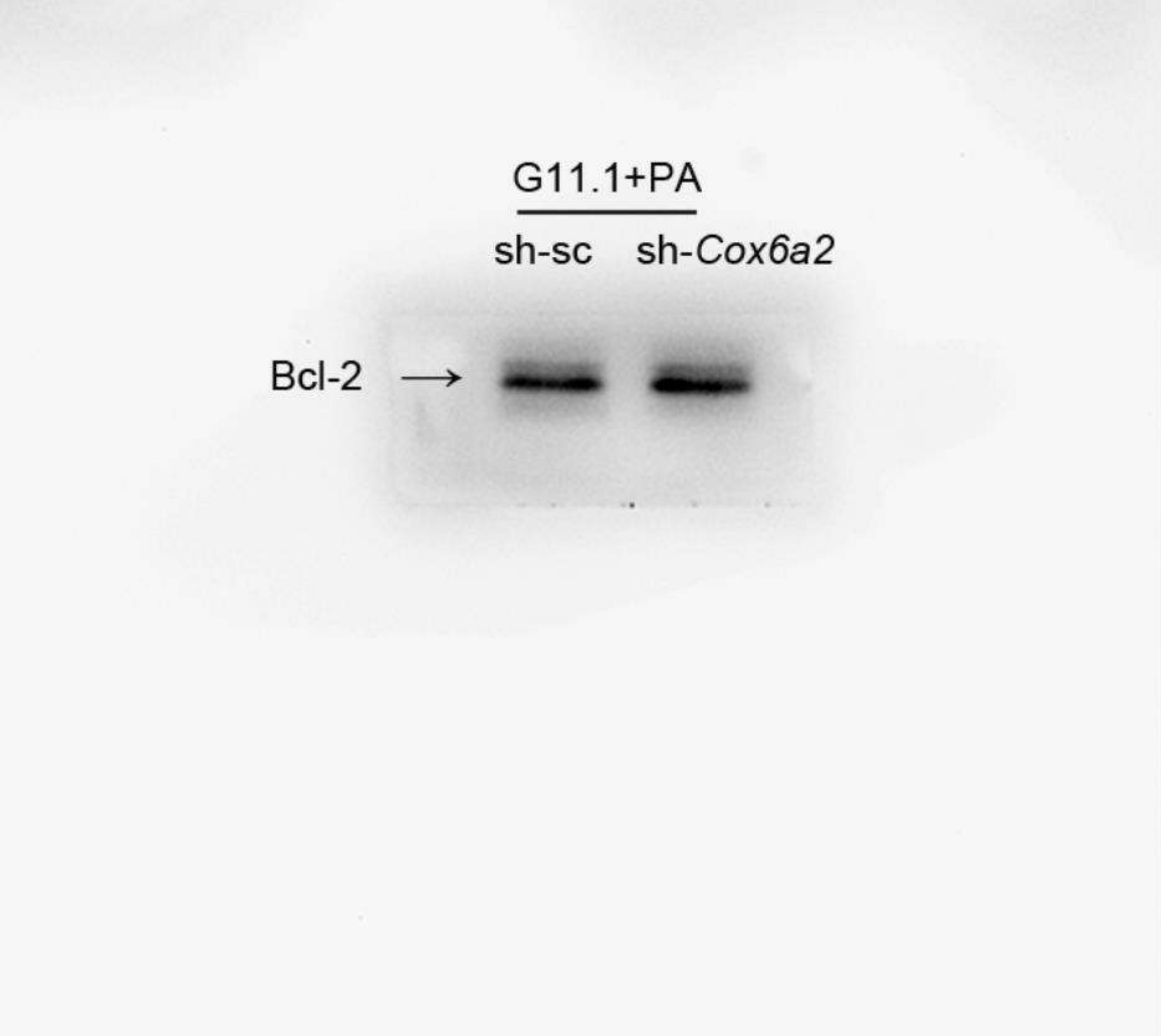

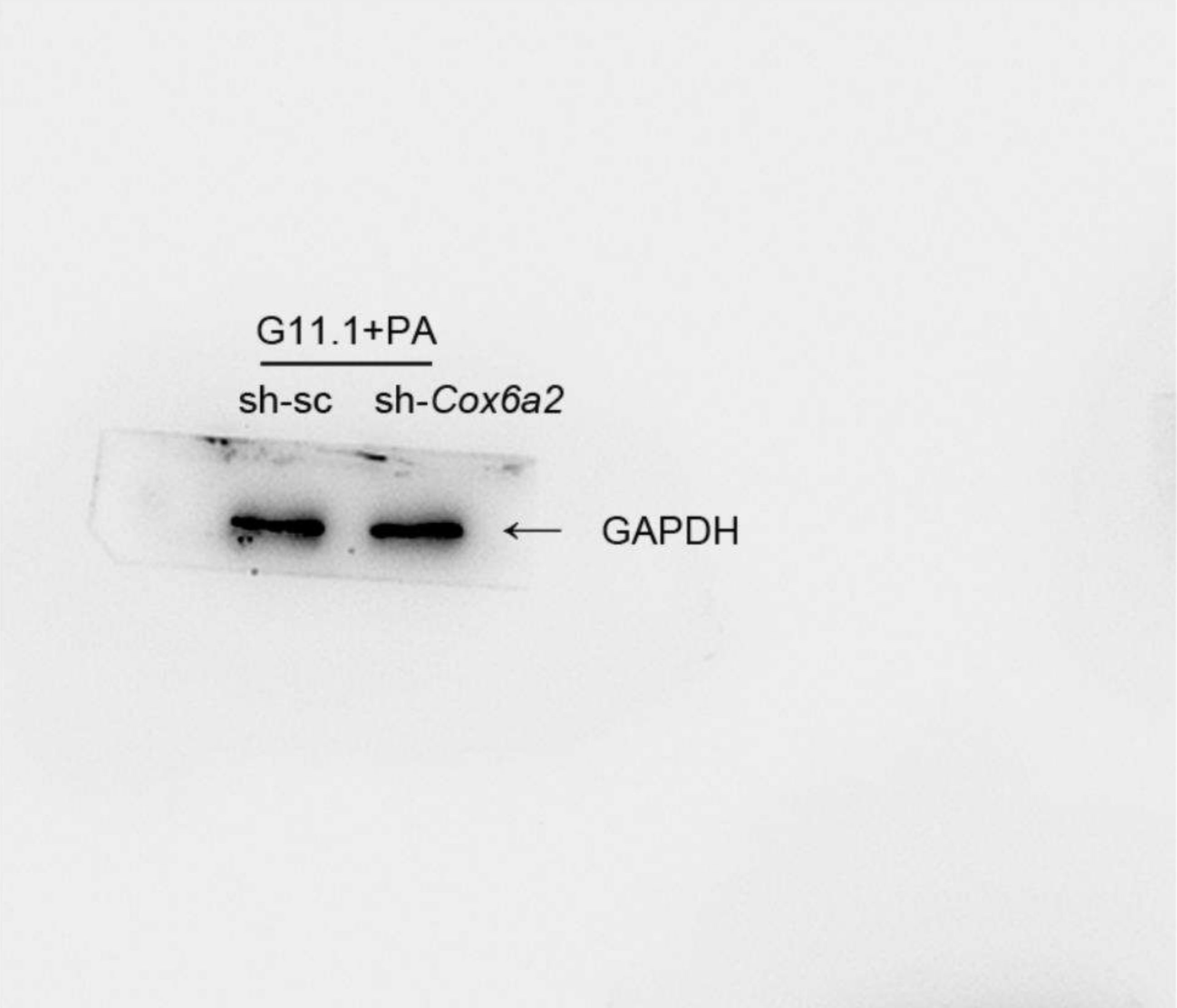

Fig. 3E

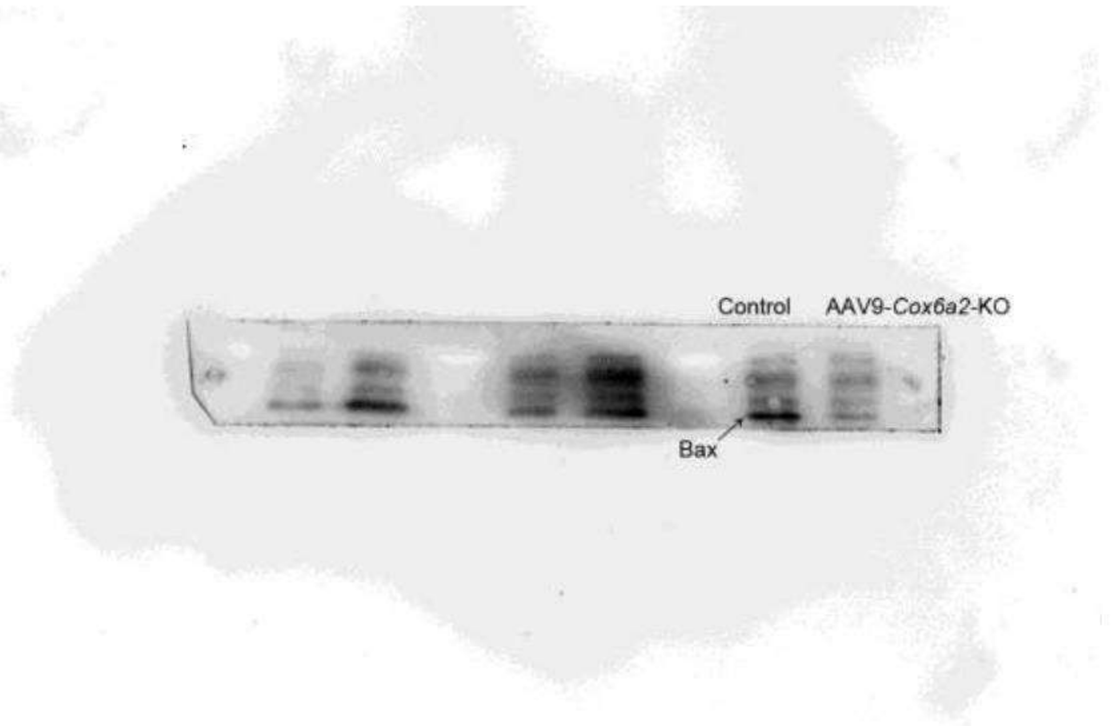

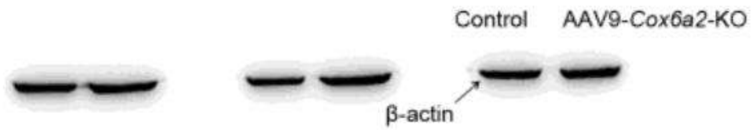

Fig. 3F

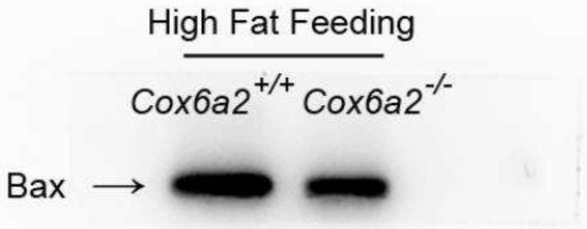
High Fat Feeding
Cox6a2+/+ Cox6a2-/-
Bax

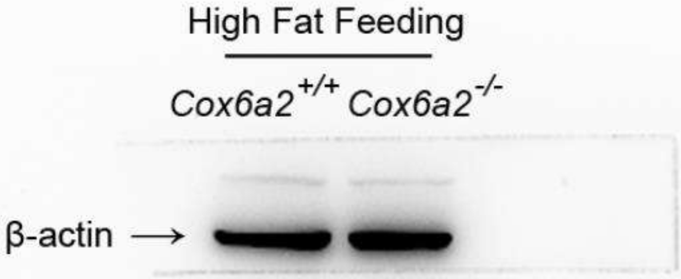
High Fat Feeding
Cox6a2+/+ Cox6a2-/-
β-actin

Fig. 3G

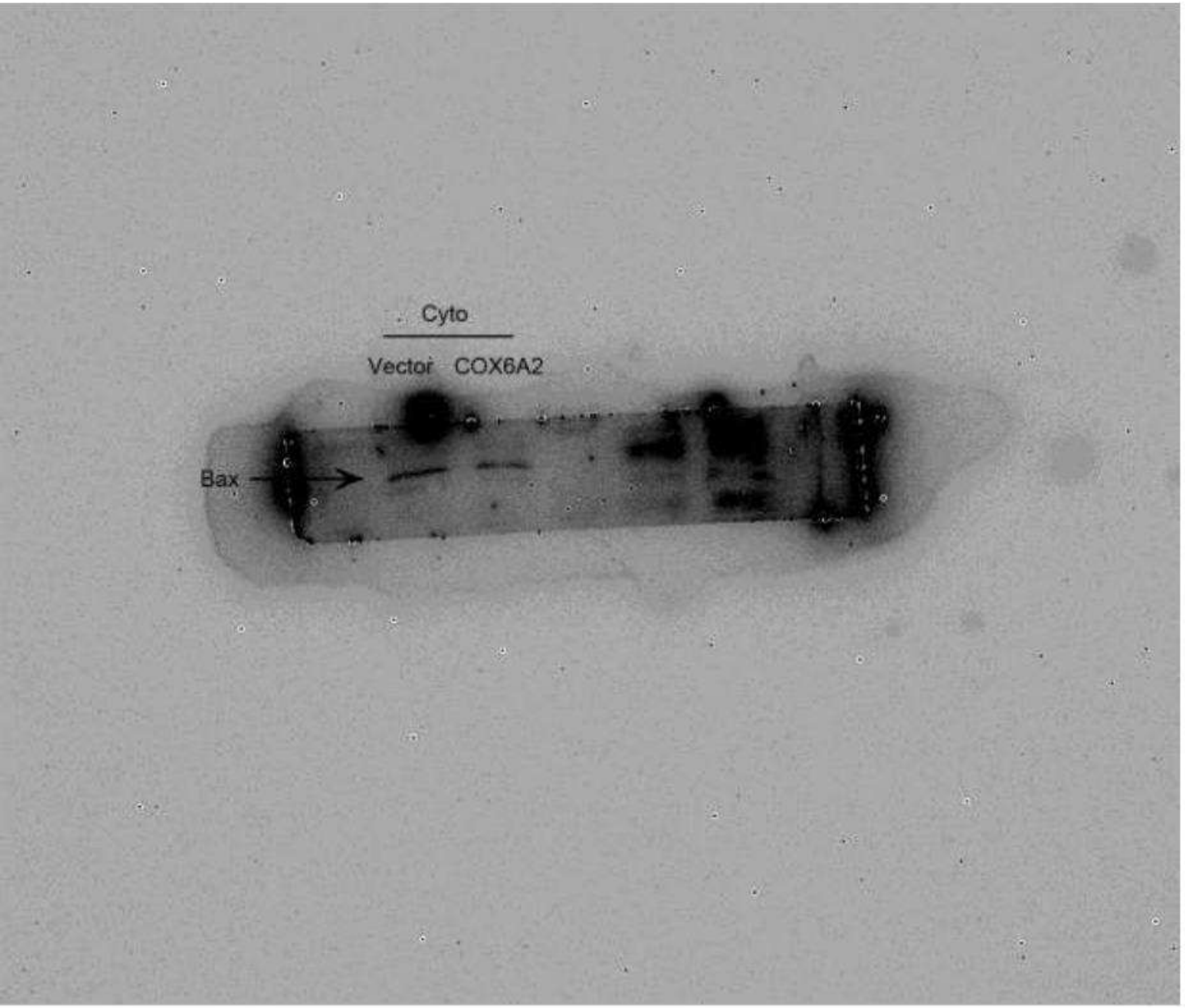

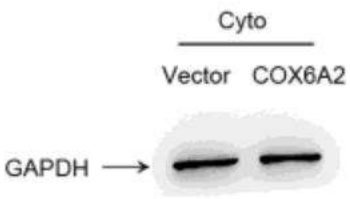

Fig. 3G

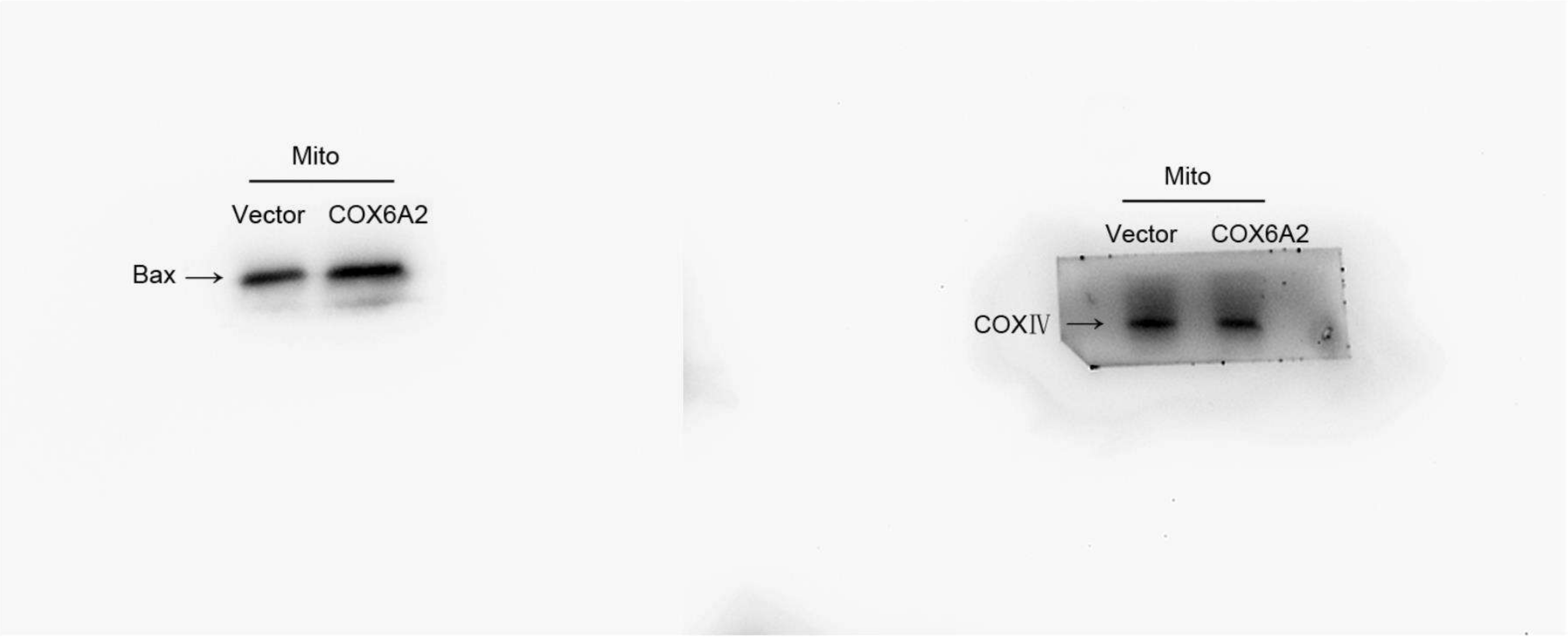

Fig. 4B

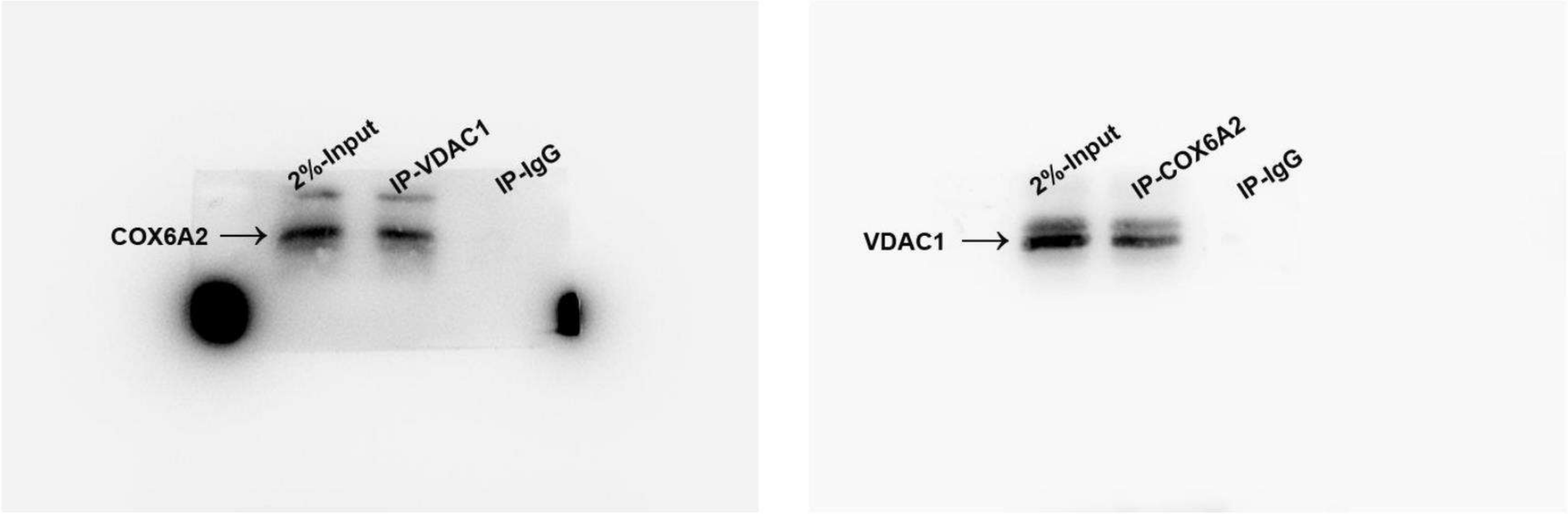

Fig. 4C

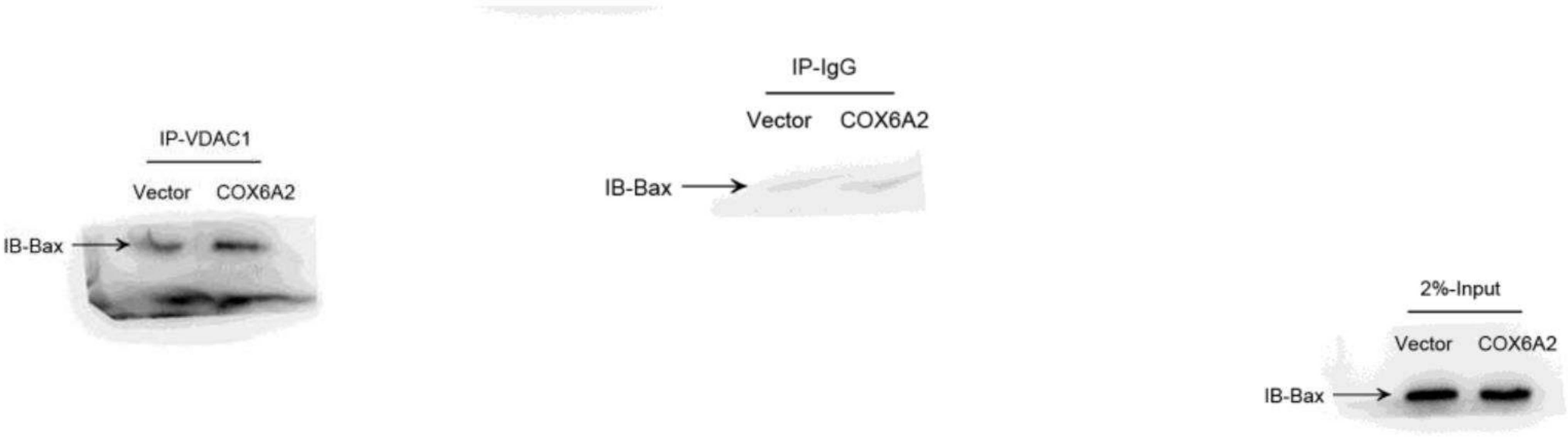
IP-VDAC1
Vector
COX6A2
IB-Bax
IP-IgG
Vector
COX6A2
IB-Bax
2%-Input
Vector
COX6A2
IB-Bax

Fig. 4C

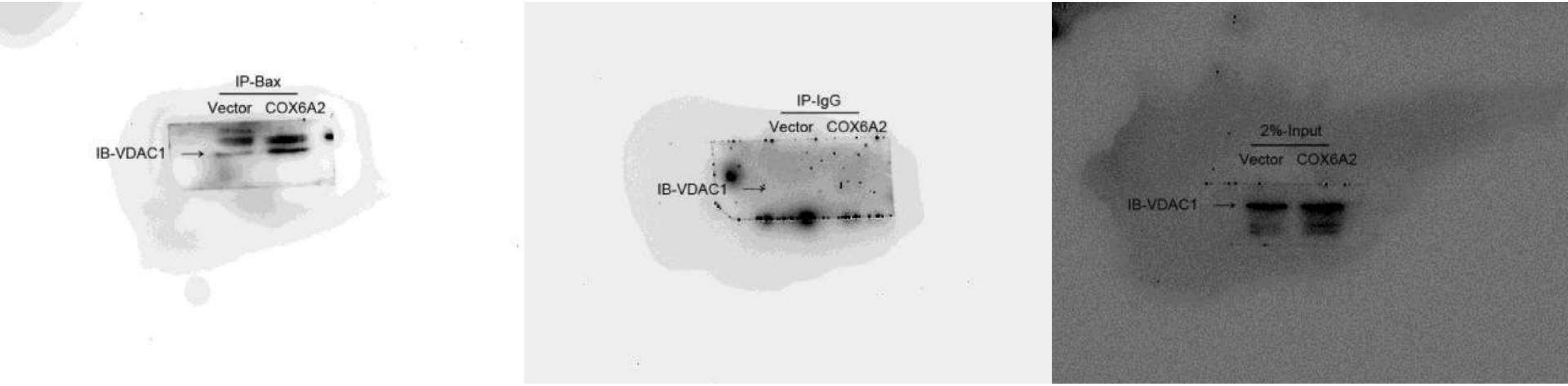
IP-Bax
Vector
COX6A2
IB-VDAC1
IP-IgG
Vector
COX6A2
IB-VDAC1
2%-Input
Vector
COX6A2
IB-VDAC1

Fig. 4D

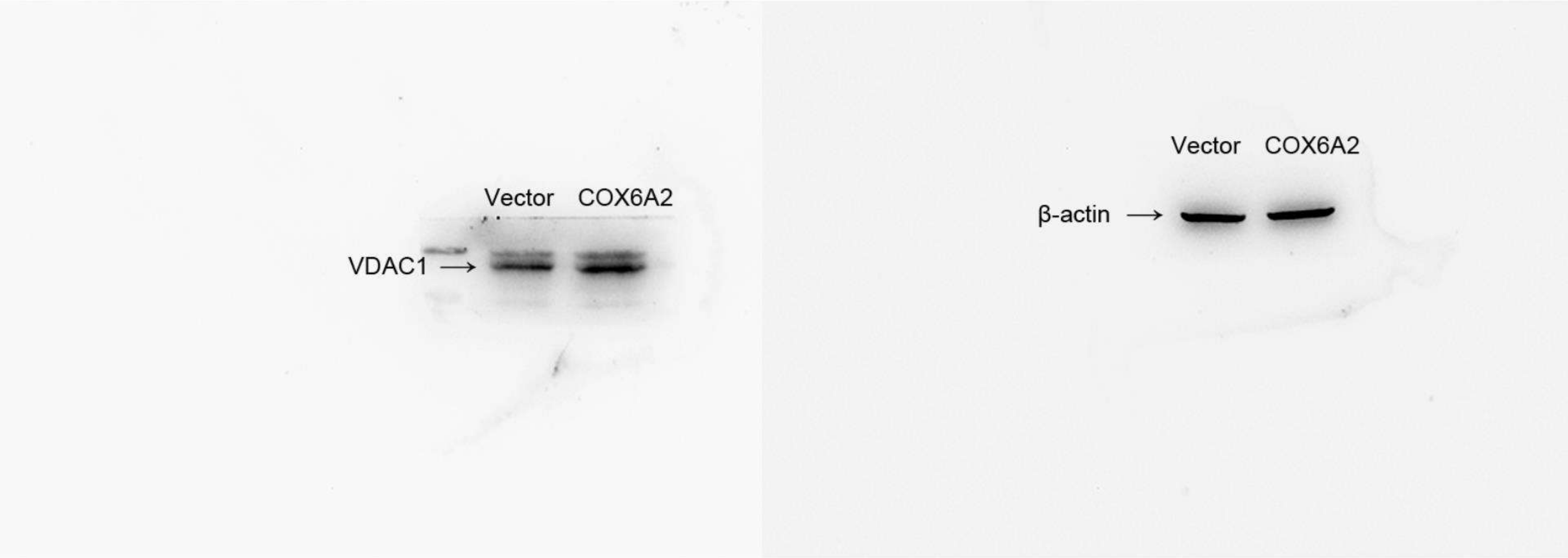

Fig. 4D

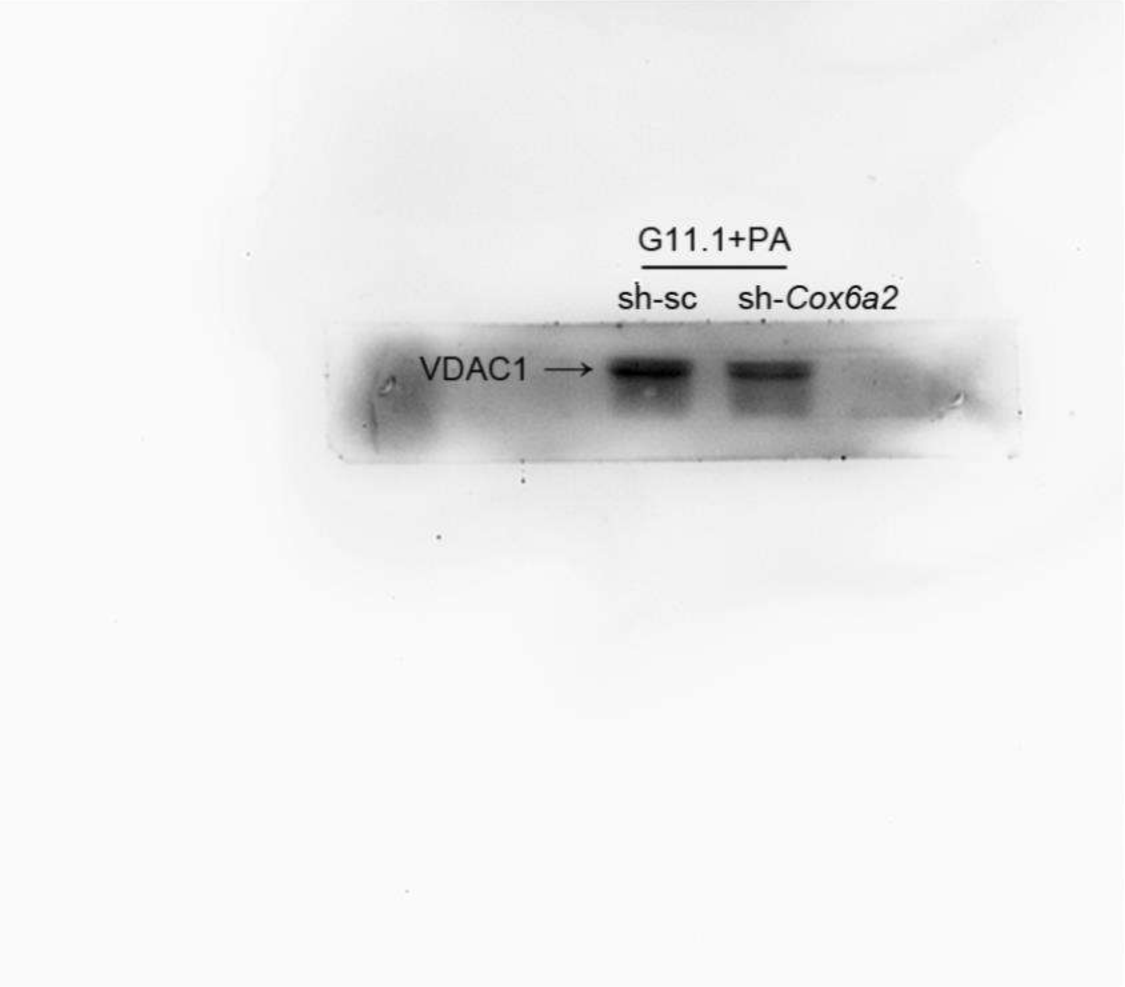

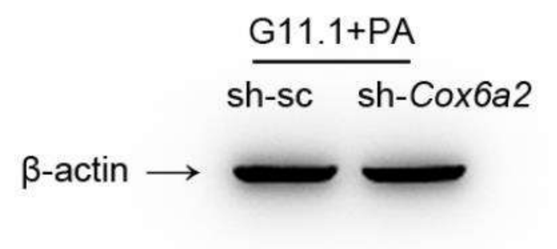

Fig. 4E

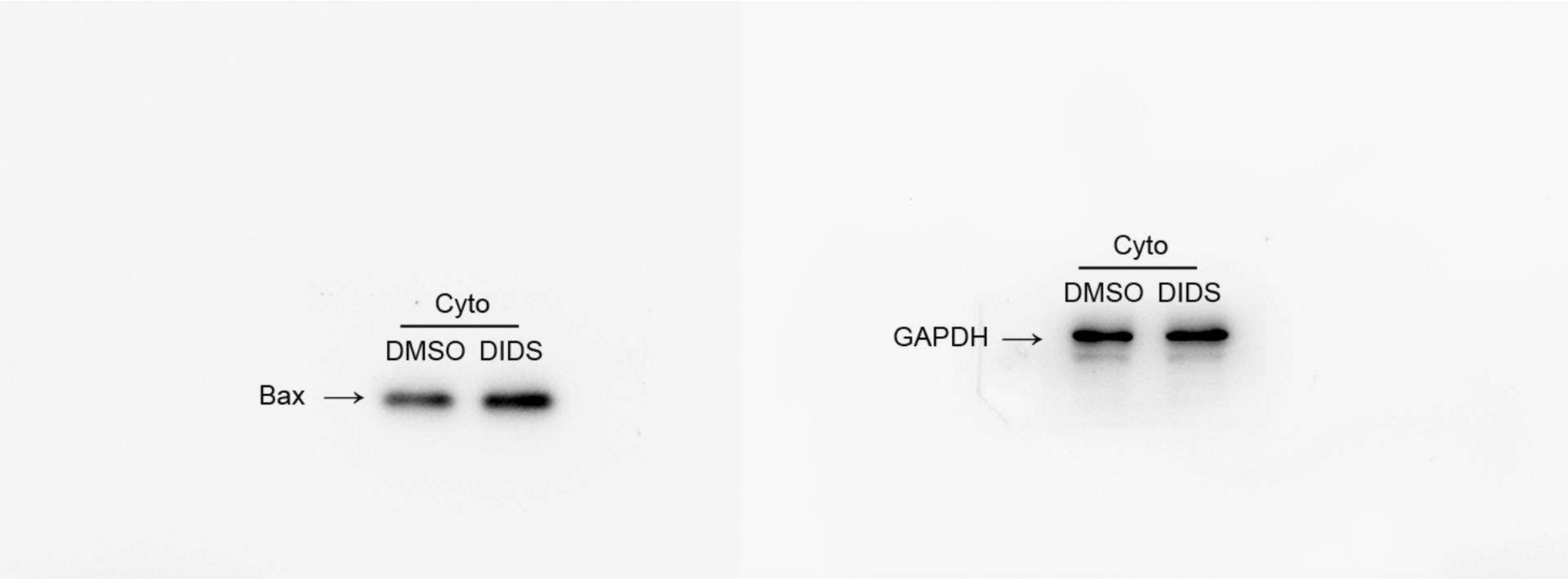

Fig. 4E

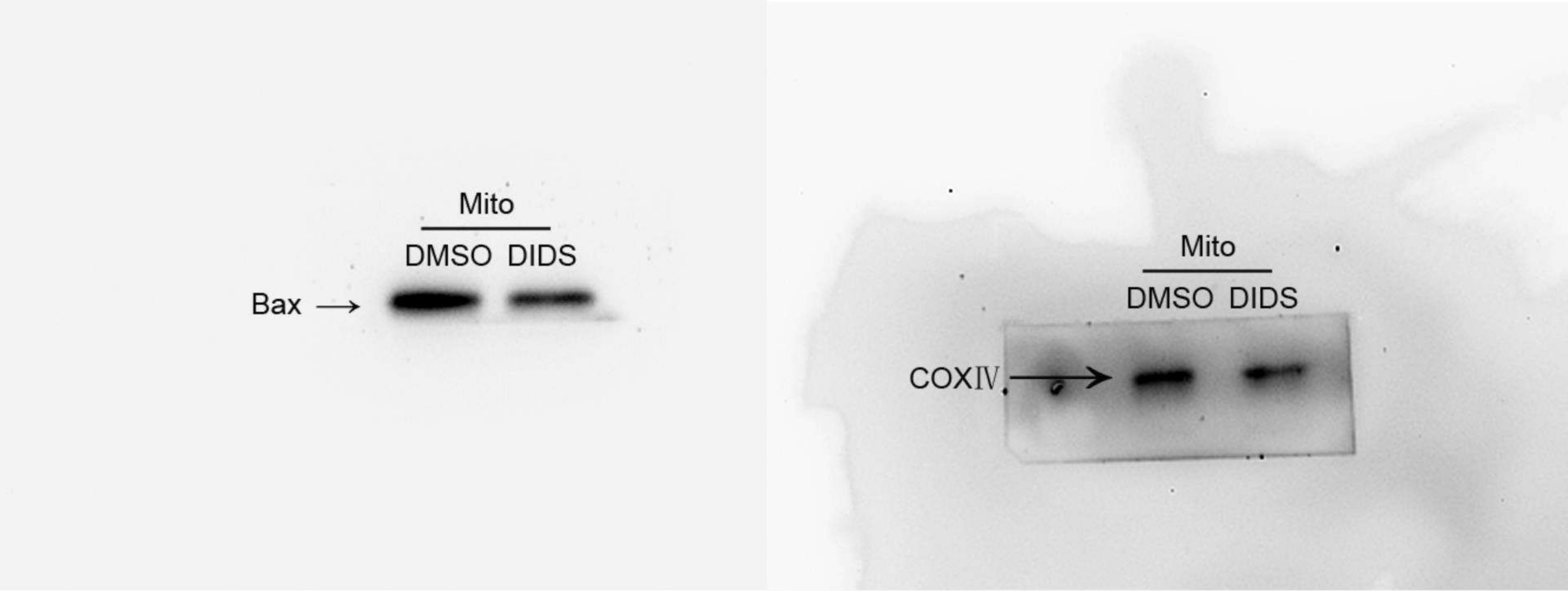

Fig. 4F

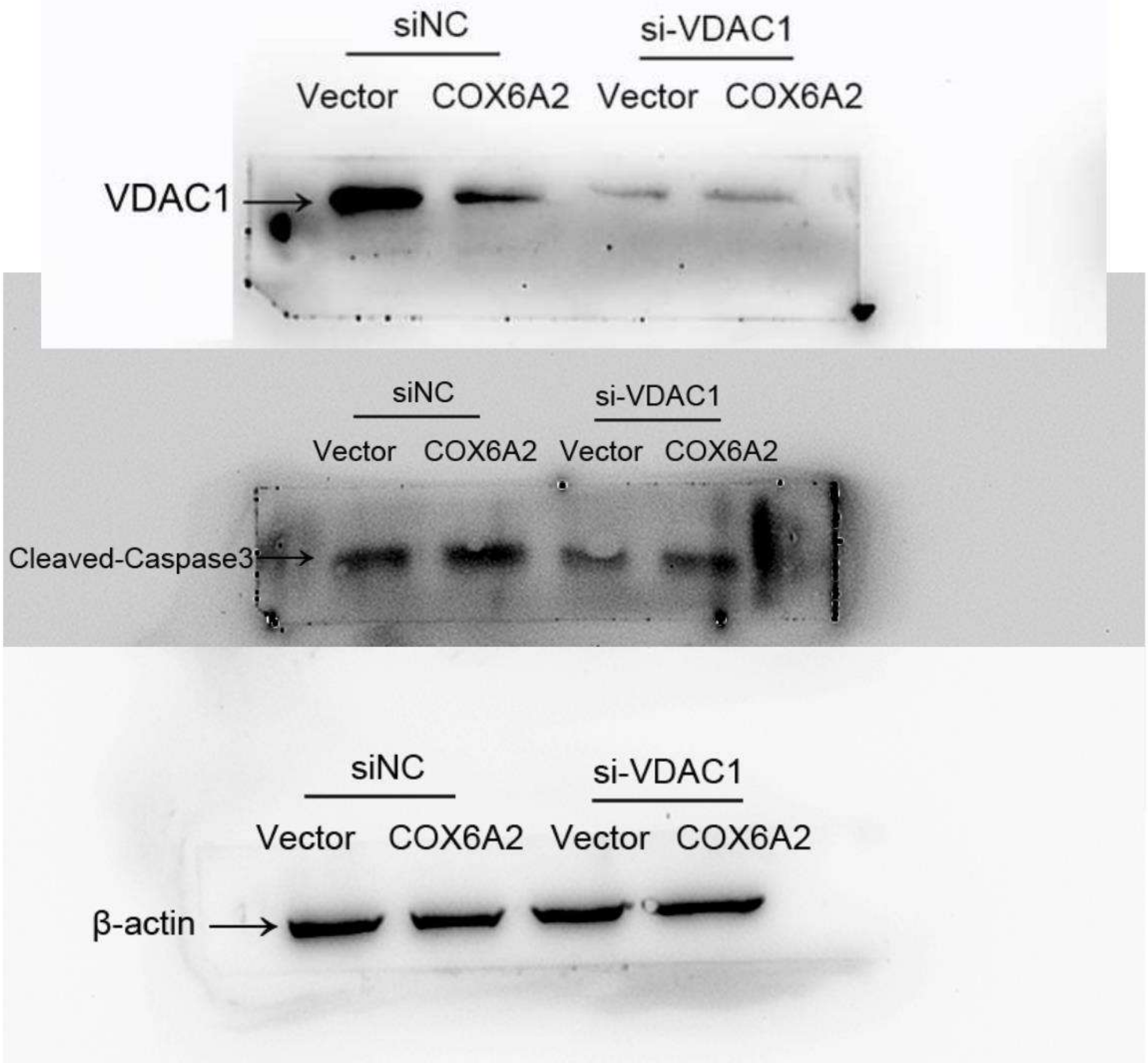
siNC
si-VDAC1
Vector
COX6A2
Vector
COX6A2
VDAC1
siNC
si-VDAC1
Vector
COX6A2
Vector
COX6A2
Cleaved-Caspase3
siNC
si-VDAC1
Vector
COX6A2
Vector
COX6A2
β-actin

Fig. 4G

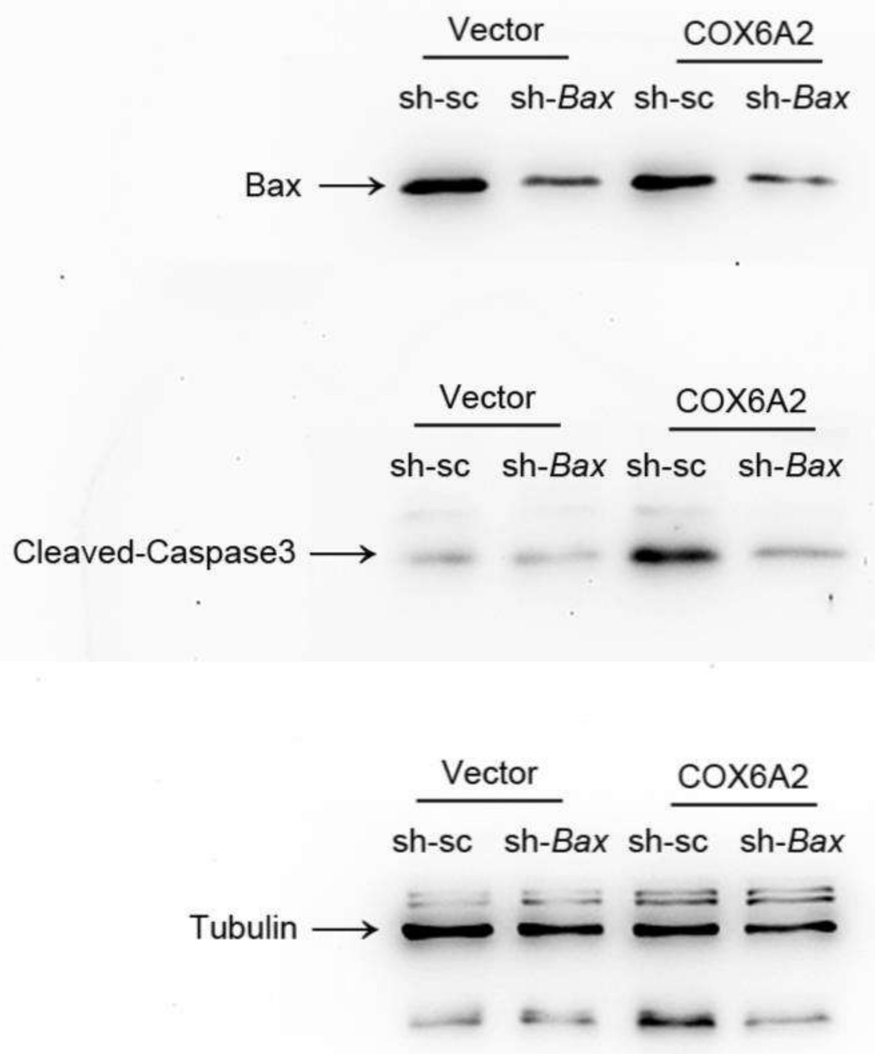

Vector
COX6A2
sh-sc
sh-*Bax*
sh-sc
sh-*Bax*
Bax
Vector
COX6A2
sh-sc
sh-*Bax*
sh-sc
sh-*Bax*
Cleaved-Caspase3
Vector
COX6A2
sh-sc
sh-*Bax*
sh-sc
sh-*Bax*
Tubulin

Fig. 5D

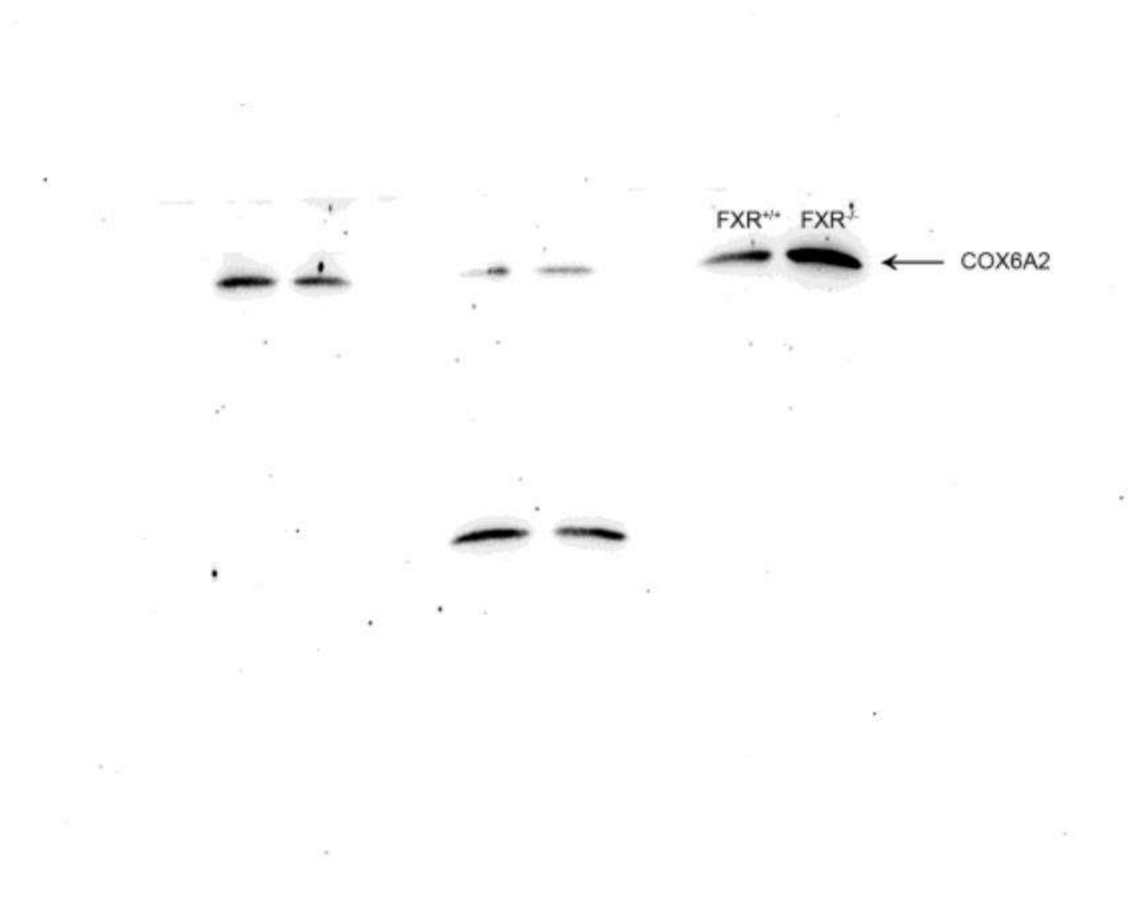

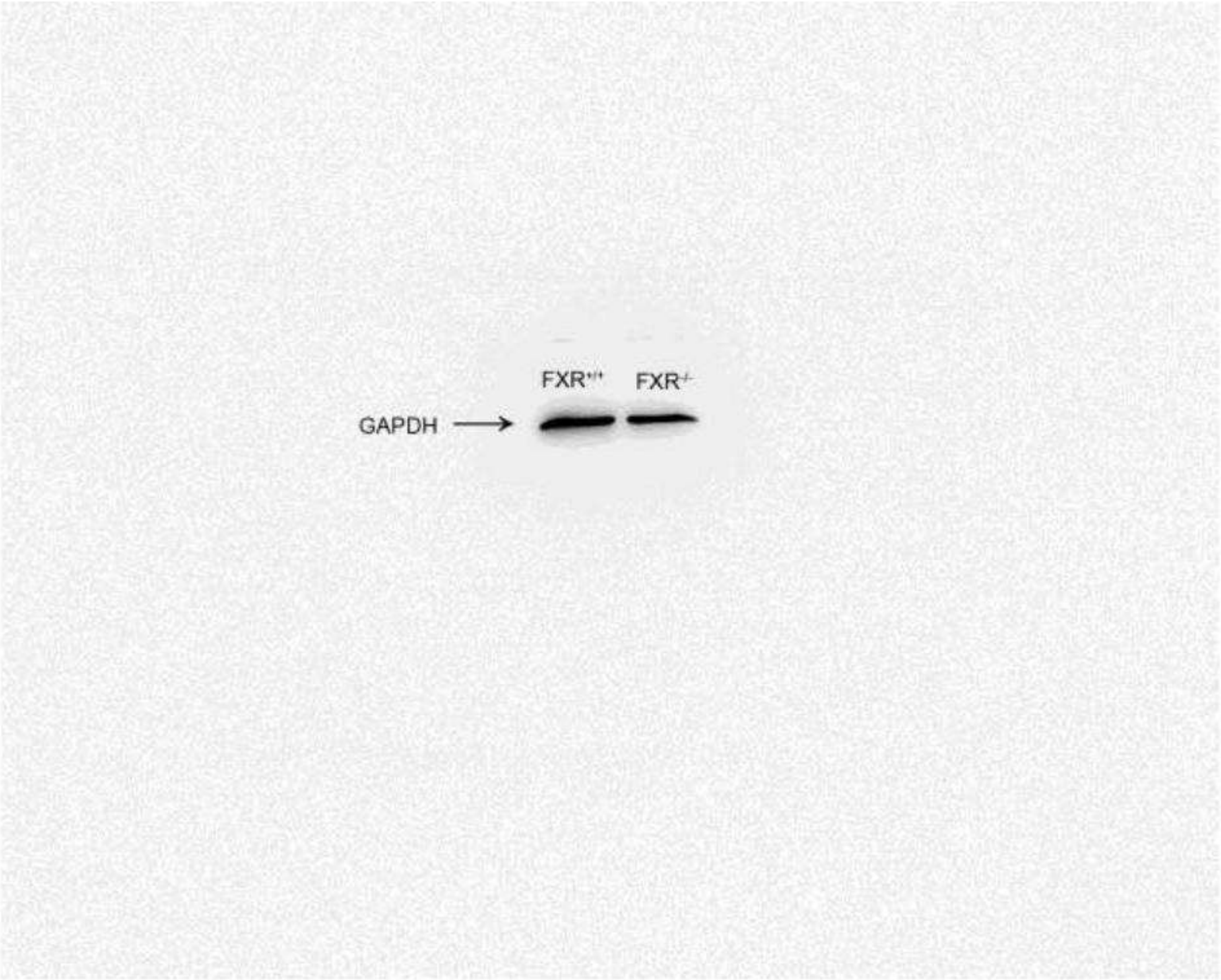

Fig. 5F

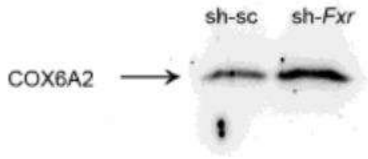

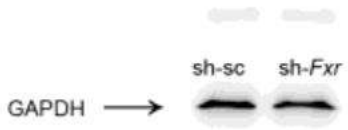

Fig. 5G

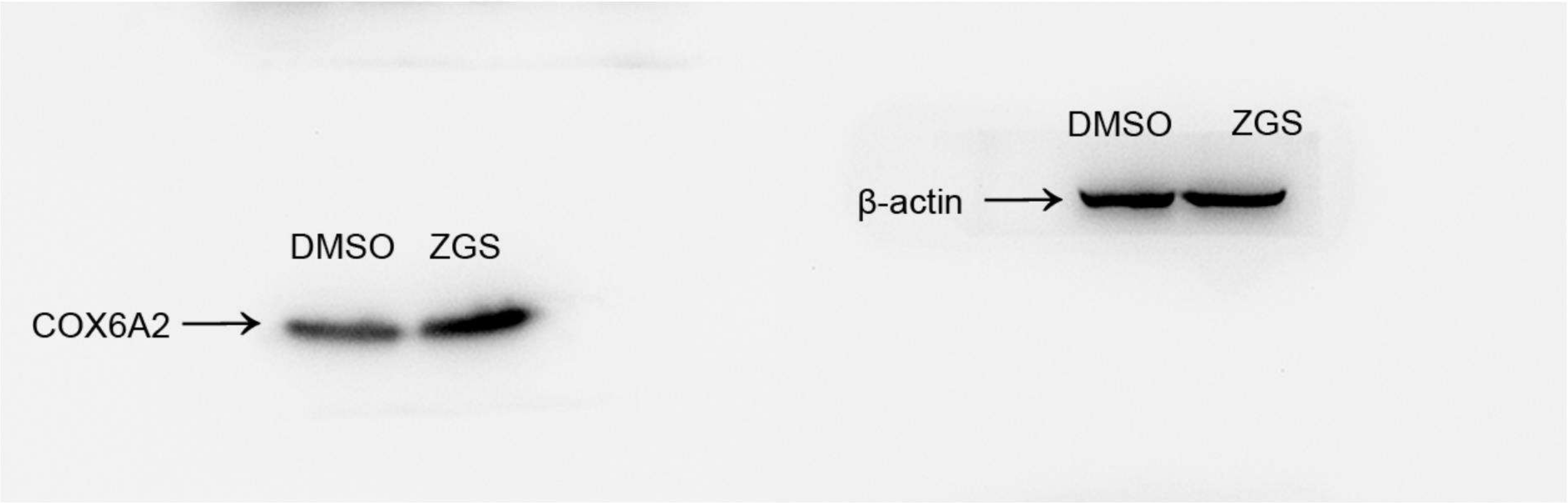

Fig. 5I

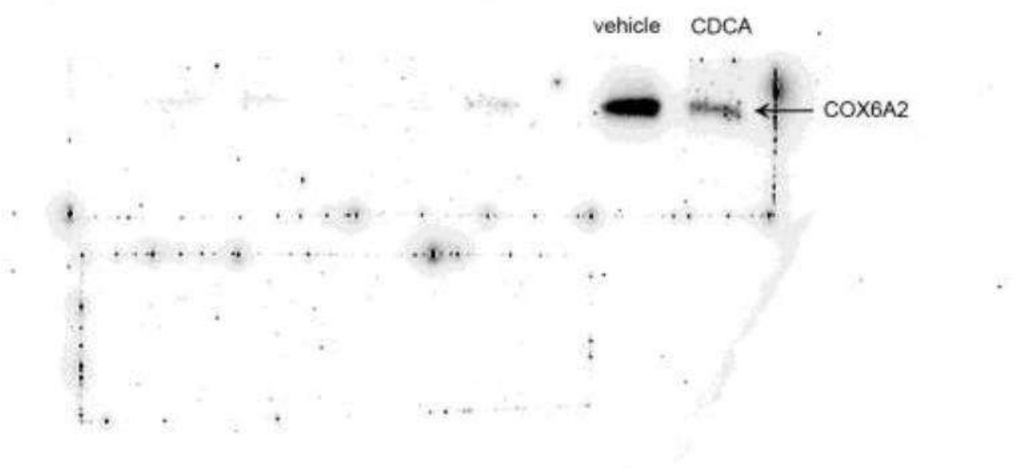

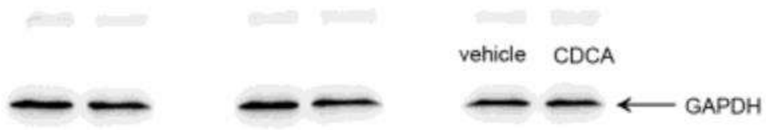

Fig. 6A

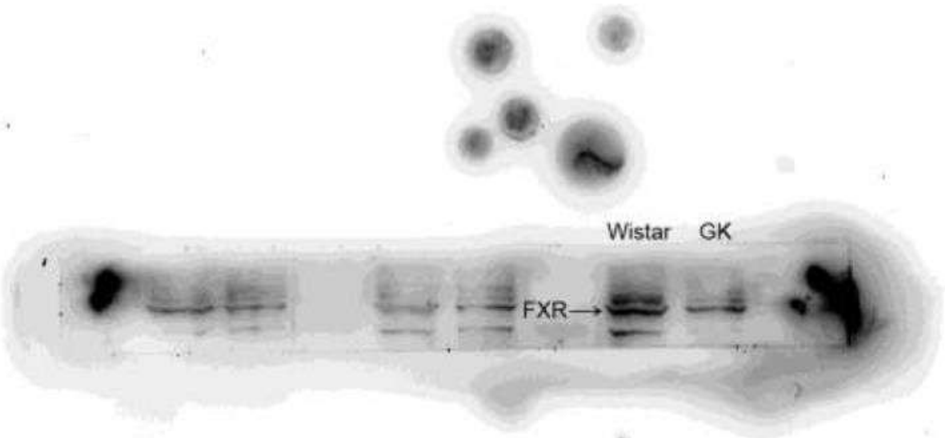

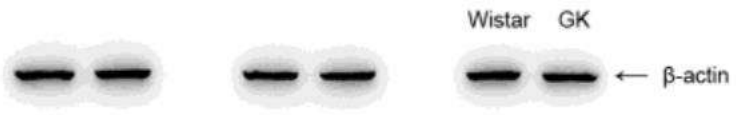

Fig. 6B

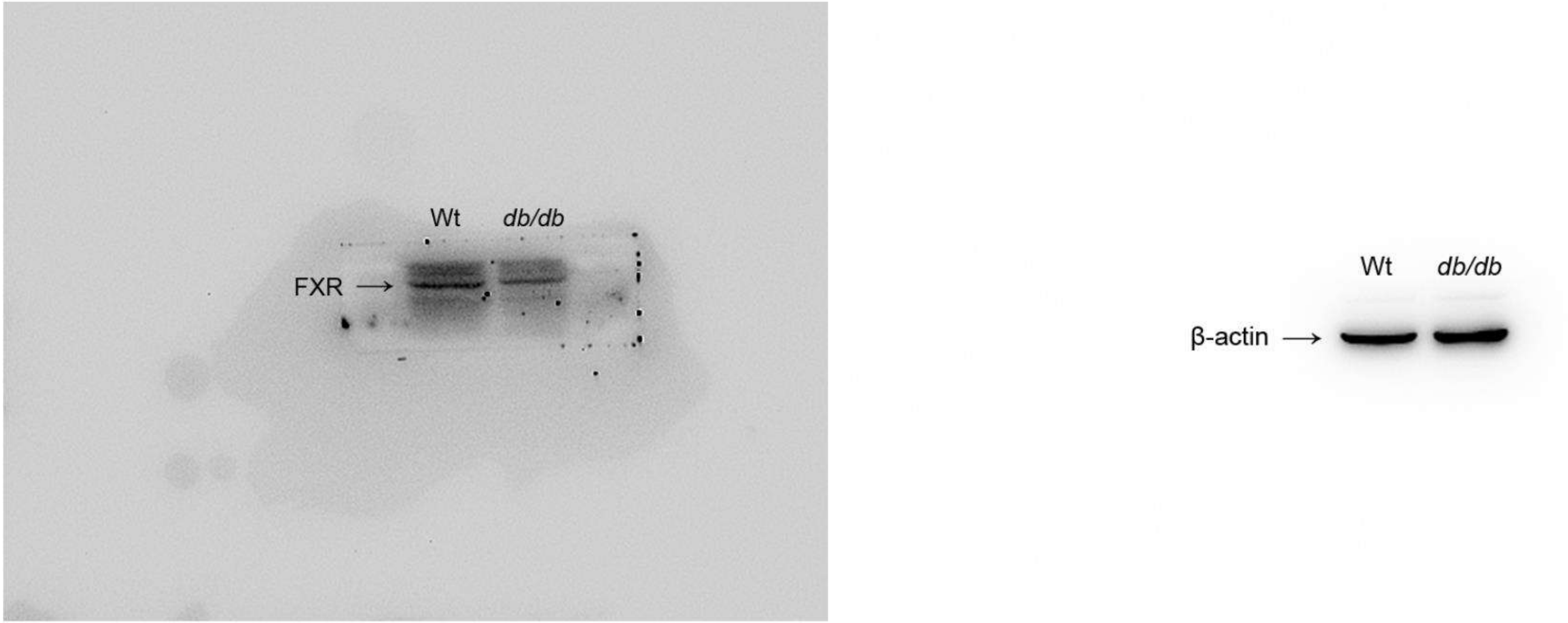

Fig. 6C

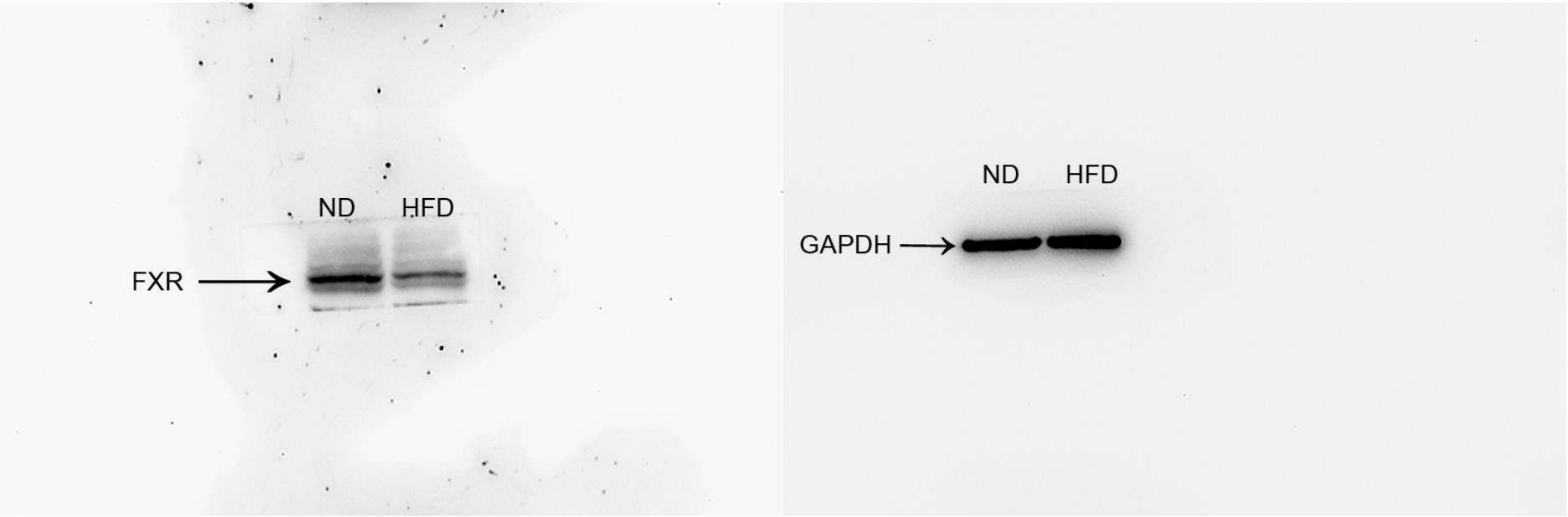

Fig. 6D

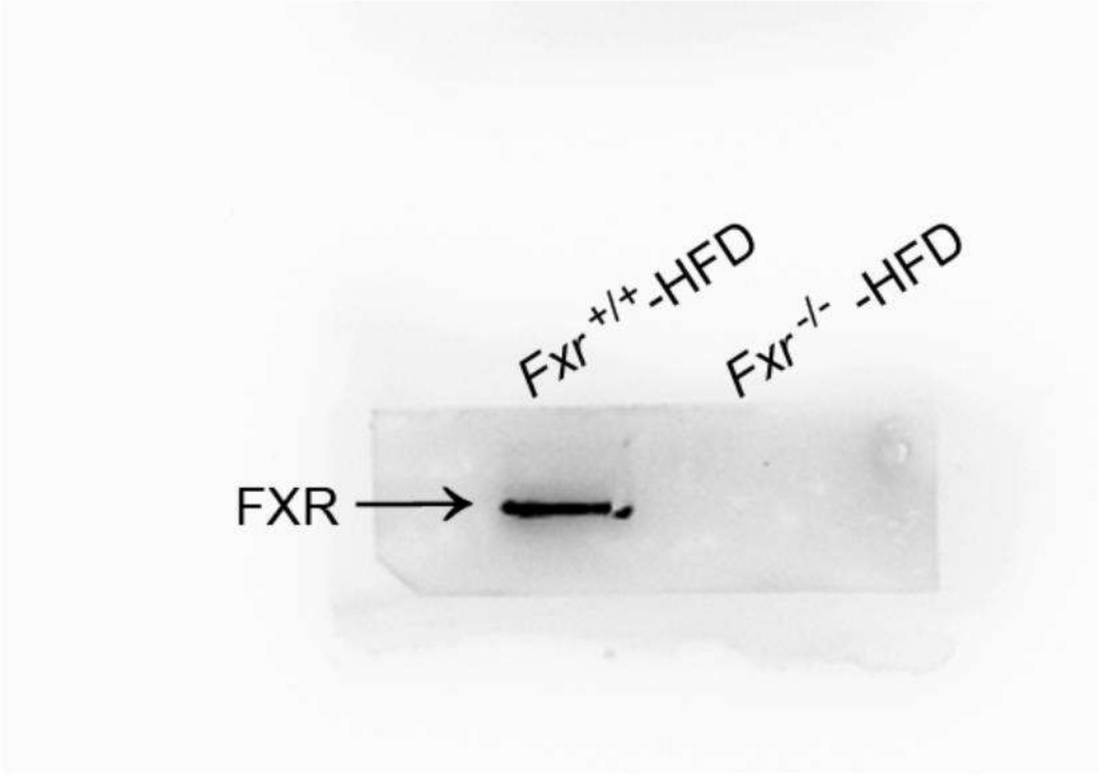

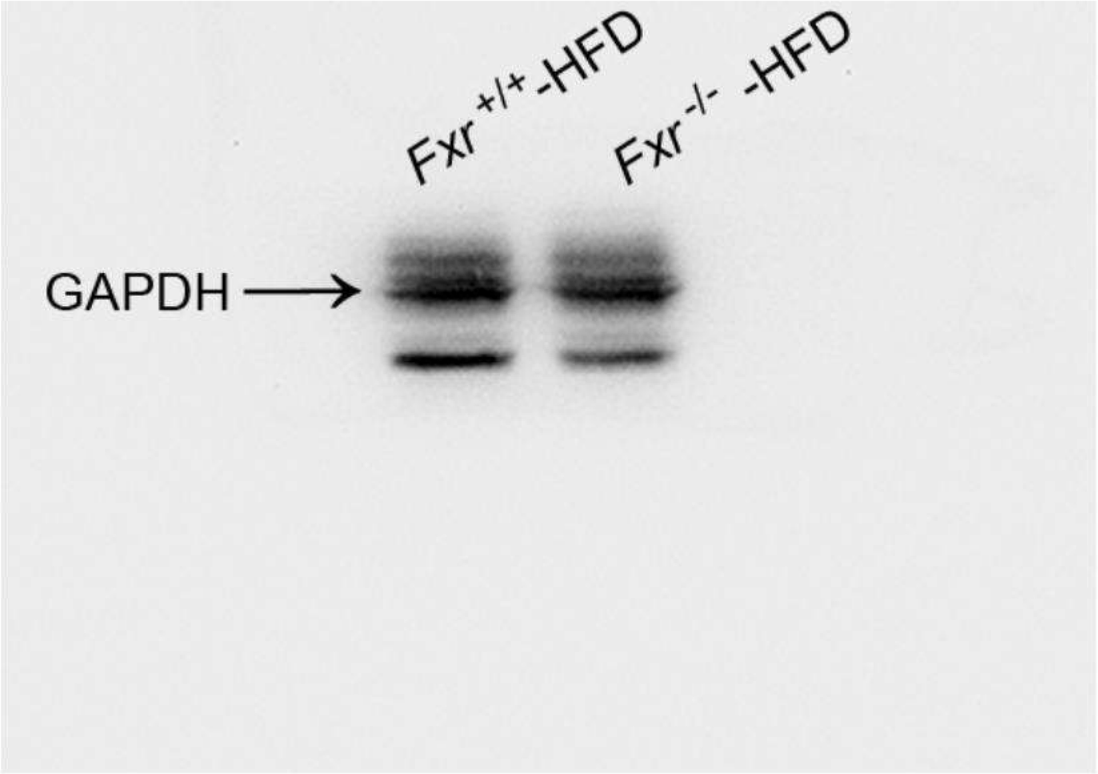

Fig. 6F

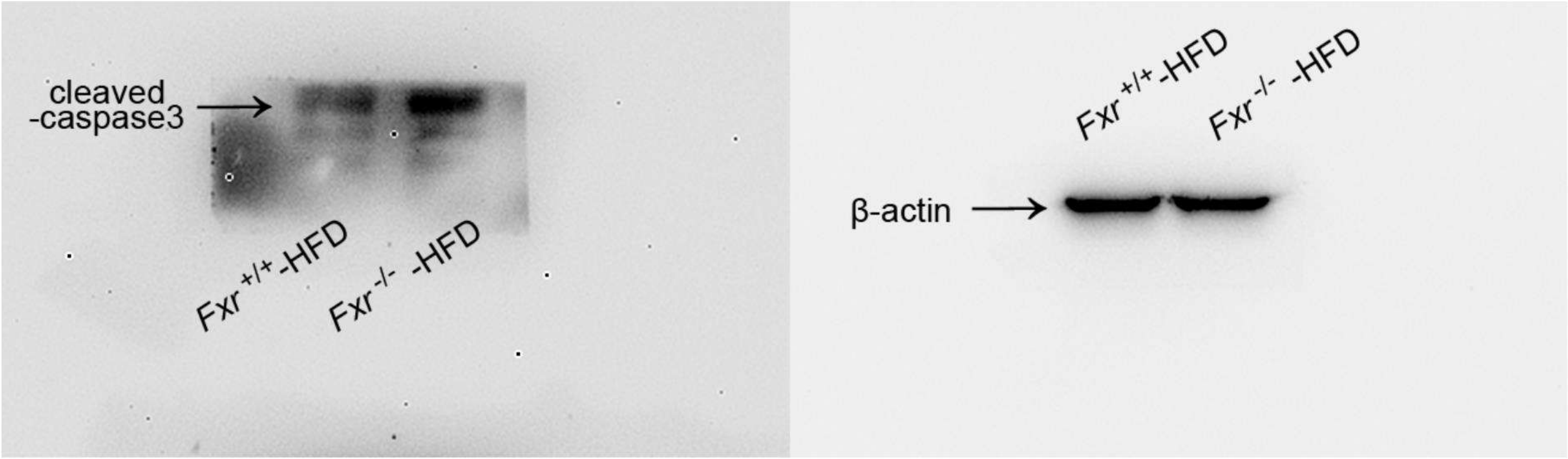

Fig. 6M

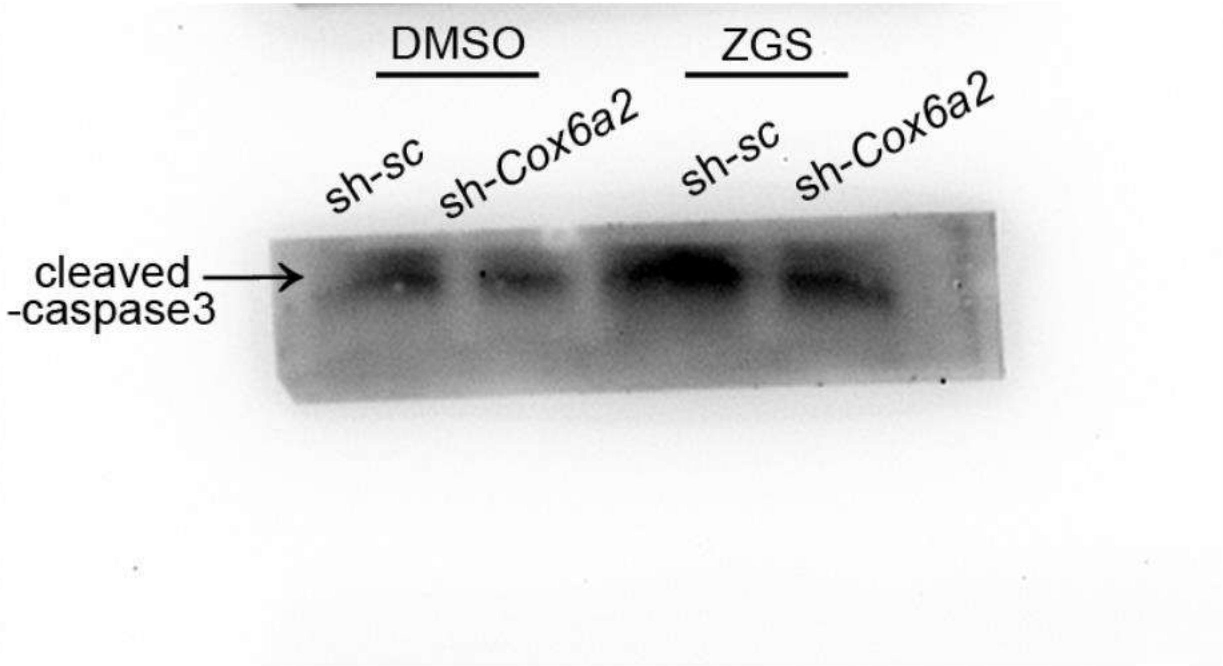
DMSO
ZGS
sh-sc
sh-Cox6a2
sh-sc
sh-Cox6a2
cleaved
-caspase3

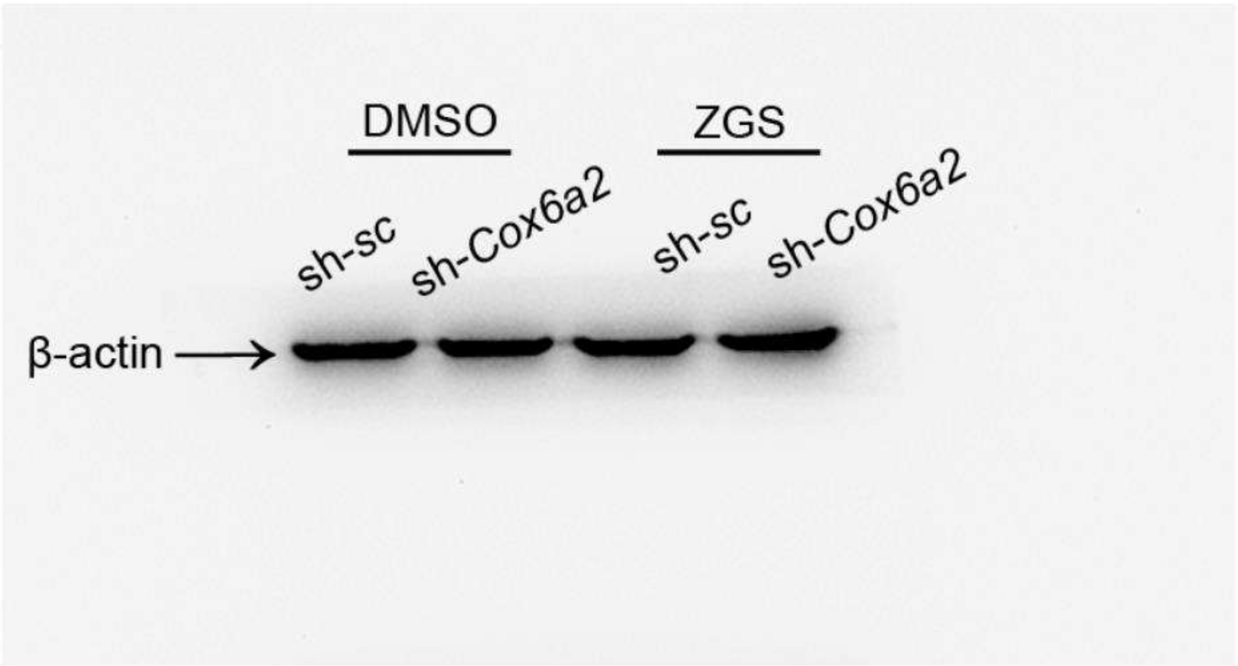
DMSO
ZGS
sh-sc
sh-Cox6a2
sh-sc
sh-Cox6a2
β-actin

Supplementary Fig. S1

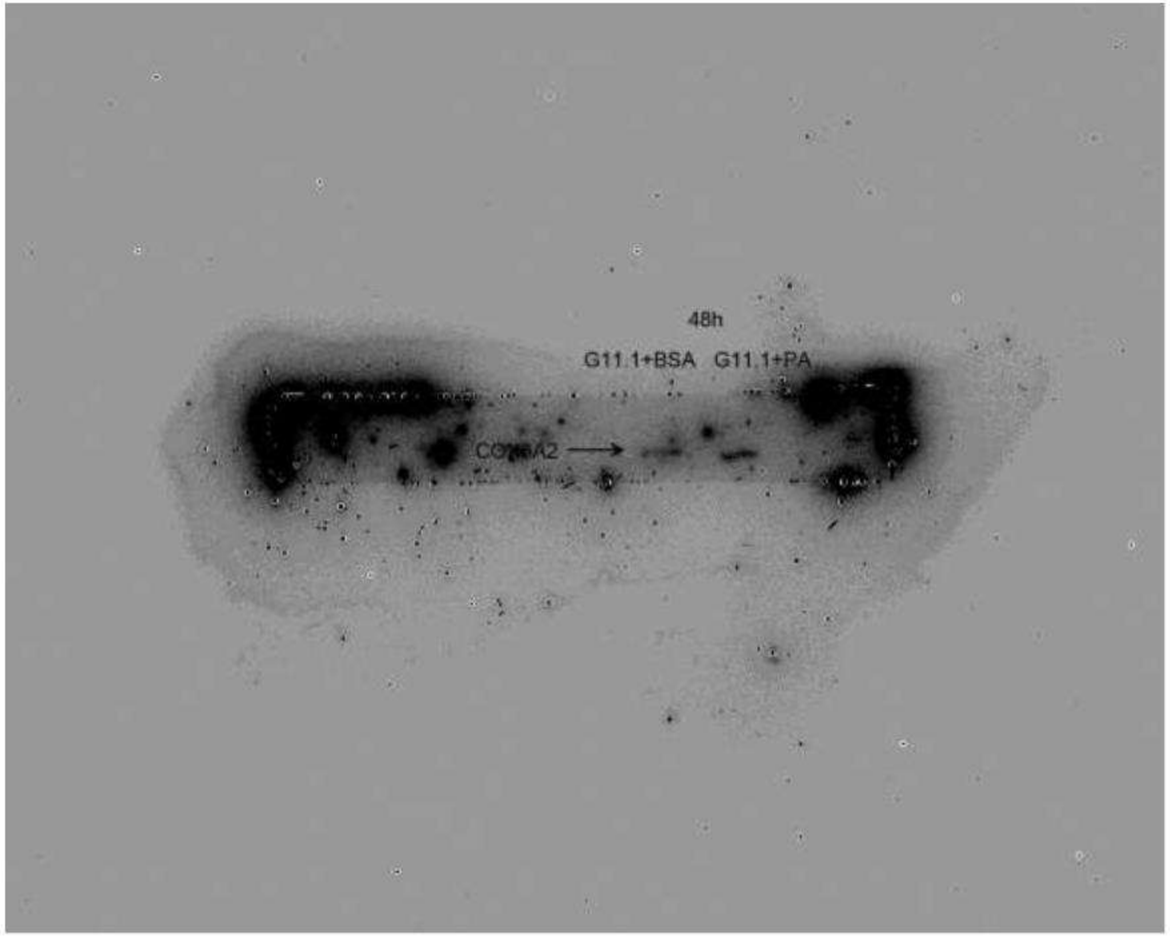

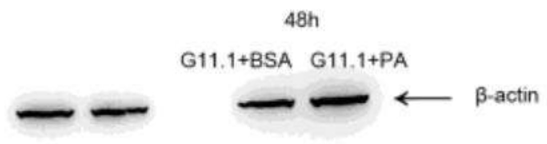

Supplementary Fig. S2A

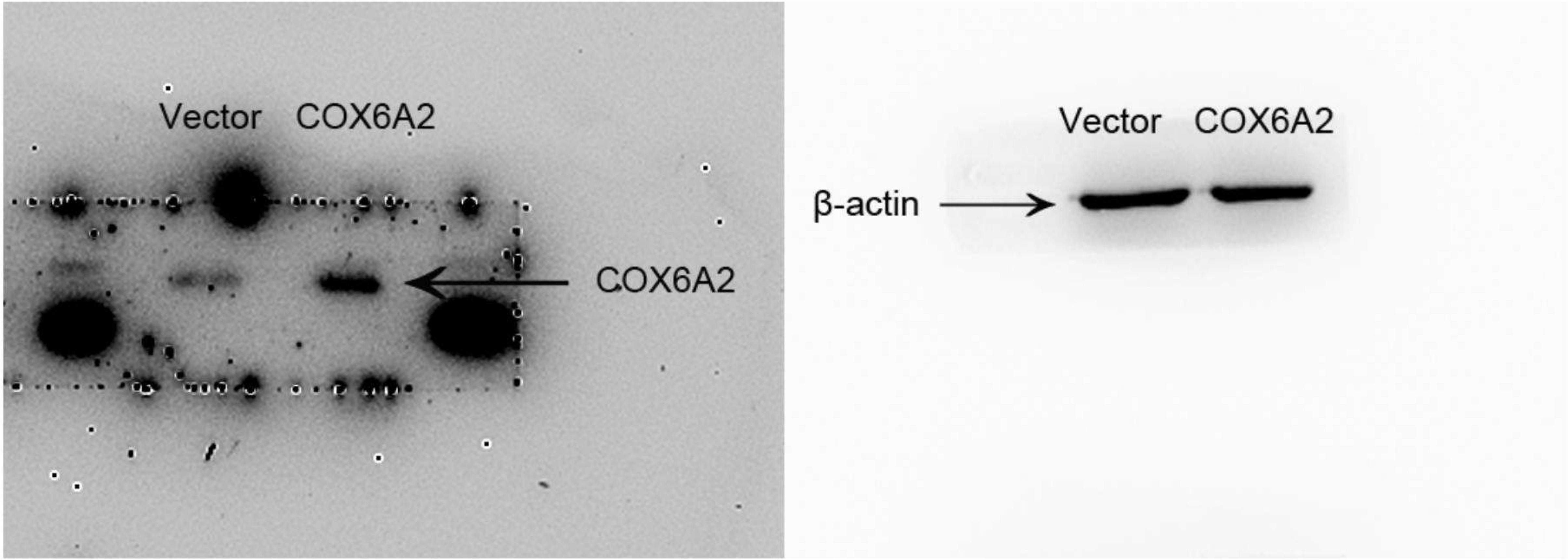

Supplementary Fig. S2B

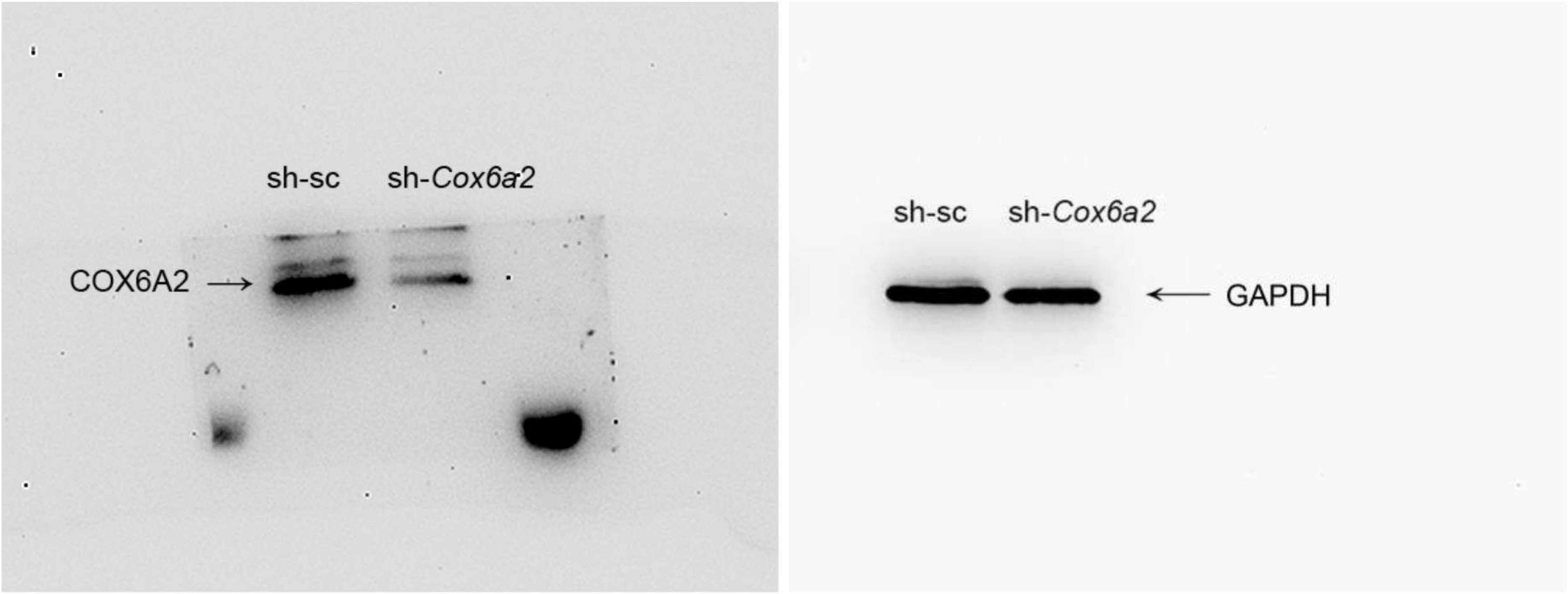

Supplementary Fig. S2C

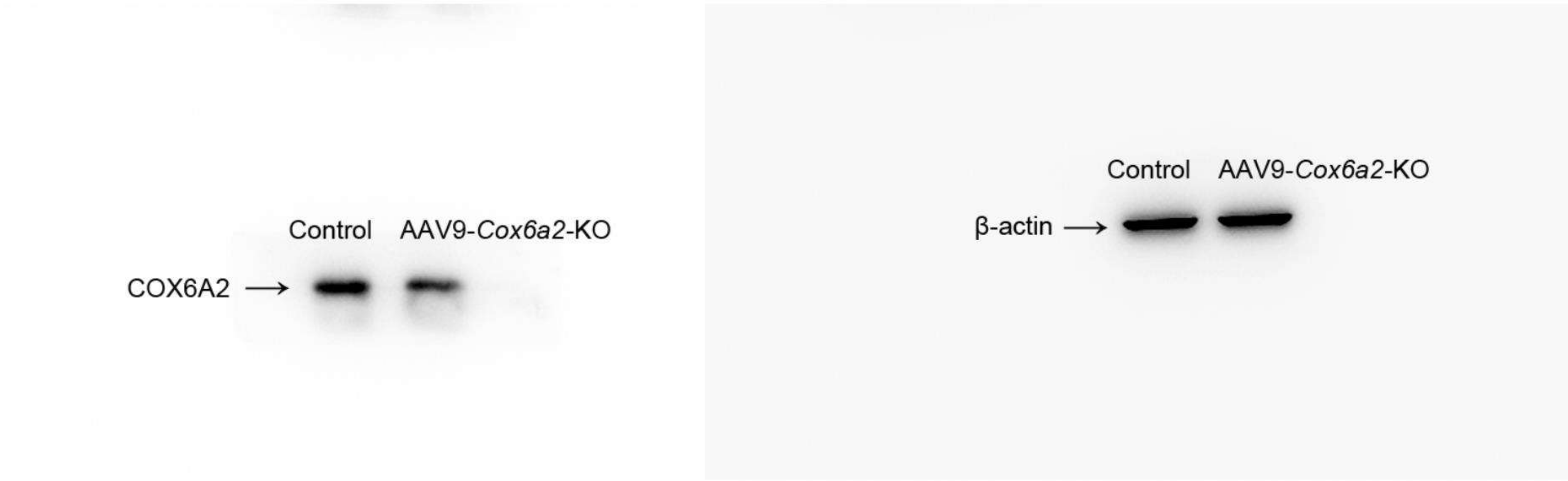

Supplementary Fig. S2D

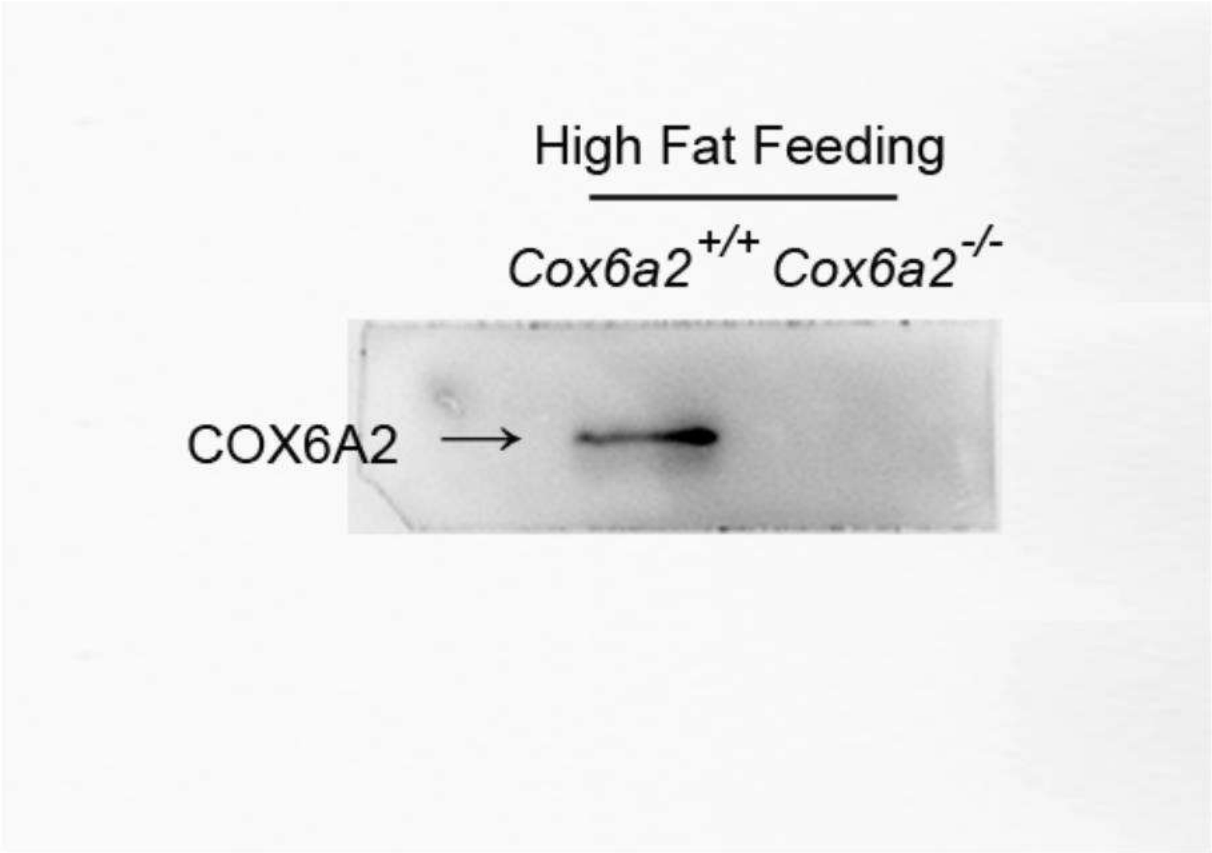

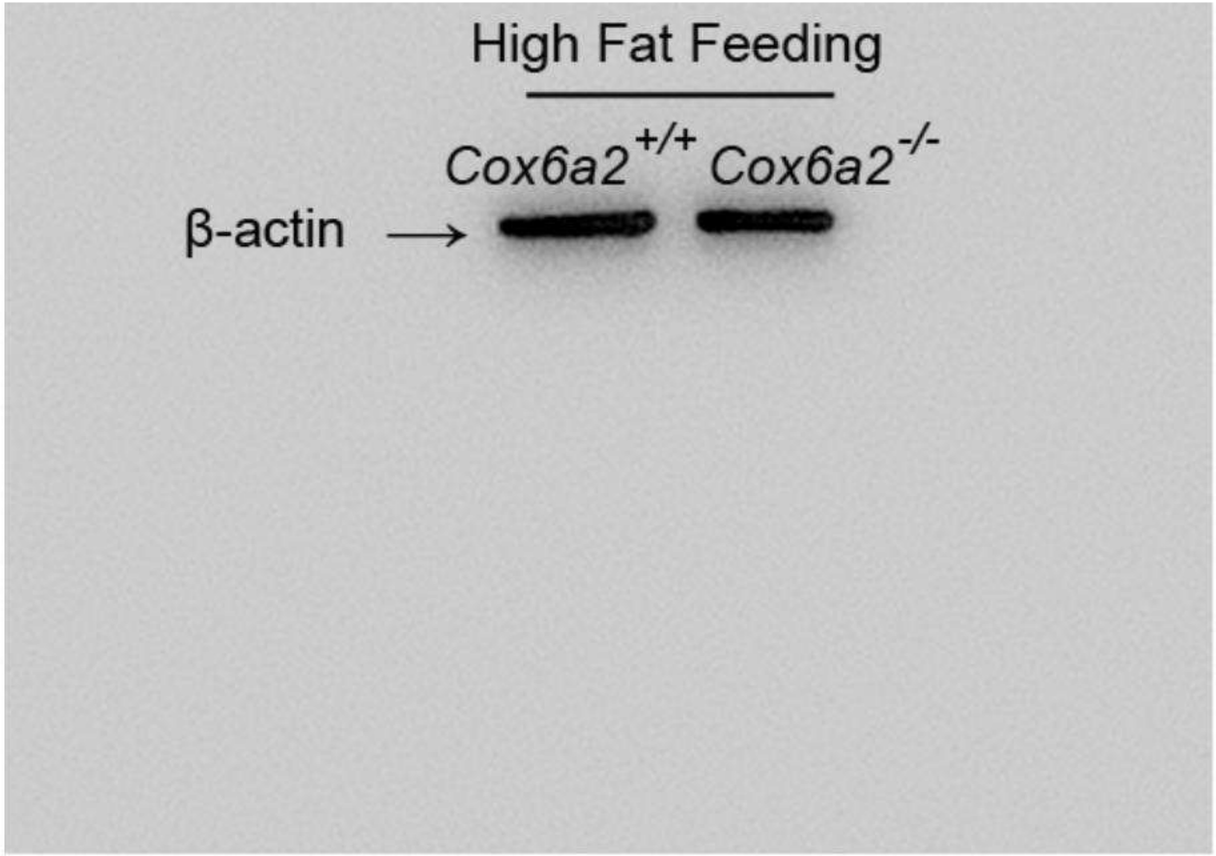

Supplementary Fig. S4

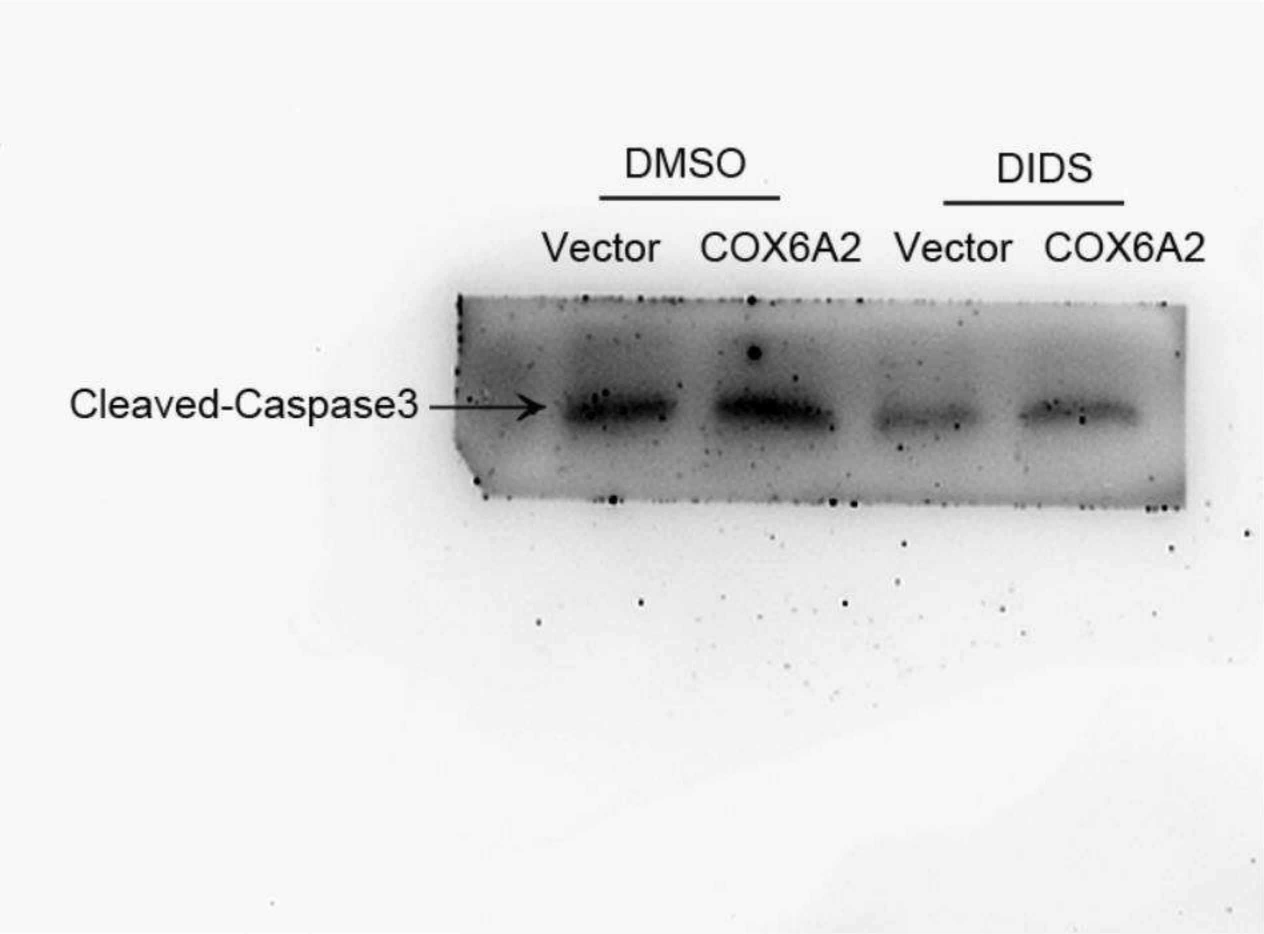

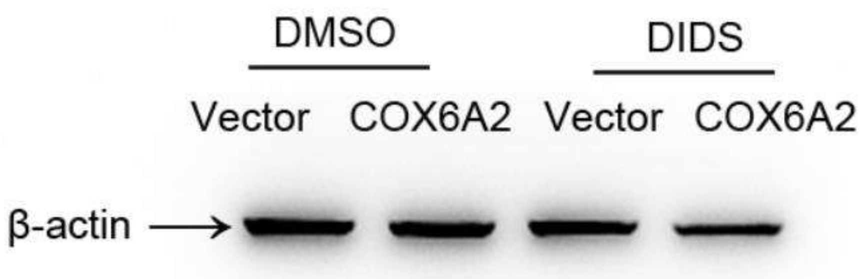

Supplementary Fig. S5B

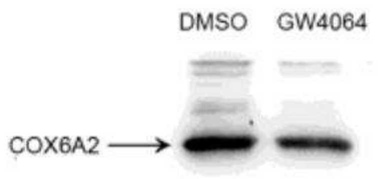

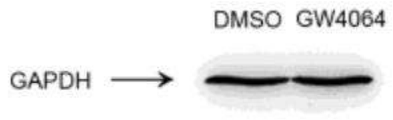

Supplementary Fig. S6B

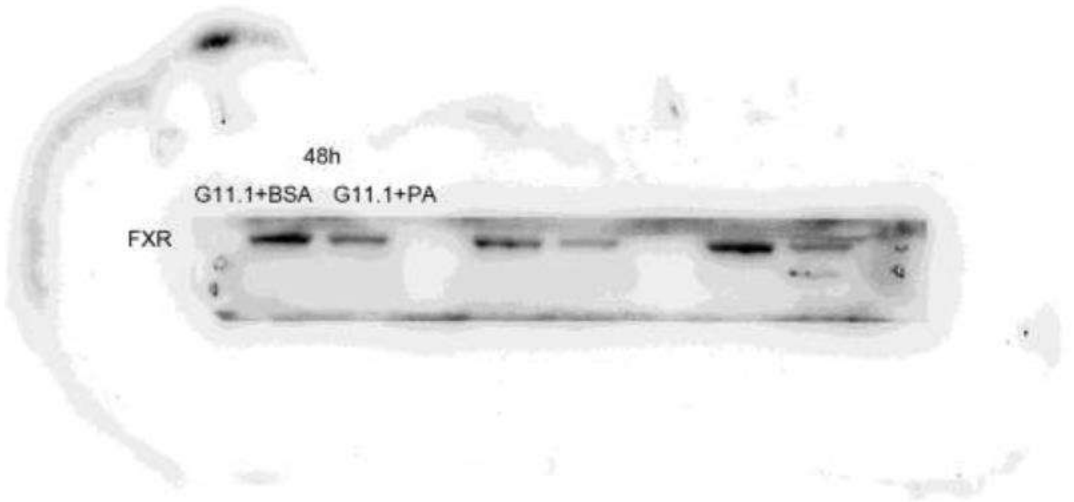

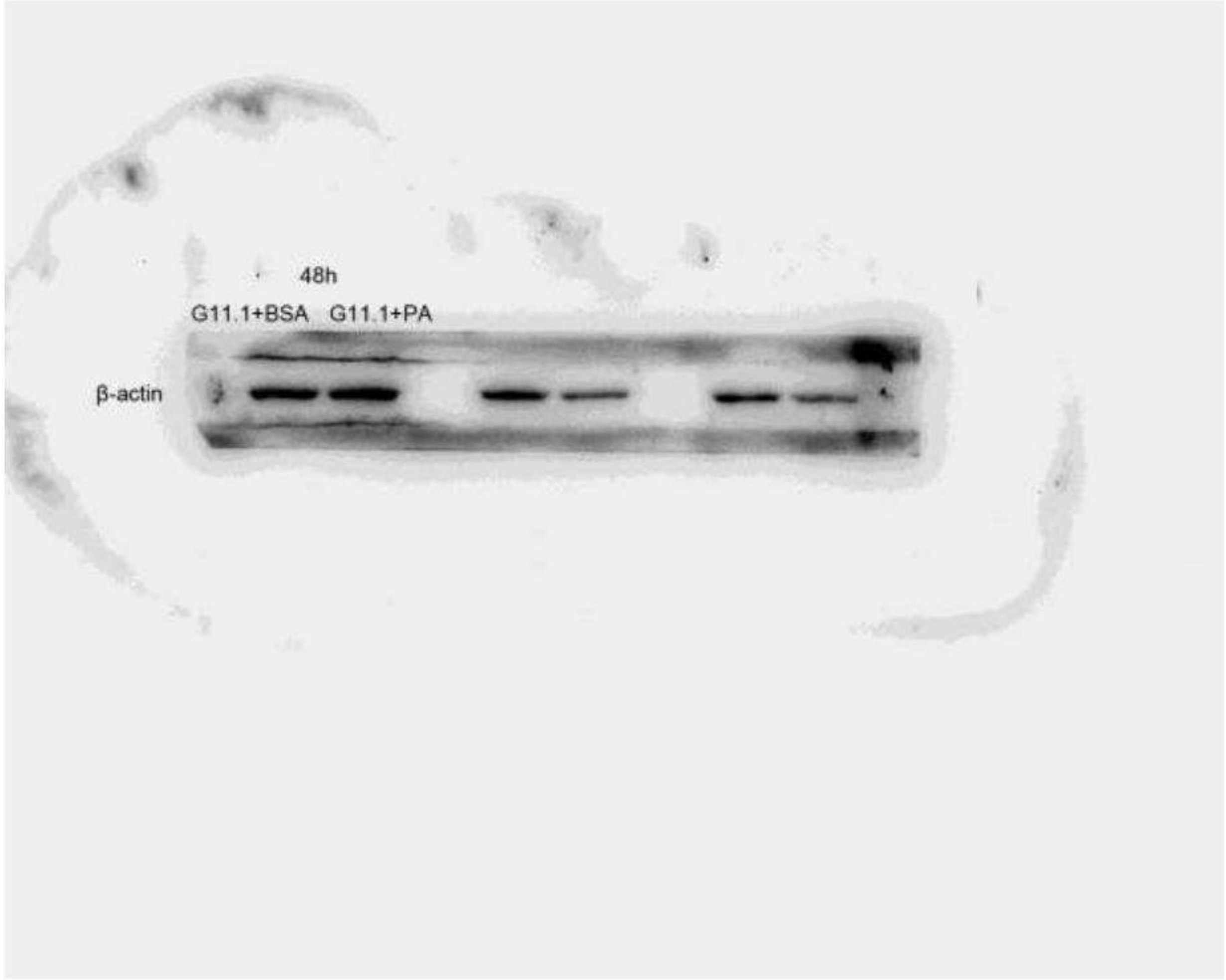

Supplementary Fig. S7A

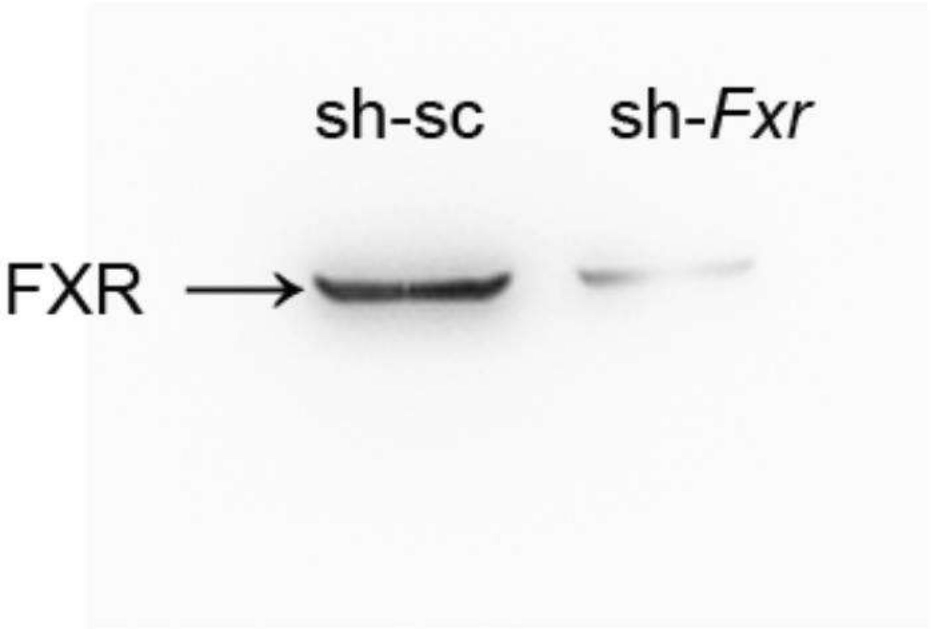

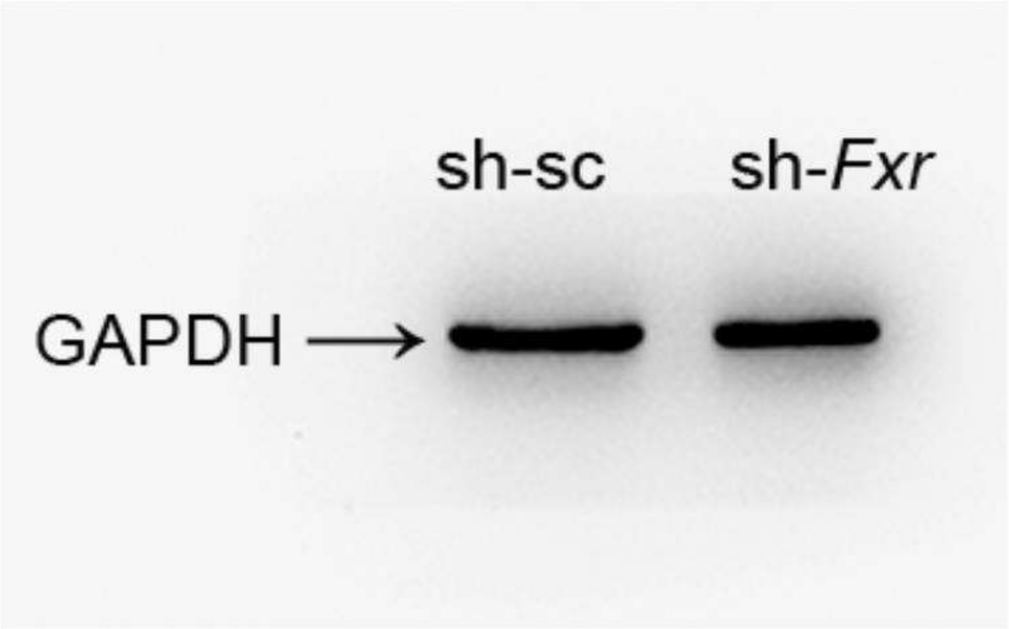

Supplementary Fig. S7B

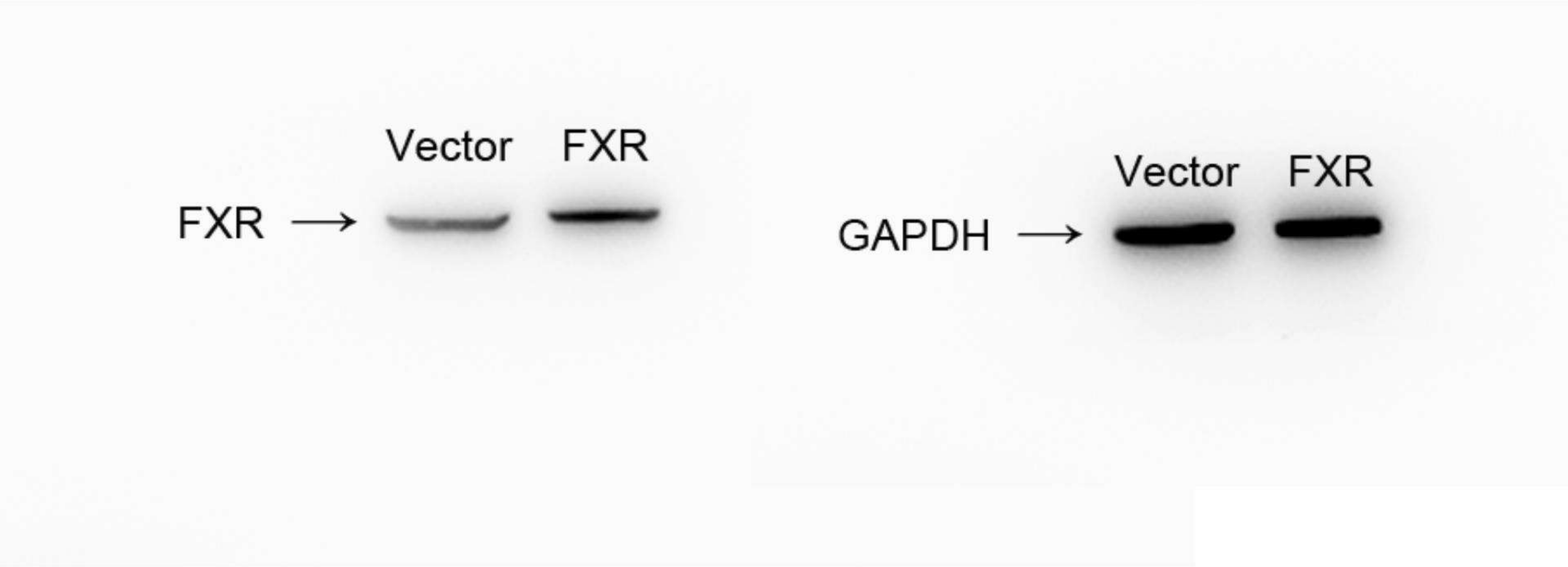

Supplementary Fig. S8A

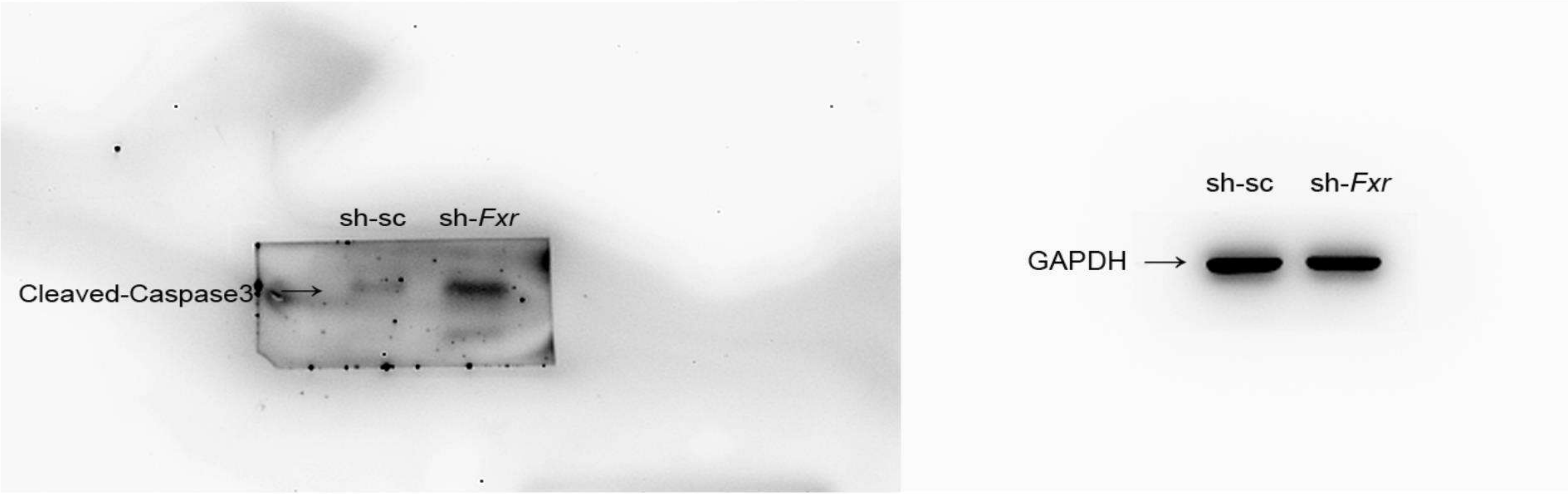

Supplementary Fig. S8C

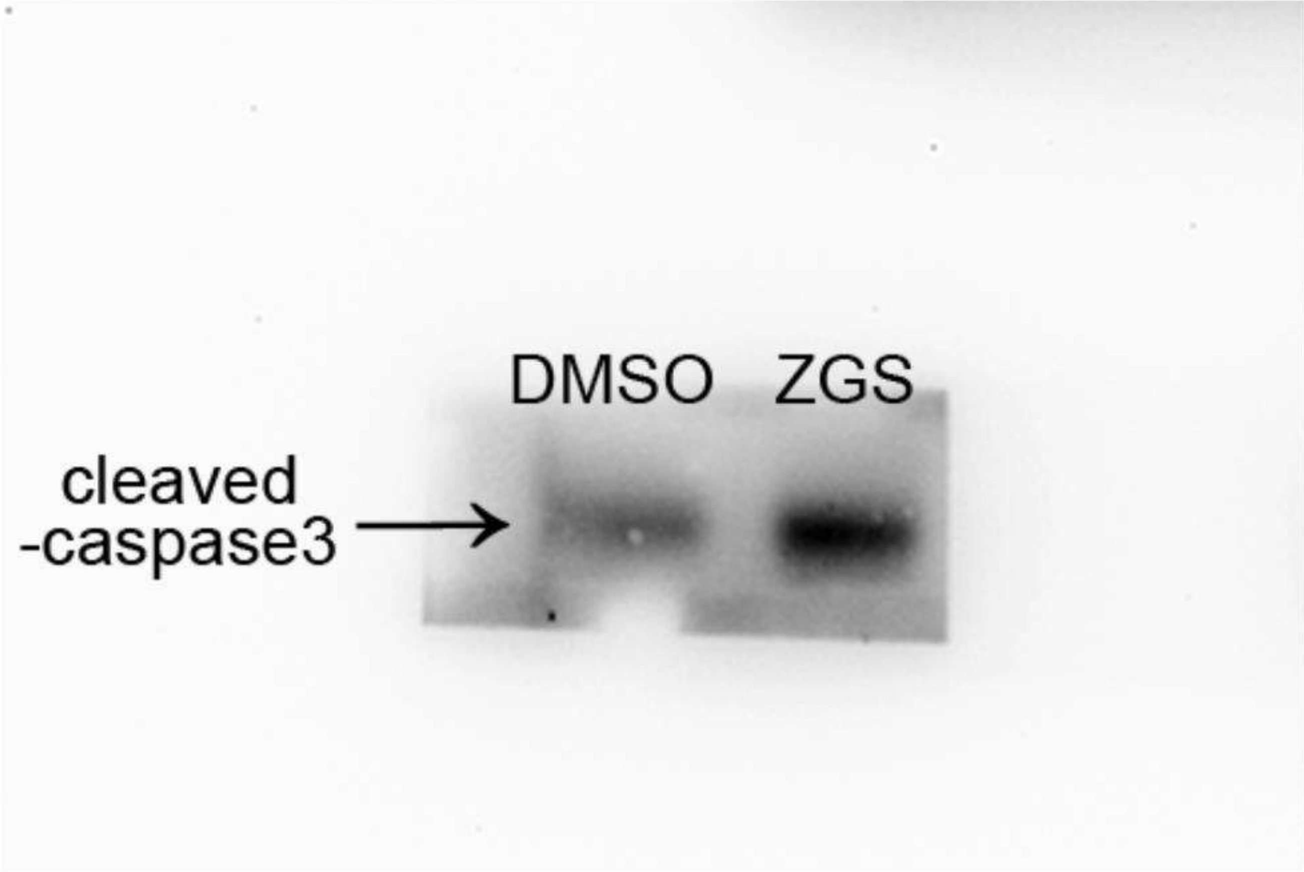

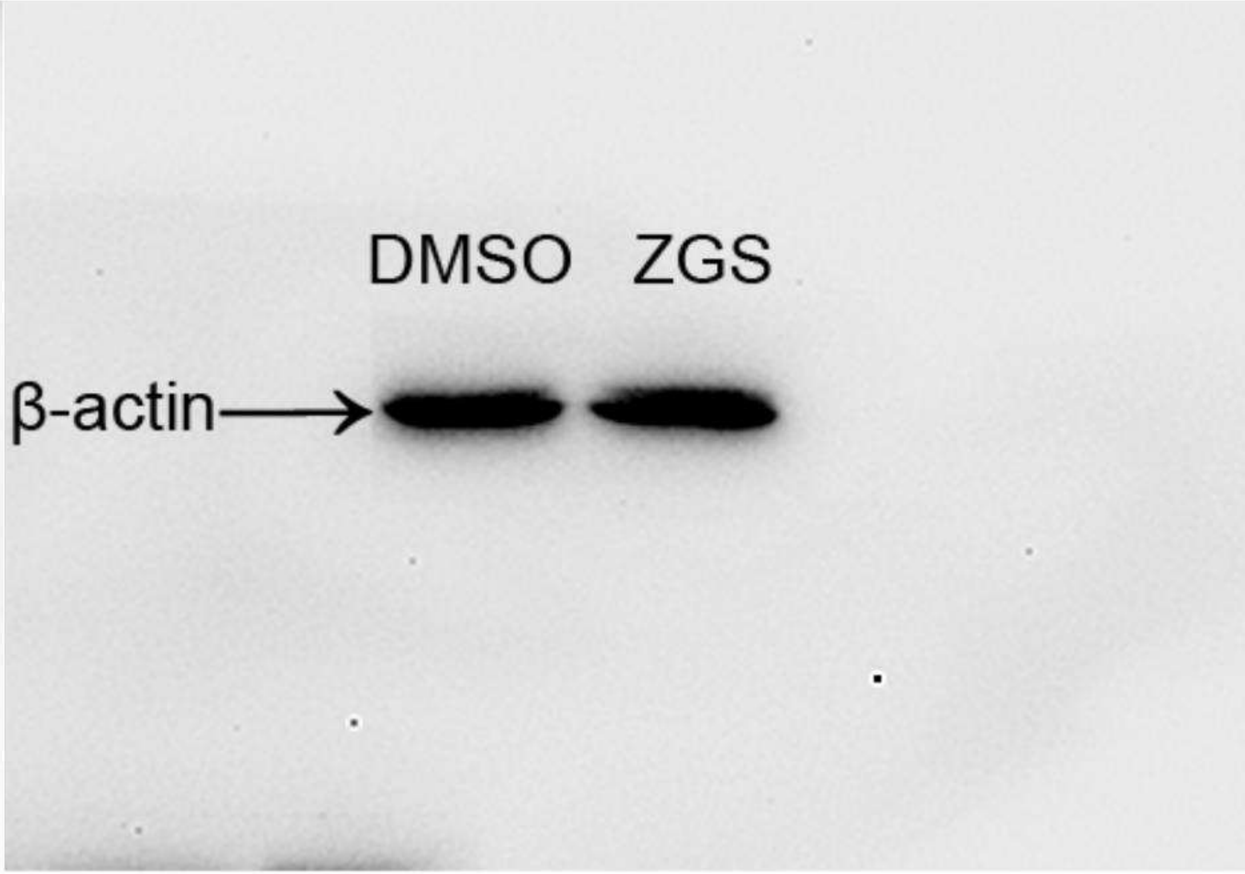

Supplementary Fig. S8D

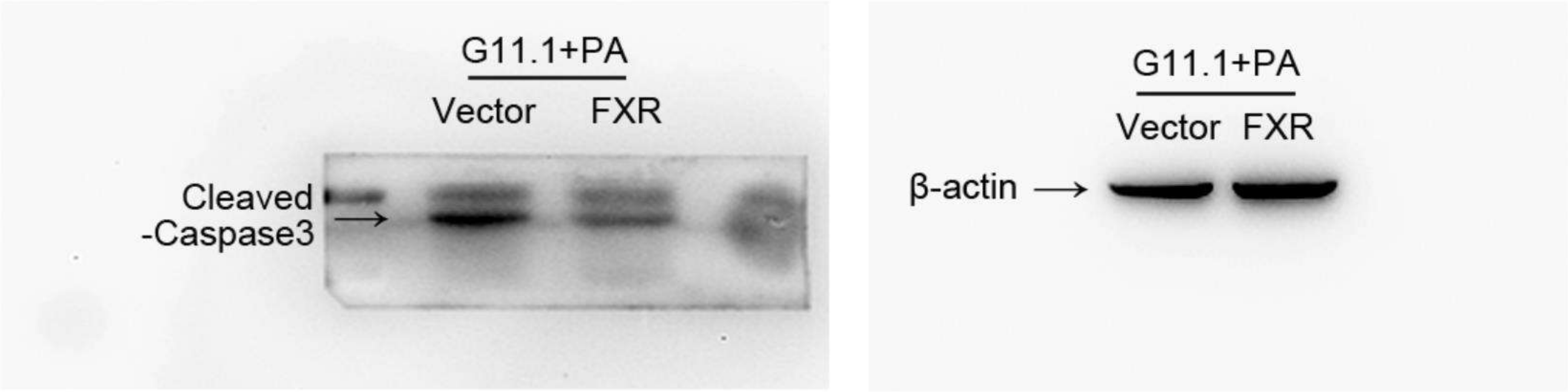

Supplementary Fig. S9

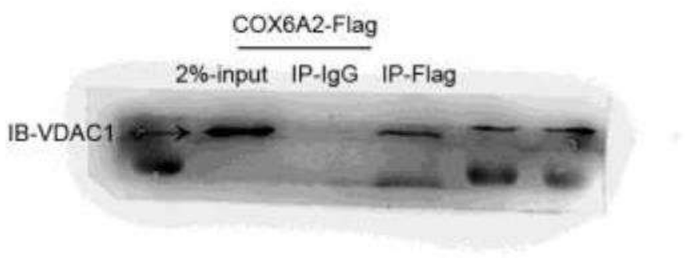

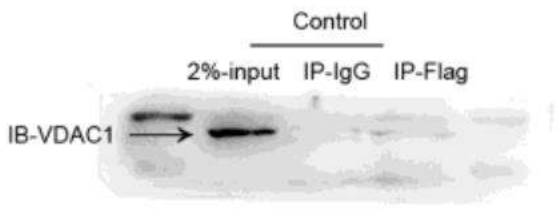

Supplementary Fig. S9

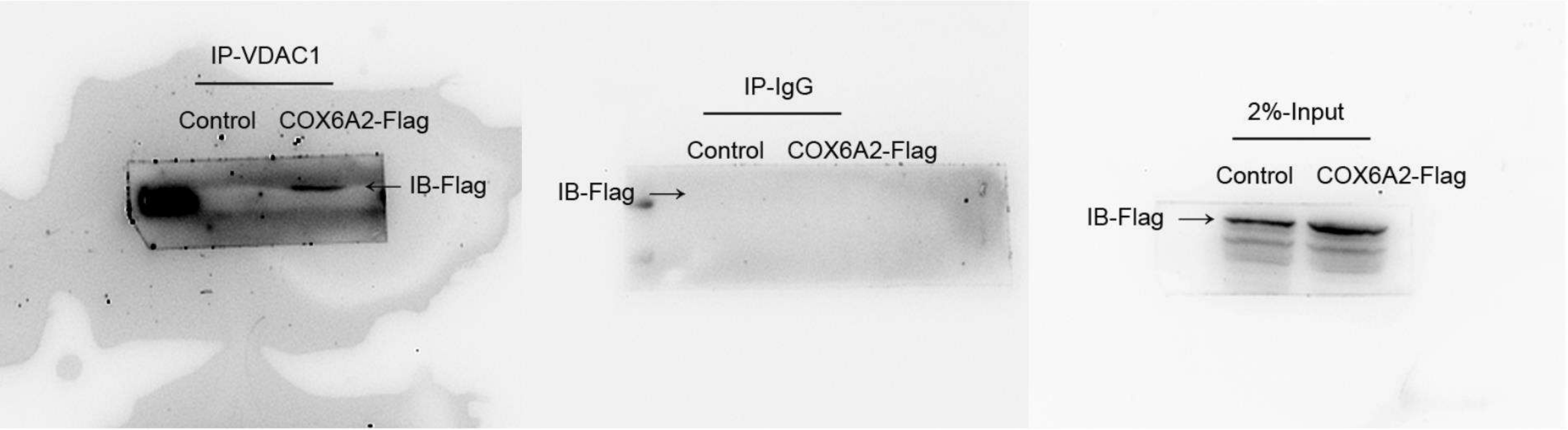

Supplement: Supplementary file 11 — Supplemental Material 2 [file 41419_2024_7302_MOESM11_ESM.pdf]
