# Supplementary material for: FXR-regulated COX6A2 triggers mitochondrial apoptosis of pancreatic β-cell in type 2 diabetes
Source: Cell Death Dis. 2024 Dec 20;15(12):920. doi: 10.1038/s41419-024-07302-4 (PMC11659401; doi:10.1038/s41419-024-07302-4)
Supplement: Supplementary file 13 — Supplementary Table 1 [file 41419_2024_7302_MOESM13_ESM.docx]

**Table 1. List of primers used for real-time PCR**

| Gene | Primers |  |
| --- | --- | --- |
| *Bax* | Forward 5’-GCAAACTGGTGCTCAAGG-5’  Reverse 5’-GGTCCCGAAGTAGGAAAGG-3’ | |
| *Vdac1* | Forward 5’- CTTGGCTATGAGGGTTGG -3’  Reverse 5’- CCTGATACTTGGCTGCTATT -3’ | |
| *Cox6a2*  *Gapdh* | Forward 5'- TGACCTTTGTGCTGGCTCT -3'  Reverse 5'- GAAGGGCTTGGTTCGGAT -3'  Forward 5'- CCTTCATTGACCTCAACTAC -3'  Reverse 5'- TCGCTCCTGGAAGATGGTGAT -3' | |
| *Actin*  (beta) | Forward 5'-GTAAAGACCTCTATGCCAACA-3'  Reverse 5'-GGACTCATCGTACTCCTGCT-3' | |
