# Supplementary material for: FXR-regulated COX6A2 triggers mitochondrial apoptosis of pancreatic β-cell in type 2 diabetes
Source: Cell Death Dis. 2024 Dec 20;15(12):920. doi: 10.1038/s41419-024-07302-4 (PMC11659401; doi:10.1038/s41419-024-07302-4)
Supplement: Supplementary file 14 — Supplementary Table 2 [file 41419_2024_7302_MOESM14_ESM.docx]

**Table 2. List of primers used for ChIP assay**

| Gene | Primers |  |
| --- | --- | --- |
| *Cox6a2 promoter* | Forward 5’-ACACGATGGCGGTAGAGG-3’  Reverse 5’-GATGGCTCAGCGGTAAGG-3’ | |
